# Intermolecular 1,2,4-Thiadiazole Synthesis Enabled by Enzymatic Halide Recycling with Vanadium-Dependent Haloperoxidases

**DOI:** 10.1021/jacs.5c01175

**Published:** 2025-03-12

**Authors:** Manik Sharma, Cameron A. Pascoe, Stacey K. Jones, Sophia G. Barthel, Katherine M. Davis, Kyle F. Biegasiewicz

**Affiliations:** + Department of Chemistry, Emory University, Atlanta, Georgia 30322, United States; ° School of Molecular Sciences, Arizona State University, Tempe, Arizona 85281, United States

## Abstract

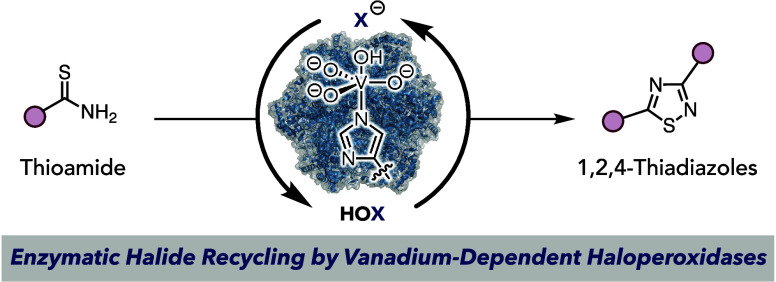

The enzymatic synthesis of heterocycles is an emerging biotechnology for the sustainable construction of societally important molecules. Herein, we describe an enzyme-mediated strategy for the oxidative dimerization of thioamides enabled by enzymatic halide recycling by vanadium-dependent haloperoxidase enzymes. This approach allows for intermolecular biocatalytic bond formation using a catalytic quantity of halide salt and hydrogen peroxide as the terminal oxidant. The established method is applied to a diverse range of thioamides to generate the corresponding 1,2,4-thiadiazoles in moderate to high yields with excellent chemoselectivity. Mechanistic experiments suggest that the reaction proceeds through two distinct enzyme-mediated sulfur halogenation events that are critical for heterocycle formation. Molecular docking experiments provide insight into reactivity differences between biocatalysts used in this study. Finally, the developed biocatalytic oxidative dimerization is applied to a preparative scale chemoenzymatic synthesis of the anticancer agent penicilliumthiamine B. These studies demonstrate that enzymatic halide recycling is a promising platform for intermolecular bond formation.

## Introduction

**Figure 1:**
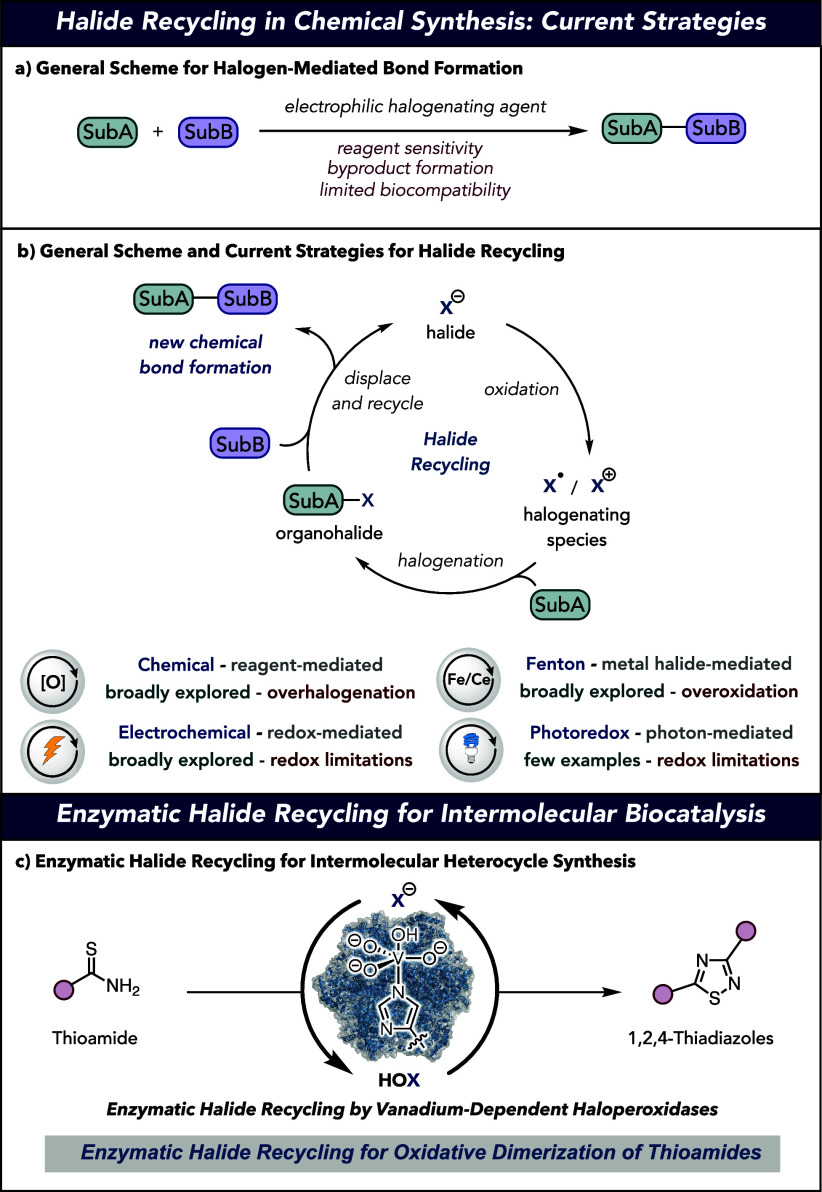
(a) General scheme for halogenation-mediated bond formation. (b) Halide recycling strategies in chemical synthesis. (c) Proposed enzymatic halide recycling strategy for intermolecular halogenation-mediated bond formation.

Halogenation-induced bond formation is an emerging approach for the synthesis of molecules across numerous chemical industries. This reaction design strategy relies on the use of an electrophilic halogenating agent that serves to activate an organic substrate for an ensuing carbon–carbon,^1−3^ carbon–heteroatom,^4−6^ or heteroatom–heteroatom bond formation.^7−9^ Despite the synthetic utility of established methods for halogenation-induced bond formation, electrophilic halogenating reagents are often air- and/or moisture-sensitive and substrate-scope-limiting as a result of overhalogenation events and produce undesired organic or halogenated byproducts.^10,11^ Moreover, these processes are largely bioincompatible, limiting their potential for applications in chemoenzymatic synthesis (Figure 1a). As a result, alternative strategies for catalytic *in situ* generation of reactive halogenating agents from inert halide salts remain highly desired.

The design of a catalytic halide recycling system for intermolecular bond formation necessitates control over three fundamental steps: (1) oxidation of a halide ion (X^–^, where X = Cl, Br, I, or F), providing the reactive halogenating species as a halide radical or halonium ion (X^•^ or X^+^), (2) selective halogenation of one of two substrate partners (SubA) to give an intermediate organohalide (SubA-X), and (3) a halide recycling step, wherein the second substrate (SubB) creates the desired chemical bond through displacement of the starting halide from bond formation with SubA. Traditional small molecule methods for halide recycling in chemical synthesis include chemical-,^12−14^ Fenton-,^15−17^ electrochemical-,^6,18^ and photoredox-based oxidation systems (Figure 1b).^19,20^ Despite the establishment of these methods, their continued development remains limited by undesired oxidation or halogenation events or sensitivity to redox-active functional groups.

Enzymes are an attractive alternative to established methods for halide recycling as a result of their efficiency, selectivity, and sustainability parameters.^21,22^ Among the broad range of halogenases that exist in nature,^23−25^ the vanadium-dependent haloperoxidase (VHPO) class of enzymes has recently been recognized as an emerging biocatalyst platform for chemical synthesis.^26^ These haloperoxidases isolated from marine algae, fungi, and bacteria perform catalytic oxidation of halides using hydrogen peroxide (H_2_O_2_) as the terminal oxidant.^23−25,27^ In nature, VHPOs use this mechanism to perform direct electrophilic halogenation of arenes and enolizable carbon centers,^23−25,28^ haloetherification,^29−33^ halolactonization,^34^ and polyene cyclization reactions.^28,35^ Some other notable characteristics of VHPOs include their facile recombinant expression in *E. coli*, resistance to oxidative degradation, stability in organic solvents, and tolerance to high temperatures, making their reaction parameter versatility remarkably valuable for biocatalyst development.^36,37^

While important seminal examples of enzymatic halide recycling have been reported in the context of an aza-Achmatowicz reaction by Hollmann et al.,^38^ decarboxylative oxidation of amino acids by Sanders et al.,^39^ and decarboxylative bromooxidation in our laboratory,^40^ they are limited to intramolecular reaction manifolds with strictly the vanadium chloroperoxidase from *Curvularia inaequalis* (*Ci*VCPO),^41^ leaving an entryway into applications of this technology to intermolecular reactions elusive. The successful demonstration of enzymatic halide recycling for intermolecular reactions would dramatically expand its potential application in chemoenzymatic synthesis. We recently hypothesized that enzymatic halide recycling could be applied to the intermolecular biocatalytic bond formation. In this process, a VHPO would be responsible for repetitive halide oxidation to generate the electrophilic halogenating agent in the form of hypohalous acid (HOX) using H_2_O_2_ as the stoichiometric oxidant. As a proof of principle, we envisioned applying this strategy to biocatalytic heterocycle synthesis in the context of an oxidative dimerization of thioamides to generate 1,2,4-thiadiazoles, an increasingly important class of heterocycles in the pharmaceutical and agricultural industries (Figure 1c).^42−44^ While numerous strategies have been developed for the oxidative dimerization of thioamides,^45^ these methods rely on strongly oxidizing reagents that are both substrate-scope-limiting and hazardous from an operational standpoint. Herein, we report that VHPOs are viable biocatalysts for the oxidative dimerization of thioamides.

## Results and Discussion

**Figure 2:**
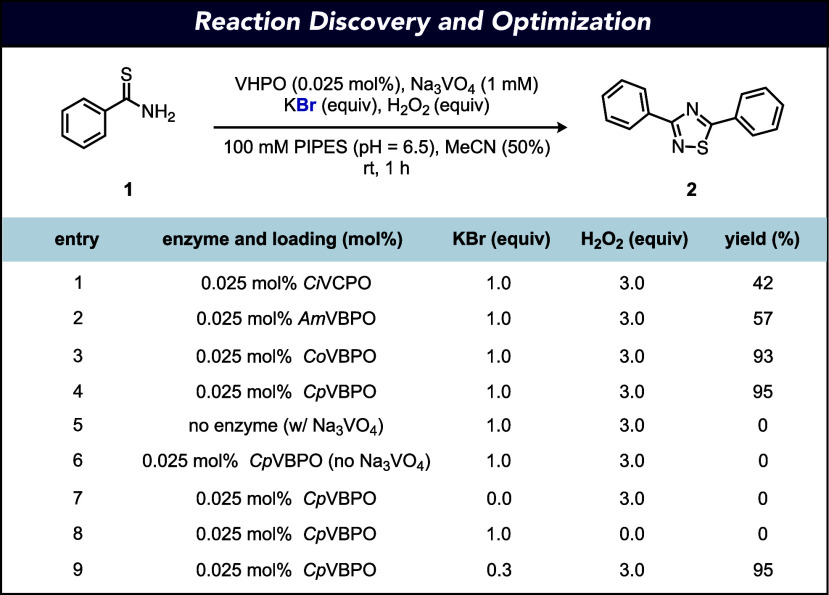
Reaction discovery and optimization for the VHPO-catalyzed oxidative dimerization of thioamides. Reaction conditions: **1** (4.0 μmol, 0.6 mg), VHPO (0.025 mol %), Na_3_VO_4_ (1 mM), KBr (0.3–1.0 equiv), H_2_O_2_ (3.0 equiv), PIPES buffer (100 mM, pH = 6.5, 200 μL), MeCN (500 μL), 1.0 mL total reaction volume, 1 h, rt. Yields were determined by HPLC based on a calibration curve. See the Supporting Information for details.

Our studies were initiated by examining the oxidative dimerization of thiobenzamide (**1**) to produce 3,5-diphenyl-1,2,4-thiadiazole (**2**) with the chloroperoxidase from *Curvularia inaequalis* (*Ci*VCPO) on the basis of its broad documented synthetic utility in performing halogenation reactions.^26,38−40,46−49^ Subjection of **1** to *Ci*VCPO (0.025 mol %), sodium orthovanadate (Na_3_VO_4_, 1 mM), potassium bromide (KBr, 1.0 equiv), and H_2_O_2_ (3.0 equiv) in 1,4-piperazinediethanesulfonic acid (PIPES) buffer (100 mM, pH = 6.5) and MeCN as cosolvent (50% v/v) provided 1,2,4-thiadiazole **2** in 42% yield in 1 h (Figure 2, entry 1). To identify a more suitable biocatalyst for oxidative dimerization, a collection of structurally diverse vanadium bromoperoxidases (VBPOs) from *Acaryochloris marina* (*Am*VBPO),^50^*Corallina officinalis* (*Co*VBPO),^51^ and *Corallina pilulifera* (*Cp*VBPO)^52^ were investigated under the same reaction conditions. All VBPOs used outperformed *Ci*VCPO, providing **2** in 57%, 93%, and 95% yields, respectively (Figure 2, entries 2–4). Control reactions were run to ensure the necessity of all reaction components, including enzyme (*Cp*VBPO), Na_3_VO_4_, KBr, and H_2_O_2_ (Figure 2, entries 5–8). Gratifyingly, KBr loadings could be reduced to as low as 0.3 equiv without diminishing reaction performance (Figure 2, entry 9). Changing the halide source to potassium chloride (KCl) led to no reaction. Other notable features of this catalyst system include its tolerance of increased H_2_O_2_ loadings up to 8.0 equiv without significant diminishment in yield (Supplementary Figure S1) and its versatile performance in a range of organic cosolvents (Supplementary Figure S2). Notably, reducing the cosolvent loading (v/v) to lower than 40% led to a decrease in yield (Figure S3). We currently attribute changes in reaction performance to product solubility in the reaction.

**Figure 3:**
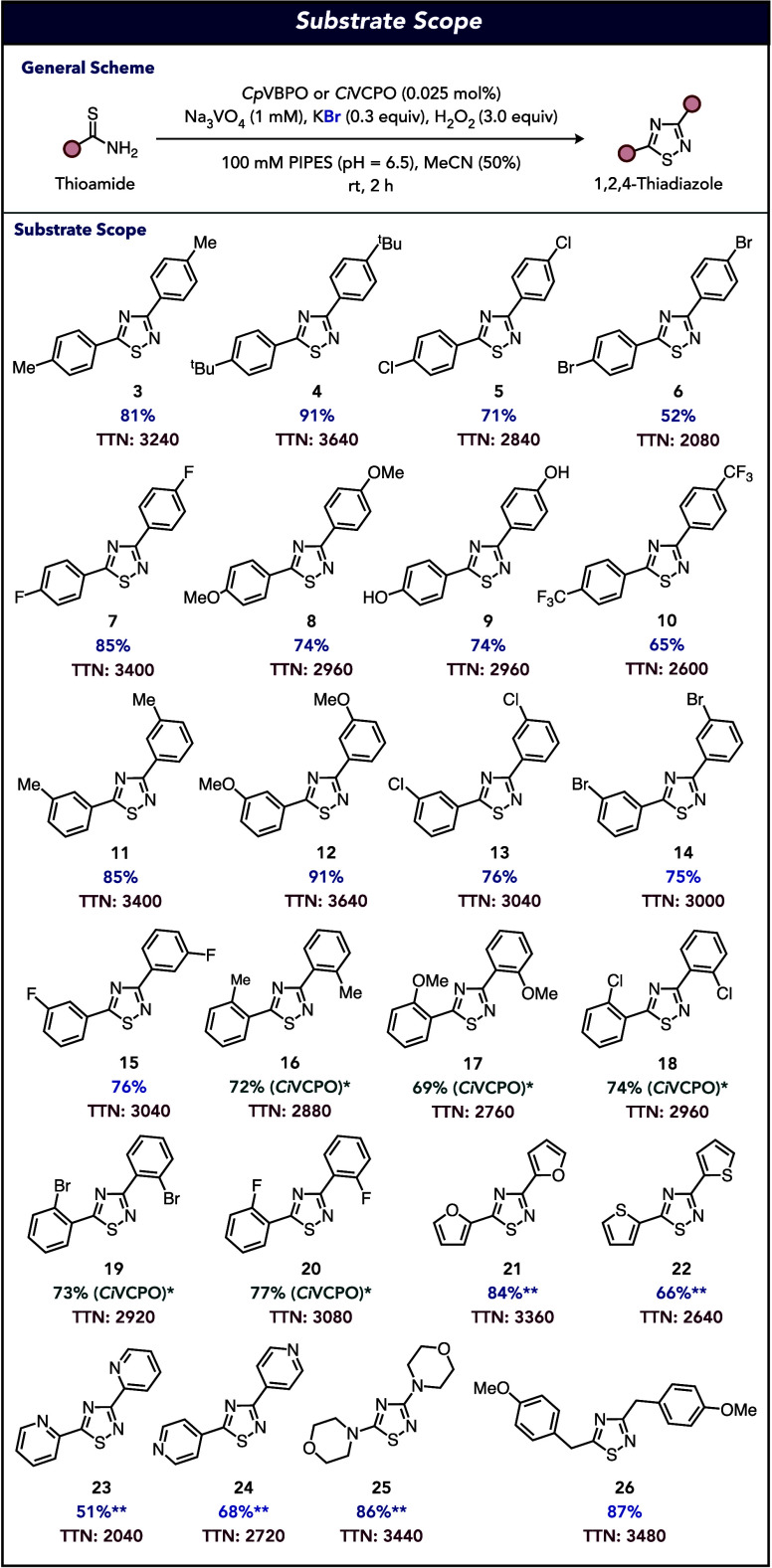
Substrate scope for the VHPO-catalyzed oxidative dimerization of thioamides. Reaction conditions: substrate (0.8 mmol), *Cp*VBPO (0.025 mol %), Na_3_VO_4_ (1 mM), KBr (0.3 equiv), H_2_O_2_ (3.0 equiv), PIPES buffer (100 mM, pH = 6.5), MeCN (50%), 2 h, rt. *CiVCPO (0.025 mol %), Na_3_VO_4_ (1 mM), KBr (0.3 equiv), H_2_O_2_ (3.0 equiv), citrate buffer (100 mM, pH = 5), MeCN (50%), 2 h, rt. **CpVBPO (0.025 mol %), Na_3_VO_4_ (1 mM), KBr (3.0 equiv), H_2_O_2_ (3.0 equiv), citrate buffer (100 mM, pH = 5), MeCN (50%), 2 h, rt. All indicated yields are isolated yields. TTNs were determined by dividing the quantity of the resulting product by the concentration of the enzyme used. See the Supporting Information for more details.

With the establishment of the optimized conditions for *Cp*VBPO-catalyzed oxidative dimerization, the substrate scope of the reaction was interrogated. For *para*-substituted aromatic thiobenzamides, the catalyst system tolerates alkyl substitution including methyl- and *tert*-butyl groups (Figure 3, **3**–**4**), producing the corresponding 1,2,4-thiadiazoles in 81% and 91% yields with total turnover numbers (TTNs) of 3240 and 3640, respectively. A noticeable decrease in reaction performance is observed for chlorine- (71% yield, 2840 TTN) and bromine-containing (52%, 2080 TTN) substrates (Figure 3, **5**–**6**). Interestingly, a *para*-fluorine-substituted thiobenzamide undergoes oxidative dimerization in high yield and TTN (85% yield, 3400 TTN) (Figure 3, **7**). The catalyst system also operates on *para*-methoxy- and hydroxy-substituted thiobenzamides, both of which proceed in 74% yield, with 2960 TTN (Figure 3, **8**–**9**) and no overhalogenation products observed. Finally, a trifluoromethyl-substituted thioamide is obtained in 65% yield (2600 TTN). We next explored *meta*-substitution on the thiobenzamide to find that electron-donating methyl- (85% yield, 3400 TTN) and methoxy-substitution (91%, 3640 TTN) (Figure 3, **11**–**12**) is well tolerated. Halogen substitution is also accommodated on the arene across chlorine, bromine, and fluorine substituents ranging from 75 to 76% yields and 3000–3040 TTNs (Figure 3, **13**–**15**). Despite the effectiveness of *Cp*VBPO as a catalyst for *para*- and *meta*-substituted thiobenzamides, *ortho*-substituted thioamides performed in drastically lower yields (<5%). To address this synthetic limitation, we again interrogated our collection of VHPOs (Figure S4) and found that *Ci*VCPO was a viable catalyst for these substrates. With this new discovery, the oxidative dimerization protocol was extended to methyl-, methoxy-, chlorine-, bromine-, and fluorine-substitution at the *ortho*-position in yields ranging from 69 to 77% and TTNs ranging from 2760 to 3080 (Figure 3, **16**–**20**). When the starting thioamide possessed an aromatic heterocycle, the reaction under standard conditions suffered from thioamide hydrolysis to the corresponding amide with *Cp*VBPO. To circumvent this limitation, reaction conditions can be simply modified by using excess KBr (3.0 equiv), enabling oxidative dimerization of furan-, thiophene-, 2-pyridyl- and 4-pyridyl-containing substrates in 51–84% yields and 2040–3360 TTNs (Figure 3, **21**–**24**). Our feature bromoperoxidase (*Cp*VBPO) was also a viable biocatalyst for oxidative dimerization of a morpholine-derived thioamide in 86% yield and 3440 TTN (Figure 3, **25**) and a *para*-methoxybenzyl-substituted thioamide in 87% yield and 3480 TTN (Figure 3, **26**). While a majority of the substrates interrogated in the study required a KBr loading of 0.3 equiv, it could be lowered to as low as 0.01 equiv for the production of **7** with *Cp*VBPO (Supplementary Figure S5) and the production of **16** with *Ci*VCPO (Supplementary Figure S6) without affecting the reaction performance.

**Figure 4:**
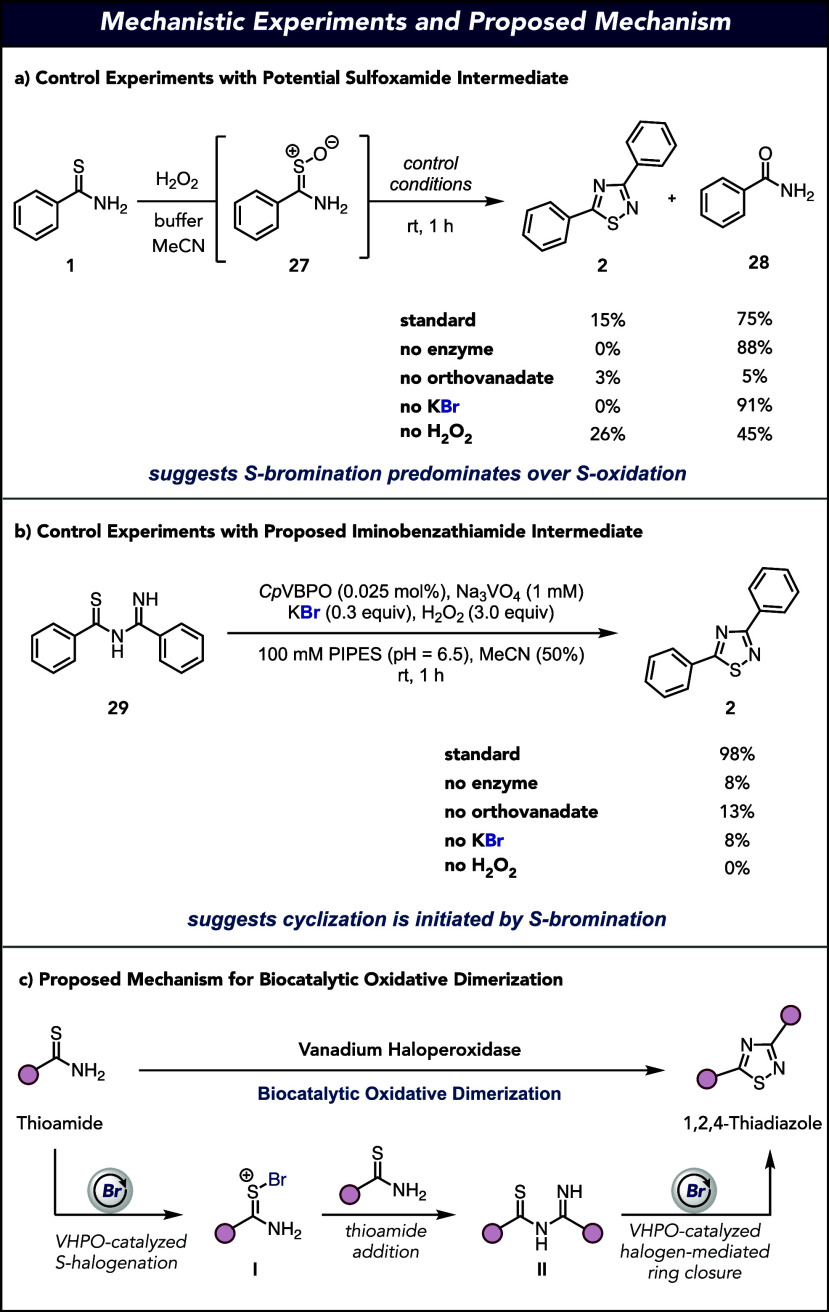
Mechanistic experiments for VHPO-catalyzed oxidative dimerization of thioamides. (a) Control experiments with possible sulfoxide intermediate. (b) Control experiments with the proposed iminobenzathiamide intermediate. (c) Proposed mechanism for VHPO-catalyzed oxidative dimerization of thioamides. See Supporting Information for more details. (c) Proposed mechanism for the VHPO-catalyzed oxidative dimerization of thioamides. See Supporting Information for more details.

Throughout the course of our investigation, we were interested in the mechanistic features of the biocatalytic oxidative dimerization of thioamides by VHPOs. It is well documented in previous reports of reagent- and catalyst-based methods that oxidative dimerization proceeds through an oxidation or halogenation event that leads to the formation of an intermediate iminobenzathiamide. This intermediate is poised for an oxidation- or halogenation-induced cyclization to generate the desired 1,2,4-thiadiazole.^45^ An intriguing observation from our original control reactions is that treatment of thiobenzamide (**1**) with H_2_O_2_ in the absence of any of the other reaction components leads to the stoichiometric generation of sulfoxamide **27**. To gain insight into whether the primary reaction mechanism proceeds through a sulfoxamide intermediate, a series of control experiments were run after *in situ* generation of **27** with H_2_O_2_. On subjection of **27** to standard reaction conditions, only 15% of the desired 1,2,4-thiadiazole was produced, suggesting that while a sulfoxamide-mediated pathway is possible with all reaction components present, oxidative dimerization proceeds predominately through S-bromination of the starting thioamide. This observation is consistent with a previous report of oxidative dimerization facilitated by treatment with H_2_O_2_ that requires more activated thioamides for obtaining moderate to high yields.^53^ A set of reaction controls were run on **27**, in which each of the reaction components was left out in sequence. When *Cp*VBPO is excluded from the reaction, the only product observed is benzamide (**28**), resulting from sulfoxamide hydrolysis. In the remainder of the control experiments, significant quantities of **28** are observed, except when orthovanadate is excluded (Figure 4a). Research on the nature of this vanadate-catalyzed hydrolysis is still underway in our laboratory. Collectively, these data suggest that reaction initiation occurs primarily through S-bromination of the starting thioamide. We next turned to confirmation of the reaction proceeding through iminobenzathiamide (**29**) through an independent synthesis.^54^ Subjection of **29** to standard reaction conditions and controls revealed that all components were required to achieve a 98% yield (Figure 4b). These results collectively suggest that the reaction (1) proceeds through the proposed iminobenzathiamide **29** and (2) undergoes S-bromination for efficient cyclization to 1,2,4-thiadizaole **2**. Based on these studies, our proposed mechanism begins with VHPO-catalyzed S-bromination of the starting thioamide, producing S-bromothioamide **I**. This intermediate is now activated for the addition of another equivalent of the starting thioamide, generating iminobenzathiamide **II**. Finally, VHPO-catalyzed bromination of **II** initiates ring closure and tautomerization to provide the desired 1,2,4-thiadiazole (Figure 4c).

**Figure 5:**
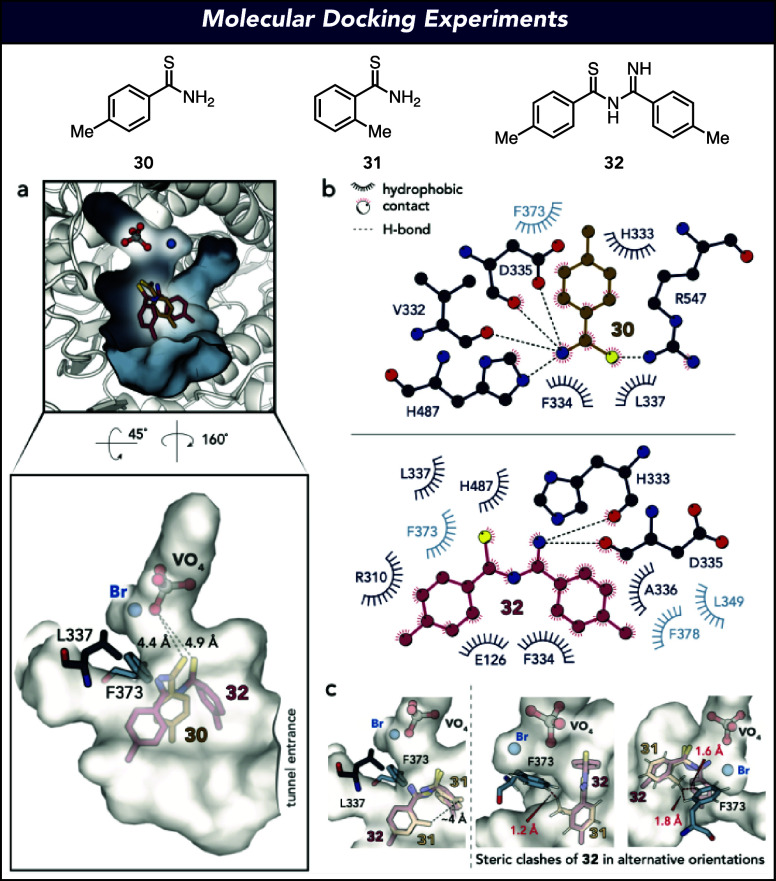
Molecular docking simulations of binding of the substrate to *Cp*VBPO. (a) The active site is formed by two monomeric subunits whose respective surfaces are depicted in dark and light blue. (inset) Rotated view visualizing **30** and **32** docked to the active site structure observed after bromide binding. While not included in the docking simulations, vanadate and bromide ions derived from PDB accession code 7QWI are included to aid in visualization. (b) Two-dimensional interaction diagrams depicting the docking contacts between substrate **30**, as well as the intermediate iminobenzathiamide **32**, and *Cp*VBPO. (c) Alignments of **31** to the docking model of **32**. (left) The only unhindered conformations of **31** that could form the iminobenzathiamide intermediate. (right) Visualizations of steric clashes with Phe373 in alternative orientations. In all panels, residues from the different monomeric subunits are colored light or dark blue. Note that the hydrogens depicted are implicit.

To provide insight into substrate binding by both *Cp*VBPO and *Ci*VCPO, we turned to molecular docking. Our primary focus was providing an explanation for the minimal activity observed for thioamides possessing *ortho*-substitution on the arene when using *Cp*VBPO. As a model comparison, we pursued docking studies with the representative reactive substrate, 4-methylthiobenzamide (**30**, 81% yield) and an electronically similar but unreactive *ortho*-substituted analogue, 2-methylthiobenzamide (**31**, < 5% yield with *Cp*VBPO). Notably, these substrates have comparable reactivity profiles in multiple chemical-based oxidative dimerization methods,^54−56^ suggesting that this reactivity difference is related to enzyme binding. Moreover, when the reaction was performed on **30** to produce **3** using *in situ* generated HOBr,^57^ only 5% yield of the desired 1,2,4-thiadiazole was produced with 26% generation of the corresponding benzamide as a result of thioamide hydrolysis (Supplementary Figure S7), meaning that the enzyme plays a critical role in selective generation of the desired 1,2,4-thiadiazole. Previous studies by Littlechild et al. suggest that binding of bromide to *Cp*VBPO enables the formation of a contiguous hydrophobic surface following rearrangement of two residues (L337 and F373) within the substrate binding pocket. This hydrophobic patch bridges the dimer interface and is hypothesized to play a key role in binding organic substrates.^58,59^ Using the crystal structure recently deposited by Isupov et al., which contains both vanadate and bromide ions (PDB: 7QWI), we docked each substrate to the enzyme using the SwissDock server (Figure 5a, Supplementary Figure S8a). Note that the constituent subunits were considered as one molecule for docking simulations, as formation of the substrate binding tunnel requires the intact catalytic dimer.

Resultant docking models depict **30** approximately 4.4 Å from the vanadate ion with the thiocarbonyl group closest to the metallocofactor (Figure 5a). Such an orientation agrees with our mechanistic investigation, indicating that formation of iminobenzathiamide **II** occurs primarily through S-bromination of the starting thioamide. As expected, significant hydrophobic contacts are observed between the enzyme and the substrate; however, several hydrogen bonding interactions with residues from chain A are also predicted (Figure 5b). The substrate thioamide, for example, appears to interact with the backbone carbonyls of V332 and D335, along with the side chains of the latter and H487. Another comparatively weaker hydrogen bond may form between the thiocarbonyl and a nearby arginine (R547). The top docking pose of **31** places the analog in approximately the same location with the methyl group pointing toward open space within the active site cavity (Supplementary Figure S8a).

Given the remarkable similarity between the binding modes of **30** and **31**, we additionally attempted to dock the iminobenzathiamide intermediate (**32**) associated with **30** in the same binding site to assess whether formation of the intermediate iminobenzathiamide rather than initial substrate binding could help to rationalize differences in the reactivity. Two models emerged as potentially physiologically relevant (Figure S8b); however, the top scoring model is inconsistent with what would be expected for efficient halogenation-mediated cyclization to occur, as the sulfur and nitrogen atoms that participate in halogenation-mediated bond formation leading to 1,2,4-thiadiazoles are misaligned and would require a geometrical reconfiguration for bond formation after the halogenation event. The second model, by contrast, depicts these two atoms in perfect alignment for an S-halogenation and a subsequent N–S bond forming event. This docking pose additionally highlights the importance of the dimerization interface for reactivity, as both monomers provide significant hydrophobic contacts (Figure 5a,b, Figure S8b). Although fewer hydrogen bonding interactions are observed, this behavior may simply be a function of limited conformational flexibility afforded by the docking protocol. Intriguingly, alignments of **31** with the docking pose of **32** suggest that F373 sterically hinders binding by certain conformations of **31**, thereby limiting reactivity. More specifically, both molecules of the substrate must bind with their 2-methyl substituents pointed away from F373 to enable the formation of the iminobenzathiamide (Figure 5c). It seems reasonable to suggest that this restriction reduces the probability of forming a competent intermediate compared to that of **30**, ultimately resulting in the diminished activity reported.

We next turned to comparative docking of substrates **30** and **31** to *Ci*VCPO, in which an inverse reactivity relationship was observed, albeit at a smaller magnitude. Unfortunately, in silico simulations with *Ci*VCPO were less conclusive. Docking simulations recapitulate the general substrate binding site hypothesized previously (Supplementary Figure S9).^60^ As with *Cp*VBPO, the substrates dock in similar orientations at the entrance to a narrow tunnel housing the vanadate ion. Both substrates lie within ∼6.2 Å of the metallocofactor, but unlike docking models with *Cp*VBPO, the aromatic ring of the substrate lies closer to vanadate than the thioamide moiety. We are loathe to place significant meaning on these or other differences of position; however, as no structures with bound halide have been solved to date, and it seems possible, if not likely, that Cl^–^ binding may induce structural rearrangements within the active site, as is observed in *Cp*VBPO.

**Figure 6:**
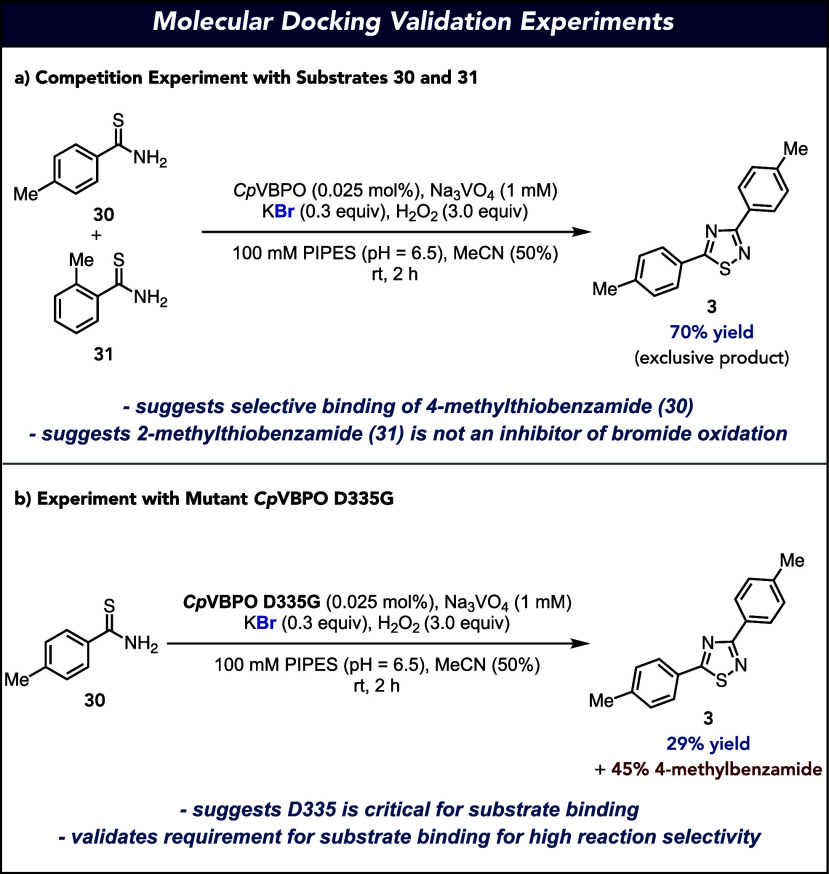
Molecular docking validation experiments. (a) Competition experiment with substrates **30** and **31**. (b) Experiment with the mutant *Cp*VBPO D335G.

One consideration for the difference in reactivity of substrates **30** and **31** is that **31** could act as an inhibitor to HOBr formation. To test this, a competition experiment was run where both substrates were introduced in a 1:1 ratio at the beginning of the reaction. Under the developed VHPO-catalyzed oxidative dimerization conditions, only the 1,2,4-thiadiazole resulting from the oxidative dimerization of 4-methylthiobenzamide (**3**) was generated in 70% yield (Figure 6a). This result suggests that *Cp*VBPO selectively binds 4-methylthiobenzamide (**30**) and that 2-methylthiobenzamide (**31**) is not acting as an inhibitor of bromide oxidation. To further confirm the importance of substrate binding for selective oxidative dimerization, we performed an experiment with the D335G variant of *Cp*VBPO, knocking out the critical D335 hydrogen bonding residue identified in our docking experiments. When the reaction was conducted using 4-methylthiobenzamide (**30**) with *Cp*VBPO D335G, a significant decrease in reaction performance was observed, producing the desired 1,2,4-thiadiazole (**3**) in 29% yield accompanied by 45% yield of 4-methylbenzamide as a result of thioamide hydrolysis (Figure 6b). This result suggests that D335 is a critical binding residue and that substrate binding is critical for selective oxidative dimerization.

**Figure 7:**
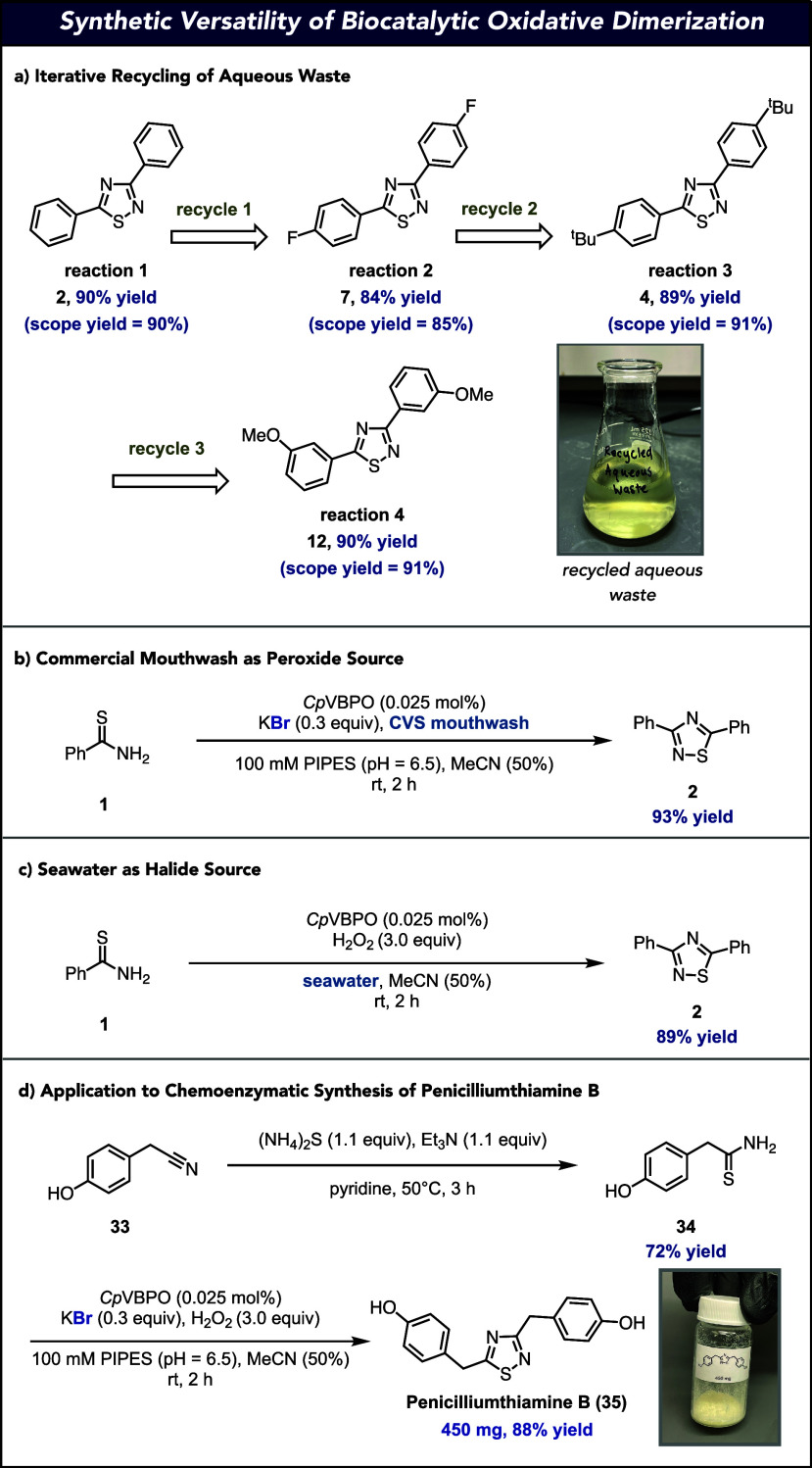
Synthetic robustness and application of VHPO-catalyzed oxidative dimerization. (a) Recycling of aqueous waste for the iterative reaction setup. (b) Replacement of H_2_O_2_ with mouthwash as the terminal oxidant. (c) Replacement of KBr with seawater as the halide source. (d) Application of VHPO-catalyzed oxidative dimerization to the chemoenzymatic synthesis of penicilliumthiamine B.

On completion of our mechanistic and modeling studies, we became interested in assessing the robustness and synthetic application of the VHPO-catalyzed oxidative dimerization protocol. First, we hypothesized that since the oxidative dimerization reaction proceeds through an enzymatic halide recycling mechanism, we should be able to use the same aqueous layer for reactions in iteration. To test this, biocatalytic oxidative dimerization was performed (vide supra) to produce 1,2,4-thiadiazole **2** on a preparative scale. On reaction completion and organic extraction, the aqueous layer from this step was directly used to produce **7** in comparable isolated yield as in the substrate scope without additional KBr required. This process was repeated two more times to produce 1,2,4-thiadiazoles **4** and **12** in yields consistent with the isolated yields in the substrate scope, highlighting that the iterative use of the aqueous layer is possible using the enzymatic halide recycling protocol (Figure 7a). To highlight the robust nature of the catalyst system, H_2_O_2_ could be replaced by a commercial mouthwash in the transformation (Figure 7b). In addition, the aqueous buffer could be replaced with seawater to achieve consistently high yields (Figure 7c). Finally, to highlight the synthetic utility of our biocatalytic oxidative dimerization, we applied it to the chemoenzymatic synthesis of the anticancer agent natural product, penicilliumthiamine B.^61^ Thioamide formation on commercially available 2-(4-hydroxyphenyl)acetonitrile (**33**) provided thioamide **34** in 72% yield. Preparative-scale VHPO-catalyzed oxidative dimerization of **34** furnished 450 mg of penicilliumthiamine B (**35**) with no overhalogenation observed (Figure 7d).

## Conclusions

In conclusion, vanadium haloperoxidases are a viable biocatalyst platform for performing intermolecular oxidative dimerization of thioamides to produce 1,2,4-thiadiazoles using an enzymatic halide recycling mechanism. This process relies on two distinct S-bromination events that enable heterocycle formation. Molecular modeling has provided structural insights into the selectivity in substrate binding for enzymes used in this study. Collectively, these studies demonstrate that enzymatic halide recycling is a powerful strategy for performing halogenation-induced intermolecular biocatalytic bond formation.

## Supporting Information Available

Experimental procedures and characterization data. Optimization data: Figure S1. Hydrogen peroxide (H_2_O_2_) loading screen. Figure S2. Cosolvent screen. Figure S3. MeCN loading screen. Figure S4. VHPO screen for 3,5-bis(2-methylphenyl)-1,2,4-thiadiazole (**16**) production. Figure S5. Potassium bromide (KBr) loading screen for production of **7**. Figure S6. Potassium bromide (KBr) loading screen for production of **16**. Procedure for oxidative dimerization of thioamides using *in situ* generated HOBr (Figure S7). Molecular modeling: Figure S8. Molecular docking models of **31** and **32** to *Cp*VBPO. Figure S9. Molecular docking of substrates to *Ci*VCPO (PDF)
