# Supplementary material for: Intermolecular 1,2,4-Thiadiazole Synthesis Enabled by Enzymatic Halide Recycling with Vanadium-Dependent Haloperoxidases
Source: J Am Chem Soc. 2025 Mar 12;147(12):10698–705. doi: 10.1021/jacs.5c01175 (PMC11951160; doi:10.1021/jacs.5c01175)

Supplementary Materials for

Intermolecular 1,2,4-Thiadiazole Synthesis Enabled by Enzymatic Halide Recycling with Vanadium-Dependent Haloperoxidases

Manik Sharma,[1,2] Cameron A. Pascoe,[2] Stacey K. Jones,[1] Sophia G. Barthel,[1]

Katherine M. Davis,*[1] & Kyle F. Biegasiewicz*[1,2]

[1] Department of Chemistry, Emory University, Atlanta, Georgia 30322, United States

[2] School of Molecular Sciences, Arizona State University, Tempe, Arizona 85281, United States

This PDF file includes:

Materials and Methods
Product Characterization
Supplementary Text and Figures
Figs. S1-S9
Spectroscopic Data
References

Table of Contents

General Experimental Information 3

General Procedures 7

Product Characterization.. 10

Additional Reaction Experimentals 33

Optimization Data 40

Molecular Modeling.. 43

References 44

Spectroscopic Data.. 45

General Experimental Information

General: Unless specified otherwise, all reagents and solvents used in this study were purchased from commercial suppliers and used as received (Sigma-Aldrich, Oakwood Chemicals, VWR, Combi-Blocks). All nonaqueous reactions were perfomed using flame-dried glassware capped with a rubber septum under a blanket of nitrogen using an inlet and outlet needle connected to a mineral oil bubbler. All aqueous reactions were carried out using the glassware indicated in the procedure without flame-drying and without an inert atmosphere. For experiments that required dried or degassed solvent, the solvent was obtained from a solvent purification system from Pure Process Technology. Deionized water (H_2O) was used in any experiments that reuire H_2O in the procedure.

Chromatography: Flash chromatography was performed on SiliaFlash® P60 (230-400 mesh, particle size 0.040-0.063 mm) using the solvent systems indictaed in each procedure. Thin-layer chromatography (TLC) was performed using Unliplate HLF 250 micron F254 precoated glass plates. A short-wave UV lamp and/or plate staining were used for TLC analysis.

Spectroscopic Characterization of Starting Materials and Products: ^{1}H- and ^{13}C-NMR were obtained on a Bruker Ascend™ (500 and 126 MHz, respectively). Chemical shifts are reported in ppm (δ) downfield from tetramethylsilane and are referenced internally to the deuterated solvent indicated. ^{1}H-NMR data is reported as follows: chemical shift [multiplicity, coupling constant (Hz), number of hydrogens]. Multiplicities are reported as follows: s (singlet), b (broad signal), d (doublet), dd (doublet of doublets), ddd (doublet of doublet of doublets), t (triplet), dt (doublet of triplets), tt (triplet of triplets), q (quartet), dq (doublet of quartets), p (pentet), m (multiplet). Infrared (IR) were acquired on a Thermo-Fisher Nicolet iS50 spectrometer and were taken neat and peaks are reported in frequency of absorption (cm^{-1}). High-resolution mass spectra were obtained using ionization techniques featuring electron impact (EI) on a mass analyzer (VG 70-VSE(A)). Analytical high-performance liquid chromatography (HPLC) was carried out using a Shimadzu LCMS-2020 System with a Kromasil EternityXT-2.5-C18 column (Dimensions: 4.6x50mm, Batch/Serial: 0000016627/A, Part No. XH2CLA05).

Protein Expression and Purification: All protein expression and purification were perfomed using previously reported procedures.[1]

<u>Sequence Information for *Cp*VBPO D335G Mutant</u>

<u>Optimized DNA Sequence for *Cp*VBPO D335G Mutant:</u>

ATGGGAATACCTGCCGACAATTTACAATCGCGTGCTAAGGCATCTTTCGACACTCGT
GTTGCTGCTGCAGAGTTGGCATTAAATCGTGGGGTCGTCCCCTCATTCGCAAATGGT
GAGGAGCTCTTATACCGGAATCCTGACCCAGACAATACTGACCCTAGTTTCATCGCA
TCATTCACTAAGGGTCTCCCACACGACGACAATGGGGCCATCATAGACCCTGACGA
CTTCTTGGCCTTCGTCCGAGCAATAAATTCTGGTGACGAGAAGGAGATAGCAGACCT
TACATTAGGTCCTGCTCGAGACCCTGAGACAGGATTGCCTATCTGGAGATCTGACTT
GGCAAATAGTCTCGAGTTAGAGGTTAGAGGATGGGAGAATTCTTCCGCCGGTCTTAC
ATTCGACCTTGAGGGGCCCGACGCACAAAGTATAGCTATGCCGCCAGCCCCTGTACT
CACTTCACCTGAGCTCGTAGCCGAGATCGCAGAGTTATACCTTATGGCTTTAGGTCG
TGAGATCGAGTTCTCTGAGTTCGACTCGCCCAAGAATGCCGAGTACATCCAATTCGC
CATCGACCAACTCAATGGGTTAGAGTGGTTCAATACACCTGCAAAGTTAGGAGACC
CACCAGCCGAGATCCGGCGTCGGCGAGGTGAGGTTACAGTTGGTAATTTATTCAGA
GGGATATTACCAGGGTCAGAGGTAGGTCCTTACTTGTCTCAATACATAATCGTCGGG
TCTAAGCAAATCGGTTCAGCCACGGTTGGTAATAAGACTCTCGTATCTCCAAATGCT
GCAGACGAGTTCGACGGTGAGATCGCTTACGGGTCTATCACTATCTCCCAAAGAGTA
CGTATCGCCACACCTGGACGGACTTCATGACAGACTTAAAGGTTTTCTTGGACGTT
CAAGACGCTGCAGACTTCCGTGGTTTCGAGTCATACGAGCCAGGAGCACGGCTTATC
CGTACAATCCGGGACTTAGCTACATGGGTACACTTCGGGGCCTTGTACGAGGCCTAC
TTGAATGCATGTTTAATCTTATTAGCAAATGGGGTCCCTTTCGACCCTAATTTACCCT
TCCAACAAGAGGACAAGTTGGACAATCAAGACGTTTTCGTCAATTTCGGGTCCGCTC
ACGTTTTATCGCTTGTTACGGAAGTCGCCACGCGTGCTCTTAAGGCCGTCCGTTACC
AAAAGTTCAATATCCACAGACGACTTCGGCCAGAGGCAACTGGTGGTCTTATCTCTG
TTAATAAGATCGCTGCACAAAAGGGTGAGTCGATCTTCCCAGAGGTTGACTTGGCTG
TAGAGGAGCTTGGTGACATATTAGAGAAGGCCGAGATATCAAATCGTAAGCAAAAT
ATAGCAGACGGAGACCCCGACCCCGACCCTTCTTTCTTATTACCCATGGCATTCGCA
GAGGGAAGTCCTTTCCACCCTAGTTACGGATCAGGTCACGCCGTTGTCGCAGGGGCT
TGTGTTACAATCTTGAAGGCCTTCTTCGACTCTGGAATCGAGATCGACCAAGTCTTC
GAGGTTGACAAGGACGAGGACAAGTTAGTCAAGTCTTCTTTCAAGGGAACACTCAC
TGTTGCAGGAGAGTTAAATAAGCTTGCAGACAATATCGCCATAGGTAGAAATATGG
CAGGTGTTCACTACTTCAGTGACCAATTCGAGTCTCTTCTTTTAGGTGAGCAAGTCGC
AATAGGTATACTTGAGGAGCAATCCTTAACTTACGGGGAGAATTTCTTCTTCAATCT
TCCAAAGTTCGACGGGACGACGATCCAAATCTAA

<u>Insertion Site Name:</u> NdeI_XhoI

<u>Vector Name:</u> pET-28a(+)

Amino Acid Sequence *Cp*VBPO D335G Mutant:

MGIPADNLQSRAKASFDTRVAAAELALNRGVVPSFANGEELLYRNPDPDNTDPSFIASFT
KGLPHDDNGAIIDPDDFLAFVRAINSGDEKEIADLTLGPARDPETGLPIWRSDLANSLELE
VRGWENSSAGLTFDLEGPDAQSIAMPPAPVLTSPELVAEIAELYLMALGREIEFSEFDSPK
NAEYIQFAIDQLNGLEWFNTPAKLGDPPAEIRRRRGEVTVGNLFRGILPGSEVGPYLSQYI
IVGSKQIGSATVGNKTLVSPNAADEFDGEIAYGSITISQRVRIATPGRDFMTDLKVFLDVQ
DAADFRGFESYEPGARLIRTIRDLATWVHFGALYEAYLNACLILLANGVPFDPNLPFQQE
DKLDNQDVFVNFGSAHVLSLVTEVATRALKAVRYQKFNIHRRLRPEATGGLISVNKIAA
QKGESIFPEVDLAVEELGDILEKAEISNRKQNIADGDPDPDPSFLLPMAFAEGSPFHPSYG
SGHAVVAGACVTILKAFFDSGIEIDQVFEVDKDEDKLVKSSFKGTLTVAGELNKLADNI
AIGRNMAGVHYFSDQFESLLLGEQVAIGILEEQSLTYGENFFFNLPKFDGTTIQI

Amino Acid Sequence with N-Terminal Tag for *Cp*VBPO D335G Mutant:

MGSSHHHHHHSSGLVPRGSHMGIPADNLQSRAKASFDTRVAAAELALNRGVVPSFAN
GEELLYRNPDPDNTDPSFIASFTKGLPHDDNGAIIDPDDFLAFVRAINSGDEKEIADLTLG
PARDPETGLPIWRSDLANSLELEVRGWENSSAGLTFDLEGPDAQSIAMPPAPVLTSPELV
AEIAELYLMALGREIEFSEFDSPKNAEYIQFAIDQLNGLEWFNTPAKLGDPPAEIRRRRGE
VTVGNLFRGILPGSEVGPYLSQYIIVGSKQIGSATVGNKTLVSPNAADEFDGEIAYGSITIS
QRVRIATPGRDFMTDLKVFLDVQDAADFRGFESYEPGARLIRTIRDLATWVHFGALYEA
YLNACLILLANGVPFDPNLPFQQEDKLDNQDVFVNFGSAHVLSLVTEVATRALKAVRY
QKFNIHRRLRPEATGGLISVNKIAAQKGESIFPEVDLAVEELGDILEKAEISNRKQNIADG
DPDPDPSFLLPMAFAEGSPFHPSYGSGHAVVAGACVTILKAFFDSGIEIDQVFEVDKDED
KLVKSSFKGTLTVAGELNKLADNIAIGRNMAGVHYFSDQFESLLLGEQVAIGILEEQSLT
YGENFFFNLPKFDGTTIQI

Molecular Modeling: Models of the substrates (**30-32**) were generated in ChemDraw, then parameterized using SwissParam,[2] which performs an energy minimization using the MMFF94 forcefield. The SwissDock web server[3] was subsequently employed to dock these substrates to the X-ray crystal structures of the bromoperoxidase from *Corallina pilulifera* (PDB accession code 7QWI) and/or chloroperoxidase from *Curvularia inaequalis* (PDB accession code 1IDQ), following removal of all small molecules, ions, and solvent. Likewise, all unnatural amino acids, e.g. BYR and HSE in 7QWI, were mutated to canonical amino acids. Given the large size of the dimer could not be accommodated by the docking server, a series of residues distant from the active site were removed from the simulation. In particular, 43 residues was truncated from the N-terminus of the first monomer and 152 residues from the C-terminus of the second monomer. To minimize spurious binding modes distant from the enzymes' active sites, we defined an orthorhombic search space centered on NE2 of His487 (7QWI) and His404 (1IDQ), respectively, and extending 10 Å in X,Y, and Z. Simulations were then run using the SwissDock 'accurate' docking type parameters, which reflect the sampling size, number of minimization steps, etc. The resultant docking models were then clustered based on their similarity and ranked according to computed CHARMM energies associated with the complex and solvent accessible surface area.[2] A representeative structure from the top ranked cluster, having the most energetically favored

interactions with each docked substrate, was then analyzed in PyMOL.[4] Note that the active site of the bromoperoxidase is formed at the interface of two monomers, which is vital to properly simulating the binding of substrates. To maintain a traditional two-body docking protocol and maintain the integrity of the binding site, these chains were relabeled as a single large molecule before submission to the SwissDock server.

General Procedures

General Analytical Procedure for Biocatalytic Oxidative Dimerization with *Cp*VBPO (General Procedure A): An enzyme aliquot of the VHPO from *Corallina pilulifera* (*Cp*VBPO, 10 µM, 100 µL) was removed from a -80 °C freezer and allowed to warm to room temperature over 5 min. After thawing, a 250 mM solution of aqueous Na_3VO_4 (4 µL) was added to the enzyme aliquot, and the resulting mixture was centrifuged for 10 seconds using a Chemglass Life Sciences MLX-108-CLS mini centrifuge and then placed at room temperature until further use. To a 1-dram vial was then added H_2O purified by an Elga purification system (185.2 µL), 500 mM pH 6.5 PIPES buffer (200 µL), and 176 mM aqueous KBr (6.82 µL, 0.3 equiv) followed by addition of 400 uL MeCN. A 40 mM solution of the starting thiobenzamide substrate in MeCN (100 µL, 1 equiv, 0.004 mmol substrate) was then added. The aliquot containing the *Cp*VBPO (0.025 mol%, 1 µM in reaction) and Na_3VO_4 (0.25 equiv) was then added to the reaction mixture followed by a 10% stock of H_2O_2 (3.95 µL, 3.0 equiv). The vial was then capped and placed on a shaker at room temperature for 1 hr. After this time, the reaction mixture was diluted with MeCN (650 µL), transferred to an Eppendorf tube, and centrifuged in a Benchmark MC-24™ Touch Centrifuge at 12,500 rpm for 5 min. After centrifugation, 650 µL of the top layer of the reaction mixture was transferred to an LCMS vial, which was then placed on an LCMS for analysis (*Note: 100 µL of a 8 mg/mL solution of 1,3,5-trimethoxybenzene was added as an internal standard for yield confirmation, where applicable)*

General Preparative Procedure for Biocatalytic Oxidative Dimerization with *Cp*VBPO (General Procedure B): Two enzyme aliquots of the VHPO from *Corallina pilulifera* (*Cp*VBPO, 10 µM, 10 mL) were removed from a -80 °C freezer and allowed to warm to room temperature over 10 min. After thawing, both enzyme aliquots were combined in a 50 mL centrifuge tube and combined with 250 mM solution of aqueous Na_3VO_4 (800 µL). This solution was then allowed to sit at room temperature for 30 minutes. To a 250 mL round bottom flask containing a magnetic stir bar was added H_2O purified by an Elga purification system (37 mL), 500 mM pH 6.5 PIPES buffer (40 mL) and 176 mM aqueous KBr (1.36 mL, 0.3 equiv). This was followed by the addition of 50 mL MeCN, a solution of the corresponding thiobenzamide substrate (1.0 equiv, 0.800 mmol) in MeCN (10 mL), and an additional 40 mL of MeCN. The contents of the centrifuge tube containing *Cp*VBPO (0.025 mol%, 1.0 µM in reaction) and Na_3VO_4 (0.25 equiv) were added to the reaction mixture followed by a 10% stock of H_2O_2 (790 µL, 3.0 equiv). The reaction was then left to stir at room temperature at 900 rpm for 2 hr. After this time, the reaction mixture was concentrated to remove the MeCN and transferred to a separatory funnel. Additional H_2O (60 mL) was added and the mixture was extracted with ethyl acetate (3 x 75 mL). The combined organic layers were then washed with brine (100 mL), dried over sodium sulfate, and concentrated under reduced pressure. The resulting crude sample was purified on a silica gel hand column to obtain the pure product.

General Analytical Procedure for Biocatalytic Oxidative Dimerization with *Cp*VBPO (Heterocycle-Containing Substrates - General Procedure C): An enzyme aliquot of the VHPO from *Corallina pilulifera* (*Cp*VBPO, 10 µM, 100 µL) was removed from a -80 °C freezer and allowed to warm to room temperature over 5 min. After thawing, a 250 mM solution of aqueous Na_3VO_4 (4 µL) was added to the enzyme aliquot and the resulting mixture was centrifuged for 10 seconds using a Chemglass Life Sciences MLX-108-CLS mini centrifuge and then placed at room temperature until further use. To a 1-dram vial was then added H_2O purified by an Elga purification system (123.9 µL), 500 mM pH 5 citrate buffer (200 µL), and 176 mM aqueous KBr (68.2 µL, 3.0 equiv) followed by the addition of 400 uL MeCN. A 40 mM solution of the thiobenzamide substrate in MeCN (100 µL, 1.0 equiv, 0.004 mmol substrate) was then added. The aliquot containing the *Cp*VBPO (0.025 mol%, 1 µM in reaction) and Na_3VO_4 (0.25 equiv) was then added to the reaction mixture followed by a 10% stock of H_2O_2 (3.95 µL, 3.0 equiv). The vial was then capped and placed on a shaker at room temperature for 1 hr. After this time, the reaction mixture was diluted with MeCN (650 µL), transferred to an Eppendorf tube, and centrifuged in a Benchmark MC-24™ Touch Centrifuge at 12,500 rpm for 5 min. After centrifugation, 650 µL of the top layer of the reaction mixture was transferred to an LCMS vial, which was then placed on an LCMS for analysis. (*Note: 100 µL of a 8 mg/mL solution of 1,3,5-trimethoxybenzene was added as an internal standard for yield confirmation, where applicable)*

General Preparative Procedure for Biocatalytic Oxidative Dimerization with *Cp*VBPO (Heterocycle-Containing Substrates - General Procedure D): Two enzyme aliquots of the VHPO from *Corallina pilulifera* (*Cp*VBPO, 10 µM, 10 mL) were removed from a -80 °C freezer and allowed to warm to room temperature over 10 min. After thawing, both the enzyme aliquots were combined in a 50 mL centrifuge tube and combined with 250 mM solution of aqueous Na_3VO_4 (800 µL). This solution was then allowed to sit at room temperature for 30 minutes. To a 250 mL round bottom flask containing a magnetic stir bar was added H_2O purified by an Elga purification system (24.8 mL), 500 mM pH 5 citrate buffer (40 mL) and 176 mM aqueous KBr (13.6 mL, 3.0 equiv). This was followed by the addition of 50 mL MeCN, a solution of the corresponding thiobenzamide substrate (1.0 equiv, 0.800 mmol) in MeCN (10 mL), and an additional 40 mL of MeCN. The contents of the centrifuge tube containing *Cp*VBPO (0.025 mol%, 1 µM in reaction) and Na_3VO_4 (0.25 equiv) were added to the reaction mixture followed by a 10% stock of H_2O_2 (790 µL, 3.0 equiv). The reaction was then left to stir at room temperature at 900 rpm for 2 hr. After this time, the reaction mixture was concentrated to remove the MeCN and transferred to a separatory funnel. Additional H_2O (60 mL) was added and the mixture was extracted with ethyl acetate (3 x 75 mL). The combined organic layers were then washed with brine (100 mL), dried over sodium sulfate, and concentrated under reduced pressure. The resulting crude sample was purified on a silica gel hand column to obtain the pure product.

General Analytical Procedure for Biocatalytic Oxidative Dimerization with *Ci*VCPO (General Procedure E): An enzyme aliquot of the VHPO from *Curvularia inaequalis* (*Ci*VCPO, 10 µM, 100 µL) was removed from a -80 °C freezer and allowed to warm to room temperature over 5 min. After thawing, a 250 mM solution of aqueous Na_3VO_4 (4 µL) was added to the enzyme aliquot, and the resulting mixture was centrifuged for 10 seconds using a Chemglass Life Sciences MLX-108-CLS mini centrifuge and then placed at room temperature until further use. To a 1-dram vial was then added H_2O purified by an Elga purification system (185.2 µL), 500 mM pH 5 citrate buffer (200 µL), and 176 mM aqueous KBr (6.82 µL, 0.3 equiv) followed by addition of 400 uL MeCN. A 40 mM solution of the thiobenzamide substrate in MeCN (100 µL, 1.0 equiv, 0.004 mmol substrate) was then added. The aliquot containing the *Ci*VCPO (0.025 mol%, 1 µM in reaction) and Na_3VO_4 (0.25 equiv) was then added to the reaction mixture followed by a 10% stock of H_2O_2 (3.95 µL, 3.0 equiv). The vial was then capped and placed on a shaker at room temperature for 1 hr. After this time, the reaction mixture was diluted with MeCN (650 µL), transferred to an Eppendorf tube, and centrifuged in a Benchmark MC-24^{TM} Touch Centrifuge at 12,500 rpm for 5 min. After centrifugation, 650 µL of the top layer of the reaction mixture was transferred to an LCMS vial, which was then placed on an LCMS for analysis. (*Note: 100 µL of a 8 mg/mL solution of 1,3,5-trimethoxybenzene was added as an internal standard for yield confirmation, where applicable)*

General Preparative Procedure for Biocatalytic Oxidative Dimerization with *Ci*VCPO (General Procedure F): Two enzyme aliquots of the VHPO from *Curvularia inaequalis* (*Ci*VCPO, 10 µM, 10 mL) were removed from a -80 °C freezer and allowed to warm to room temperature over 10 min. After thawing, both the enzyme aliquots were combined in a 50 mL centrifuge tube and combined with 250 mM solution of aqueous Na_3VO_4 (800 µL). This solution was then allowed to sit at room temperature for 30 minutes. To a 250 mL round bottom flask containing a magnetic stir bar was added H_2O purified by an Elga purification system (37 mL), 500 mM pH 5 citrate buffer (40 mL) and 176 mM aqueous KBr (1.36 mL, 0.3 equiv). This was followed by the addition of 50 mL MeCN, a solution of the corresponding thiobenzamide substrate (1.0 equiv, 0.800 mmol) in MeCN (10 mL), and an additional 40 mL of MeCN. The contents of the centrifuge tube containing *Ci*VCPO (0.025 mol%, 1 µM in reaction) and Na_3VO_4 (0.25 equiv) were added to the reaction mixture followed by a 10% stock of H_2O_2 (790 µL, 3.0 equiv). The reaction was then left to stir at room temperature at 900 rpm for 2 hr. After this time, the reaction mixture was concentrated to remove the MeCN and transferred to a separatory funnel. Additional H_2O (60 mL) was added and the mixture was extracted with ethyl acetate (3 x 75 mL). The combined organic layers were then washed with brine (100 mL), dried over sodium sulfate, and concentrated under reduced pressure. The resulting crude sample was purified on a silica gel hand column to obtain the pure product.

Product Characterization

3,5-Diphenyl-1,2,4-thiadiazole (2)

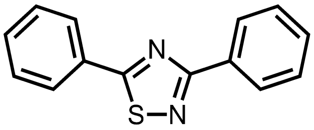

Synthesized from commercially available thiobenzamide following General Procedure B

Yield: 90%

Purification: Eluted with 5% EtOAc in Hexanes (R_f = 0.37)

^{1}H NMR (500 MHz, $CDCl_3$) δ 8.40 (dd, J = 7.5, 2.0 Hz, 2H), 8.06 (dd, J = 7.5, 2.0 Hz, 2H), 7.59 – 7.45 (m, 6H).

^{13}C NMR (126 MHz, $CDCl_3$) δ 188.2, 173.8, 132.9, 131.9, 130.8, 130.4, 129.3, 128.7, 128.4, 127.5.

HRMS: calculated for $C_{14}H_{11}N_2S$ $[M+H^+]^+$: 239.0643. Found $[M+H^+]^+$: 239.0650.

IR: (cm^{-1}) 3044.87, 1599.23, 1475.10, 1438.85, 1415.35, 1274.96, 1239.90, 760.06.

Standard Curve for Analytical Runs:

Procedure for using standard curve is as follows: 1,3,5-trimethoxybenzene (8 mg/mL solution, 100 µL) is added to 900 µL of the reaction mixture and yield is determined by LCMS analysis based on the below standard curve. LCMS conditions: 10 µL injection volume, 1.5 mL/min mobile phase rate, 10-100% B over 5.5 minutes. Mobile Phase: Solvent A- H_2O w/ 0.1% formic acid, Solvent B- MeCN w/ 0.1% formic acid.

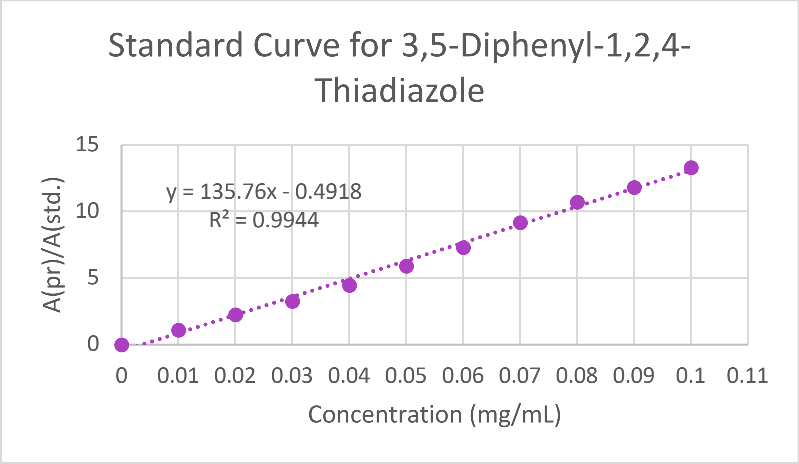

3,5-Bis(4-methylphenyl)-1,2,4-thiadiazole (3)

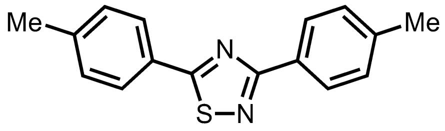

Synthesized from commercially available 4-methylthiobenzamide following General Procedure B

Yield: 81%

Purification: Eluted with 5% EtOAc in Hexanes (R_f = 0.43)

^{1}H NMR (500 MHz, $CDCl_3$) δ 8.28 (d, J = 8.0 Hz, 2H), 7.94 (d, J = 8.0 Hz, 2H), 7.32 (d, J = 8.0, 2H), 7.30 (d, J = 8.0, 2H), 2.44 (s, 3H), 2.43 (s, 3H).

^{13}C NMR (126 MHz, $CDCl_3$) δ 188.0, 173.8, 142.5, 140.5, 130.4, 129.9, 129.4, 128.3, 128.2, 127.4, 21.7, 21.5.

HRMS: calculated for $C_{16}H_{15}N_2S$ $[M+H^+]^+$: 267.0956. Found $[M+H^+]^+$: 267.0963.

IR: (cm^{-1}) 3033.72, 2920.23, 1606.75, 1470.48, 1411.05, 1402.55, 1316.99, 1269.94, 1236.81, 813.55.

3,5-Bis(4-tertbutylphenyl)-1,2,4-thiadiazole (4)

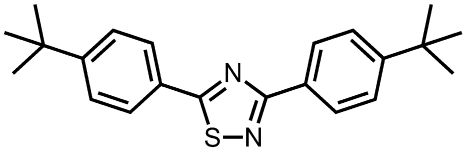

Synthesized from commercially available 4-tert-butylthiobenzamide following General Procedure B

Yield: 91%

Purification: Eluted with 5% EtOAc in Hexanes (R_f = 0.62)

^{1}H NMR (500 MHz, $CDCl_3$) δ 8.34 – 8.30 (m, 2H), 8.00 – 7.96 (m, 2H), 7.56 – 7.50 (m, 4H), 1.38 (s, 9H), 1.38 (s, 9H).

^{13}C NMR (126 MHz, $CDCl_3$) δ 188.0, 173.9, 155.7, 153.7, 130.5, 128.3, 128.2, 127.5, 126.3, 125.8, 35.5, 35.0, 21.7, 21.5.

HRMS: calculated for $C_{22}H_{17}N_2S$ $[M+H^+]^+$: 351.1895. Found $[M+H^+]^+$: 351.1900.

IR: (cm^{-1}) 3032.77, 2956.33, 2902.90, 1606.07, 1467.85, 1407.34, 1361.99, 1322.44, 1267.40, 841.05.

3,5-Bis(4-chlorophenyl)-1,2,4-thiadiazole (5)

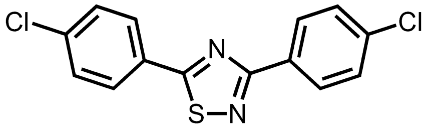

Synthesized from commercially available 4-chlorothiobenzamide following General Procedure B

Yield: 71%

Purification: Eluted with 5% EtOAc in Hexanes (R_f = 0.36)

^{1}H NMR (500 MHz, $CDCl_3$) δ 8.32 (d, J = 8.5 Hz, 2H), 7.98 (d, J = 8.5 Hz, 2H), 7.51 (d, J = 8.5 Hz, 2H), 7.47 (d, J = 8.5 Hz, 2H).

^{13}C NMR (126 MHz, $CDCl_3$) δ 187.1, 172.9, 138.2, 136.6, 131.2, 129.7, 129.7, 129.0, 129.0, 128.7.

HRMS: calculated for $C_{14}H_9N_2SCl_2$ $[M+H^+]^+$: 308.9863. Found $[M+H^+]^+$: 308.9864.

IR: (cm^{-1}) 3069.39, 2933.68, 1654.97, 1594.52, 1466.51, 1401.28, 1355.03, 828.16, 738.87.

3,5-Bis(4-bromophenyl)-1,2,4-thiadiazole (6)

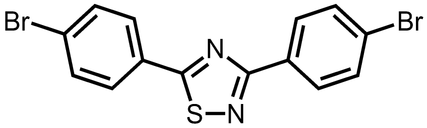

Synthesized from commercially available 4-bromothiobenzamide following General Procedure B

Yield: 52%

Purification: Eluted with 5% EtOAc in Hexanes (R_f = 0.37)

^{1}H NMR (500 MHz, $CDCl_3$) δ 8.24 (d, J = 8.5 Hz, 2H), 7.90 (d, J = 8.0 Hz, 2H), 7.67 (d, J =8.0, 8.2 Hz, 2H), 7.63 (d, J = 8.5 Hz, 2H).

^{13}C NMR (126 MHz, $CDCl_3$) δ 187.2, 172.9, 132.6, 132.0, 131.6, 129.9, 129.4, 128.9, 126.6, 125.1.

HRMS: calculated for $C_{14}H_9N_2SBr_2$ $[M+H^+]^+$: 394.8853. Found $[M+H^+]^+$: 394.8842.

IR: (cm^{-1}) 3066.65, 2921.15, 1562.62, 1490.23, 1428.06, 1300.95, 993.36, 789.16, 727.72.

3,5-Bis(4-fluorophenyl)-1,2,4-thiadiazole (7)

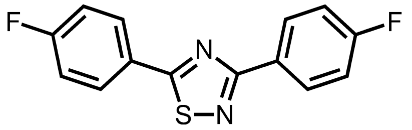

Synthesized from commercially available 4-fluorothiobenzamide following General Procedure B

Yield: 85%

Purification: Eluted with 5% EtOAc in Hexanes (R_f = 0.45)

^{1}H NMR (500 MHz, $CDCl_3$) δ 8.40 – 8.36 (m, 2H), 8.07 – 8.03 (m, 2H), 7.25 – 7.10 (m, 4H).

^{13}C NMR (126 MHz, $CDCl_3$) δ 187.0, 172.8, 164.9 (d, *J* = 252.5 Hz), 164.2 (d, *J* = 252.5 Hz), 130.4 (d, *J* = 8.6 Hz), 129.6 (d, *J* = 8.6 Hz), 129.1 (d, *J* = 3.1 Hz), 127.0 (d, *J* = 3.1 Hz), 116.6 (d, *J* = 22.3 Hz), 115.7 (d, *J* = 21.8 Hz).

HRMS: calculated for $C_{14}H_9N_2SF_2$ $[M+H^+]^+$: 275.0455. Found $[M+H^+]^+$: 275.0455.

IR: (cm^{-1}) 3049.37, 2922.02, 1596.01, 1515.79, 1470.02, 1408.36, 1315.08, 841.74, 831.86, 742.55.

3,5-Bis(4-methoxyphenyl)-1,2,4-thiadiazole (8)

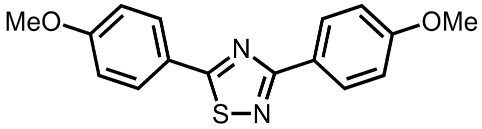

Synthesized from commercially available 4-methoxythiobenzamide following General Procedure B

Yield: 74%

Purification: Eluted with 5% EtOAc in Hexanes (R_f = 0.19)

^{1}H NMR (500 MHz, $CDCl_3$) δ 8.32 (d, J = 9.0 Hz, 2H), 7.99 (d, J = 8.5 Hz, 2H), 7.01 (d, J = 8.5 Hz, 2H), 7.01 (d, J = 9.0, Hz, 2H), 3.90 (s, 3H), 3.89 (s, 3H).

^{13}C NMR (126 MHz, $CDCl_3$) δ 187.4, 173.3, 162.6, 161.3, 129.9, 129.2, 126.0, 123.6, 114.6, 114.0, 55.5, 55.4.

HRMS: calculated for $C_{16}H_{15}N_2O_2S$ $[M+H^+]^+$: 299.0854. Found $[M+H^+]^+$: 299.0854.

IR: (cm^{-1}) 3036.42, 3001.59, 2970.16, 1606.63, 1580.60, 1474.96, 1419.51, 1305.56, 1250.13, 1182.03, 1167.10, 1052.98, 833.65.

3,5-Bis(4-hydroxyphenyl)-1,2,4-thiadiazole (9)

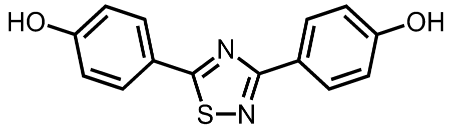

Synthesized from commercially available 4-hydroxythiobenzamide following General Procedure B

Yield: 74%

Purification: Eluted with 30% EtOAc in Hexanes (R_f = 0.18)

^{1}H NMR (500 MHz, DMSO-d_6) δ 10.36 (s, 1H), 9.99 (s, 1H), 8.11 (d, *J* = 8.5 Hz, 2H), 7.93 (d, *J* = 8.5 Hz, 2H), 6.95 (d, *J* = 8.5 Hz, 2H), 6.90 (d, *J* = 8.5 Hz, 2H).

^{13}C NMR (126 MHz, DMSO) δ 187.8, 173.2, 161.7, 160.1, 130.1, 129.8, 124.2, 121.7, 116.7, 116.1.

HRMS: calculated for $C_{14}H_{11}N_2O_2S$ $[M+H^+]^+$: 271.0541. Found $[M+H^+]^+$: 271.0540.

IR: (cm^{-1}) 3389.02, 3068.32, 2921.72, 1601.27, 1588.92, 1520.18, 1473.08, 1410.76, 1322.78, 1265.19, 1231.00, 1216.29, 1164.26, 839.33.

3,5-Bis(4-trifluoromethylphenyl)-1,2,4-thiadiazole (10)

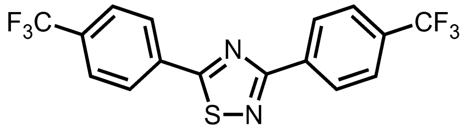

Synthesized from commercially available 4-trifluoromethylthiobenzamide following General Procedure B

Yield: 65%

Purification: Eluted with 5% EtOAc in Hexanes (R_f = 0.43)

^{1}H NMR (500 MHz, $CDCl_3$) δ 8.51 (d, *J* = 8.0 Hz, 2H), 8.18 (d, *J* = 8.0 Hz, 2H), 7.81 (d, *J* = 8.0 Hz, 2H), 7.78 (d, *J* = 8.0 Hz, 2H).

^{13}C NMR (126 MHz, $CDCl_3$) δ 187.0, 172.7, 135.6, 133.7 (q, *J* = 32.1 Hz), 133.5, 132.3 (q, *J* = 32.1 Hz), 128.7, 127.9, 126.4 (q, *J* = 3.8 Hz), 125.8 (q, *J* = 3.8 Hz), 124.0 (q, *J* = 270.4 Hz), 123.6 (q, *J* = 270.4 Hz).

HRMS: calculated for $C_{16}H_9N_2SF_6$ $[M+H^+]^+$: 375.0391. Found $[M+H^+]^+$: 375.0390.

IR: (cm^{-1}) 3058.46, 2920.36, 1597.92, 1517.74, 1472.91, 1410.35, 1317.86, 1159.69, 1133.24, 1106.02, 1065.17, 1016.63, 837.19.

3,5-Bis(3-methylphenyl)-1,2,4-thiadiazole (11)

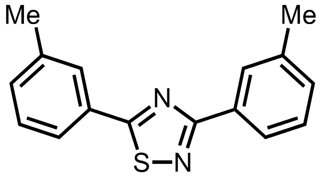

Synthesized from commercially available 3-methylthiobenzamide following General Procedure B

Yield: 85%

Purification: Eluted with 5% EtOAc in Hexanes (R_f = 0.43)

^{1}H NMR (500 MHz, $CDCl_3$) δ 8.23 (s, 1H), 8.20 (d, *J* = 7.5 Hz, 1H), 7.88 (s, 1H), 7.84 (d, *J* = 7.5 Hz, 1H), 7.40 (dd, *J* = 7.5, 7.5 Hz, 2H), 7.35 (d, *J* = 7.5 Hz, 1H), 7.30 (d, *J* = 7.5 Hz, 1H), 2.47 (s, 3H), 2.46 (s, 3H).

^{13}C NMR (126 MHz, $CDCl_3$) δ 188.3, 174.0, 139.2, 138.4, 132.9, 132.7, 131.2, 130.7, 129.2, 128.9, 128.6, 128.0, 125.6, 124.8, 21.5, 21.4.

HRMS: calculated for $C_{16}H_{15}N_2S$ $[M+H^+]^+$: 267.0956. Found $[M+H^+]^+$: 267.0963.

IR: (cm^{-1}) 3035.69, 3015.73, 2919.55, 1605.64, 1498.16, 1478.61, 1434.18, 1307.85, 1085.44, 848.11.

3,5-Bis(3-methoxyphenyl)-1,2,4-thiadiazole (12)

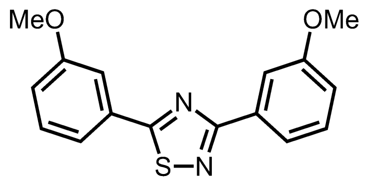

Synthesized from commercially available 3-methoxythiobenzamide following General Procedure B

Yield: 91%

Purification: Eluted with 5% EtOAc in Hexanes (R_f = 0.17)

^{1}H NMR (500 MHz, $CDCl_3$) δ 8.00 (d, J = 7.5 Hz, 1H), 7.94 (t, J = 2.0 Hz, 1H), 7.61 (t, J = 2.0 Hz, 1H), 7.59 (d, J = 7.5 Hz, 1H), 7.41 (dd, J = 8.0, 7.5 Hz, 2H), 7.08 (dd, J = 8.0, 2.0 Hz, 1H), 7.04 (dd, J = 8.0, 2.0 Hz, 1H), 3.92 (s, 3H), 3.91 (s, 3H).

^{13}C NMR (126 MHz, $CDCl_3$) δ 188.0, 173.6, 160.2, 159.9, 134.1, 131.9, 130.4, 129.8, 121.0, 120.1, 118.0, 116.8, 113.0, 112.2, 55.6, 55.5.

HRMS: calculated for $C_{16}H_{15}N_2O_2S$ $[M+H^+]^+$: 299.0854. Found $[M+H^+]^+$: 299.0857.

IR: (cm^{-1}) 3015.55, 2953.30, 2920.68, 1594.10, 1582.36, 1503.93, 1456.85, 1430.10, 1312.03, 1274.41, 1260. 43, 1046.01, 864.14.

3,5-Bis(3-chlorophenyl)-1,2,4-thiadiazole (13)

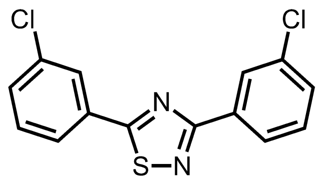

Synthesized from commercially available 3-chlorothiobenzamide following General Procedure B

Yield: 76%

Purification: Eluted with 5% EtOAc in Hexanes (R_f = 0.34)

^{1}H NMR (500 MHz, $CDCl_3$) δ 8.40 (t, *J* = 1.5 Hz, 1H), 8.27 (dt, *J* = 7.5, 1.5 Hz, 1H), 8.08 (t, *J* = 1.5 Hz, 1H), 7.91 (d, *J* = 7.5 Hz, 1H), 7.56 – 7.52 (m, 1H), 7.51 – 7.41 (m, 3H).

^{13}C NMR (126 MHz, $CDCl_3$) δ 186.9, 172.6, 135.5, 134.8, 134.2, 132.1, 132.0, 130.6, 130.5, 130.1, 128.5, 127.4, 126.4, 125.7.

HRMS: calculated for $C_{14}H_9N_2SCl_2$ $[M+H^+]^+$: 306.9863. Found $[M+H^+]^+$: 306.9858.

IR: (cm^{-1}) 3069.5, 2920.11, 1571.33, 1468.63, 1388.0, 1230.41, 1075.41, 1003.72, 840.59, 782.94, 695.42, 673.75.

3,5-Bis(3-bromophenyl)-1,2,4-thiadiazole (14)

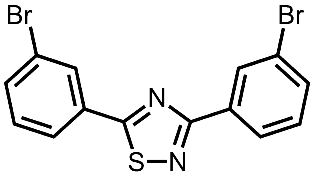

Synthesized from commercially available 3-bromothiobenzamide following General Procedure B

Yield: 75%

Purification: Eluted with 5% EtOAc in Hexanes (R_f = 0.41)

^{1}H NMR (500 MHz, $CDCl_3$) δ 8.55 (t, J = 2.0 Hz, 1H), 8.32 (d, J = 8.0 Hz, 1H), 8.23 (t, J = 2.0 Hz, 1H), 7.95 (d, J = 8.0 Hz, 1H), 7.69 (d, J = 8.0 Hz, 1H), 7.62 (d, J = 8.0 Hz, 1H), 7.41 (t, J = 8.0 Hz, 1H), 7.38 (t, J = 8.0 Hz, 1H).

^{13}C NMR (126 MHz, $CDCl_3$) δ 186.8, 172.4, 134.9, 134.5, 133.5, 132.3, 131.4, 130.8, 130.3, 130.2, 126.9, 126.2, 123.5, 122.9.

HRMS: calculated for $C_{14}H_9N_2SBr_2$ $[M+H^+]^+$: 394.8853. Found $[M+H^+]^+$: 394.8855.

IR: (cm^{-1}) 3066.65, 2921.15, 1562.62, 1490.23, 1462.13, 1428.06, 1300.95, 1212.85, 993.36, 841.82, 789.15.

3,5-Bis(3-fluorophenyl)-1,2,4-thiadiazole (15)

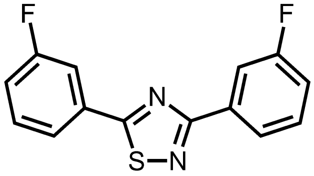

Synthesized from commercially available 3-fluorothiobenzamide following General Procedure B

Yield: 76%

Purification: Eluted with 5% EtOAc in Hexanes (R_f = 0.41)

^{1}H NMR (500 MHz, $CDCl_3$) δ 8.18 (d, *J* = 8.0 Hz, 1H), 8.08 (dt, *J* = 9.5, 2.0 Hz, 1H), 7.82 – 7.77 (m, 2H), 7.53 – 7.46 (m, 2H), 7.28 – 7.24 (m, 1H), 7.19 (td, *J* = 8.5, 2.0 Hz, 1H).

^{13}C NMR (126 MHz, $CDCl_3$) δ 187.0 (d, *J* = 3.1 Hz), 172.7 (d, *J* = 3.1 Hz), 163.1 (d, *J* = 246.6 Hz), 163.0 (d, *J* = 244.4 Hz), 134.7 (d, *J* = 8.3 Hz), 132.4 (d, *J* = 8.0 Hz), 131.1 (d, *J* = 8.3 Hz), 130.3 (d, *J* = 8.0 Hz), 124.0 (d, *J* = 3.1 Hz), 123.4 (d, *J* = 3.1 Hz), 119.0 (d, *J* = 21.3 Hz), 117.5 (d, *J* = 21.3 Hz), 115.3 (d, *J* = 23.4 Hz), 114.3 (d, *J* = 23.1 Hz).

HRMS: calculated for $C_{14}H_9N_2SF_2$ $[M+H^+]^+$: 275.0455. Found $[M+H^+]^+$: 275.0458.

IR: (cm^{-1}) 3073.21, 2922.62, 1588.70, 1481.04, 1445.66, 1295.85, 1260.76, 1153.63, 952.94, 882.28, 860.51.

3,5-Bis(2-methylphenyl)-1,2,4-thiadiazole (16)

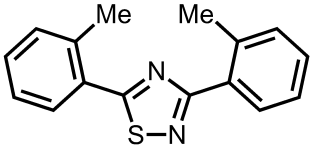

Synthesized from commercially available 2-methylthiobenzamide following General Procedure F

Yield: 72%

Purification: Eluted with 5% EtOAc in Hexanes (R_f = 0.44)

^{1}H NMR (500 MHz, $CDCl_3$) δ 8.19 (d, J = 7.5 Hz, 1H), 8.07 (d, J = 7.5 Hz, 1H), 7.46 – 7.41 (m, 1H), 7.40 – 7.31 (m, 5H), 2.76 (s, 3H), 2.71 (s, 3H).

^{13}C NMR (126 MHz, $CDCl_3$) δ 186.6, 173.8, 138.0, 137.1, 132.4, 131.8, 131.5, 131.1, 131.0, 130.2, 129.9, 129.8, 126.5, 125.9, 22.2, 22.0.

HRMS: calculated for $C_{16}H_{15}N_2S$ $[M+H^+]^+$: 267.0956. Found $[M+H^+]^+$: 267.0961.

IR: (cm^{-1}) 2957.73, 2920.60, 1492.40, 1464.40, 1443.95, 1375.95, 1308.99, 1261.51, 1086.39, 977.75, 765.56, 729.76.

3,5-Bis(2-methylphenyl)-1,2,4-thiadiazole (17)

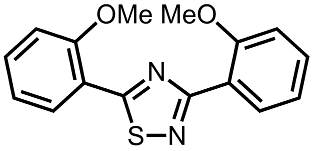

Synthesized from commercially available 2-methoxythiobenzamide following General Procedure F

Yield: 69%

Purification: Eluted with 5% EtOAc in Hexanes (R_f = 0.15)

^{1}H NMR (500 MHz, $CDCl_3$) δ 8.56 (dd, *J* = 8.0, 2.0 Hz, 1H), 8.15 (dd, *J* = 8.0, 2.0, 1H), 7.51 (t, *J* = 8.0 Hz, 1H), 7.44 (t, *J* = 8.0 Hz, 1H), 7.15 (t, *J* = 7.5 Hz, 1H), 7.11 – 7.05 (m, 3H), 4.12 (s, 3H), 3.97 (s, 3H).

^{13}C NMR (126 MHz, $CDCl_3$) δ 180.45, 169.4, 158.1, 157.6, 132.6, 132.3, 131.2, 128.6, 122.7, 121.3, 120.6, 120.1, 112.1, 111.1, 56.2, 55.9.

HRMS: calculated for $C_{16}H_{15}N_2O_2S$ $[M+H^+]^+$: 299.0854. Found $[M+H^+]^+$: 299.0856.

IR: (cm^{-1}) 3044.89, 2952.97, 2922.57, 1598.29, 1582.20, 1500.20, 1452.18, 1431.44, 1245.69, 1085.88, 1013.27, 898.96, 745.90.

3,5-Bis(2-chlorophenyl)-1,2,4-thiadiazole (18)

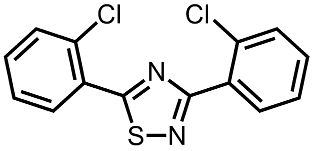

Synthesized from commercially available 2-chlorothiobenzamide following General Procedure F

Yield: 74%

Purification: Eluted with 5% EtOAc in Hexanes (R_f = 0.50)

^{1}H NMR (500 MHz, $CDCl_3$) δ 8.67 – 8.61 (m, 1H), 8.07 – 8.02 (m, 1H), 7.63 – 7.58 (m, 1H), 7.57 – 7.52 (m, 1H), 7.51 – 7.45 (m, 2H), 7.44 – 7.38 (m, 2H).

^{13}C NMR (126 MHz, $CDCl_3$) δ 183.1, 169.8, 133.9, 133.3, 132.3, 132.2, 132.1, 130.9, 130.8, 130.7, 130.5, 129.6, 127.5, 126.8.

HRMS: calculated for $C_{14}H_9N_2SCl_2$ $[M+H^+]^+$: 306.9863. Found $[M+H^+]^+$: 306.9864.

IR: (cm^{-1}) 3068.27, 2920.42, 1572.12, 1469.39, 1427.02, 1388.96, 1230.28, 1075.50, 1004.77, 919.21, 841.27, 784.34, 728.62, 674.37.

3,5-Bis(2-bromophenyl)-1,2,4-thiadiazole (19)

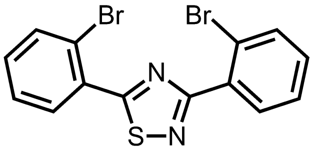

Synthesized from commercially available 2-bromothiobenzamide following General Procedure F

Yield: 73%

Purification: Eluted with 5% EtOAc in Hexanes (R_f = 0.42)

^{1}H NMR (500 MHz, $CDCl_3$) δ 8.62 (dd, *J* = 8.0, 2.0 Hz, 1H), 7.96 (dd, *J* = 8.0, 2.0 Hz, 1H), 7.78 (d, *J* = 8.0 Hz, 1H), 7.76 (d, *J* = 8.0 Hz, 1H), 7.51 (t, *J* = 7.5 Hz, 1H), 7.45 (t, *J* = 7.5 Hz, 1H), 7.39 (td, *J* = 7.5, 2.0 Hz, 1H), 7.33 (td, *J* = 7.5, 2.0 Hz, 1H).

^{13}C NMR (126 MHz, $CDCl_3$) δ 184.5, 170.7, 134.1, 134.1, 134.0, 132.3, 132.2, 131.7, 131.5, 130.9, 128.0, 127.38, 123.5, 122.2.

HRMS: calculated for $C_{14}H_9N_2SBr_2$ $[M+H^+]^+$: 394.8853. Found $[M+H^+]^+$: 394.8855.

IR: (cm^{-1}) 3060.78, 2922.05, 1582.81, 1463.97, 1432.53, 1262.24, 1198.88, 1042.69, 1027.80, 951.46, 754.34.

3,5-Bis(2-fluoroophenyl)-1,2,4-thiadiazole (20)

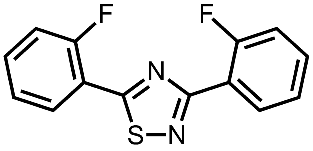

Synthesized from commercially available 2-fluorothiobenzamide following General Procedure F

Yield: 77%

Purification: Eluted with 5% EtOAc in Hexanes (R_f = 0.39)

^{1}H NMR (500 MHz, $CDCl_3$) δ 8.48 (td, *J* = 7.5, 2.0 Hz, 1H), 8.33 (td, *J* = 7.5, 2.0 Hz, 1H), 7.58 – 7.52 (m, 1H), 7.50 – 7.44 (m, 1H), 7.33 (t, *J* = 5.5 Hz, 1H), 7.33 – 7.21 (m, 3H).

^{13}C NMR (126 MHz, $CDCl_3$) δ 180.0 (d, *J* = 5.0 Hz), 168.4 (d, *J* = 5.3 Hz), 161.3 (d, *J* = 252.0 Hz), 160.9 (d, *J* = 255.0 Hz), 133.3 (d, *J* = 8.8 Hz), 131.9 (d, *J* = 1.9 Hz), 131.9 (d, *J* = 8.4 Hz), 128.9 (d, *J* = 2.4 Hz), 125.1 (d, *J* = 3.1 Hz), 124.2 (d, *J* = 3.8 Hz), 121.0 (d, *J* = 9.8 Hz), 118.8 (d, *J* = 12.0 Hz), 116.9 (d, *J* = 22.0 Hz), 116.0 (d, *J* = 20.6 Hz).

HRMS: calculated for $C_{14}H_9N_2SF_2$ $[M+H^+]^+$: 275.0455. Found $[M+H^+]^+$: 275.0456.

IR: (cm^{-1}) 2920.46, 1612.21, 1584.95, 1500.84, 1472.61, 1441.41, 1282.25, 1257.92, 1201.03, 1086.55, 989.92, 815.76, 764.02, 744.98.

3,5-Di(furan-2-yl)-1,2,4-thiadiazole (21)

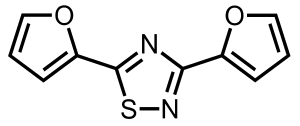

Synthesized from commercially available furan-2-thiocarboxamide following General Procedure D

Yield: 84%

Purification: Eluted with 30% EtOAc in Hexanes (R_f = 0.63)

^{1}H NMR (500 MHz, $CDCl_3$) δ 7.62 (d, *J* = 2.0 Hz, 1H), 7.60 (d, *J* = 2.0 Hz, 1H), 7.27 (d, *J* = 3.5 Hz, 1H), 7.22 (d, *J* = 3.5 Hz, 1H), 6.62 (dd, *J* = 3.5, 2.0 Hz, 1H), 6.56 (dd, *J* = 3.5, 2.0 Hz, 1H).

^{13}C NMR (126 MHz, $CDCl_3$) δ 177.1, 165.0, 148.2, 146.6, 145.7, 144.5, 112.8, 112.8, 112.7, 111.9.

HRMS: calculated for $C_{10}H_7N_2O_2S$ $[M+H^+]^+$: 219.0228. Found $[M+H^+]^+$: 219.0231.

IR: (cm^{-1}) 3139.58, 3104.53, 3054.45, 2922.22, 1588.92, 1492.32, 1408.80, 1310.59, 1224.51, 1175.20, 1032.24, 1008.20, 895.05, 761.57.

3,5-Di(thiophen-2-yl)-1,2,4-thiadiazole (22)

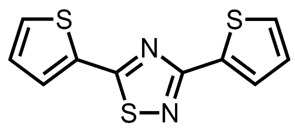

Synthesized from commercially available thiophene-2-thiocarboxamide following General Procedure D

Yield: 66%

Purification: Eluted with 10% EtOAc in Hexanes (R_f = 0.41)

^{1}H NMR (500 MHz, $CDCl_3$) δ 7.93 (dd, J = 3.5, 1.5 Hz, 1H), 7.69 (dd, J = 3.5, 1.5 Hz, 1H), 7.58 (dd, J = 5.0, 1.5 Hz, 1H), 7.46 (dd, J = 5.0, 1.5 Hz, 1H), 7.16 (dd, J = 5.0, 3.5 Hz, 1H), 7.14 (dd, J = 5.0, 3.5 Hz, 1H).

^{13}C NMR (126 MHz, $CDCl_3$) δ 180.7, 168.4, 136.2, 133.1, 130.6, 129.93, 129.3, 128.9, 128.5, 127.9.

HRMS: calculated for $C_{10}H_7N_2S_2$ $[M+H^+]^+$: 250.9771. Found $[M+H^+]^+$: 250.9777.

IR: (cm^{-1}) 3095.40, 2922.28, 1538.06, 1461.04, 1411.96, 1310.70, 1283.43, 1219.11, 1068.92, 1034.23, 868.74, 706.93.

3,5-Di(pyridin-2-yl)-1,2,4-thiadiazole (23)

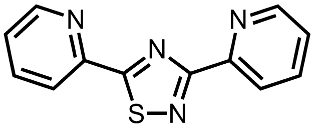

Synthesized from commercially available 2-pyridylthioamide following General Procedure D

Yield: 51%

Purification: Eluted with 3% Acetone in DCM (R_f = 0.48)

^{1}H NMR (500 MHz, $CDCl_3$) δ 8.81 (d, *J* = 4.5 Hz, 1H), 8.63 (d, *J* = 4.5 Hz, 1H), 8.39 (d, *J* = 7.5 Hz, 1H), 8.35 (d, *J* = 7.5 Hz, 1H), 7.85 (t, *J* = 7.5 Hz, 2H), 7.44 – 7.34 (m, 2H).

^{13}C NMR (126 MHz, $CDCl_3$) δ 190.3, 172.9, 150.6, 150.0, 149.9, 149.0, 137.5, 137.3, 126.4, 124.8, 123.7, 120.8.

HRMS: calculated for $C_{12}H_9N_4S$ $[M+H^+]^+$: 241.0548. Found $[M+H^+]^+$: 241.0557.

IR: (cm^{-1}) 3046.02, 2957.01, 2923.62, 1727.77, 1660.05, 1584.40, 1569.35, 1460.20, 1434.44, 1404.03, 1325.31, 1272.08, 1244.74, 1141.11, 1010.21, 782.87, 739.87, 707.89.

3,5-Di(pyridin-4-yl)-1,2,4-thiadiazole (24)

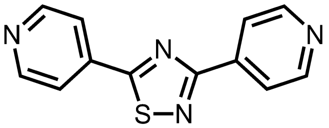

Synthesized from commercially available 4-pyridthioamide following General Procedure D

Yield: 68%

Purification: Eluted with 50-95% EtOAc in Hexanes (R_f = 0.24)

^{1}H NMR (500 MHz, $CDCl_3$) δ 8.25 – 8.18 (m, 4H), 7.65 (d, *J* = 5.0 Hz, 2H), 7.26 (d, *J* = 5.0 Hz, 2H).

^{13}C NMR (126 MHz, $CDCl_3$) δ 186.9, 171.9, 151.3, 149.9, 139.7, 136.7, 122.5, 121.0.

HRMS: calculated for $C_{12}H_9N_4S$ $[M+H^+]^+$: 241.0548. Found $[M+H^+]^+$: 241.0548.

IR: (cm^{-1}) 3031.55, 2918.52, 1599.80, 1465.55, 1408.07, 1342.88, 1250.16, 1219.05, 1127.72, 1007.20, 823.86, 744.57, 710.39.

3,5-Di(morpholine-4-yl)-1,2,4-thiadiazole (25)

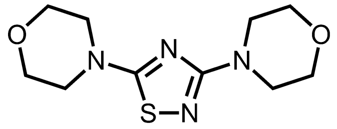

Synthesized from commercially available 4-morpholinethioamide following General Procedure D

Yield: 86%

Purification: Eluted with 30% EtOAc in Hexanes (R_f = 0.16)

^{1}H NMR (500 MHz, $CDCl_3$) δ 3.82 – 3.77 (m, 2H), 3.78 – 3.72 (m, 6H), 3.70 – 3.66 (m, 2H), 3.51 – 3.46 (m, 2H), 3.28 – 3.22 (m, 4H).

^{13}C NMR (126 MHz, $CDCl_3$) δ 188.8, 166.9, 66.6, 66.0, 65.8, 49.0, 48.6, 47.2.

HRMS: calculated for $C_{10}H_{17}N_4O_2S$ $[M+H^+]^+$: 257.1072. Found $[M+H^+]^+$: 257.1082.

IR: (cm^{-1}) 3034.13, 2962.64, 2922.38, 1551.02, 1495.20, 1443.54, 1393.97, 1369.27, 1259.00, 1109.62, 1068.36, 1002.27, 866.96.

3,5-Bis(4-methoxybenzyl)-1,2,4-thiadiazole (26)

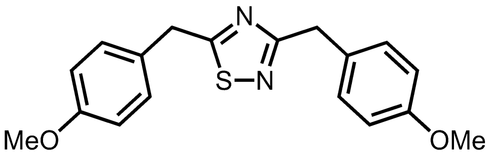

Synthesized from the previously synthesized 2-(4-methoxyphenyl)ethanethioamide following General Procedure B

Yield: 87%

Purification: Eluted with 10% EtOAc in Hexanes (R_f = 0.30)

^{1}H NMR (500 MHz, $CDCl_3$) δ 7.31 – 7.26 (m, 2H), 7.25 – 7.22 (m, 2H), 6.93 – 6.88 (m, 2H), 6.87 – 6.83 (m, 2H), 4.29 (s, 2H), 4.24 (s, 2H), 3.81 (s, 3H), 3.79 (s, 3H).

^{13}C NMR (126 MHz, $CDCl_3$) δ 193.3, 175.9, 159.2, 158.5, 130.3, 130.1, 129.3, 128.2, 114.5, 114.0, 55.3, 55.3, 38.5, 37.0.

HRMS: calculated for $C_{18}H_{19}N_2O_2S$ $[M+H^+]^+$: 327.1167. Found $[M+H^+]^+$: 327.1173.

IR: (cm^{-1}) 3010.01, 2919.88, 1607.33, 1581.55, 1509.74, 1489.57, 1453.70, 1299.75, 1243.19, 1177.28, 1024.70, 812.56.

Additional Reaction Experimental

Procedure for the Inhibition Studies for Biocatalytic Oxidative Dimerization with *Cp*VBPO
An enzyme aliquot of the VHPO from *Corallina pilulifera* (*Cp*VBPO, 10 µM, 100 µL) was removed from a -80 °C freezer and allowed to warm to room temperature over 5 min. After thawing, a 250 mM solution of aqueous Na_3VO_4 (4 µL) was added to the enzyme aliquot, and the resulting mixture was centrifuged for 10 seconds using a Chemglass Life Sciences MLX-108-CLS mini centrifuge and then placed at room temperature until further use. To a 1-dram vial was then added H_2O purified by an Elga purification system (185.2 µL), 500 mM pH 6.5 PIPES buffer (200 µL), and 176 mM aqueous KBr (6.82 µL, 0.3 equiv) followed by addition of 300 uL MeCN. A 40 mM solution of the 4-methylthiobenzamide substrate in MeCN (100 µL, 1.0 equiv, 0.004 mmol substrate) and a 40 mM solution of the 2-methylthiobenzamide substrate in MeCN (100 µL, 1.0 equiv, 0.004 mmol substrate) was then added. The aliquot containing the *Cp*VBPO (0.025 mol%, 1 µM in reaction) and Na_3VO_4 (0.25 equiv) was then added to the reaction mixture followed by a 10% stock of H_2O_2 (3.95 µL, 3.0 equiv). The vial was then capped and placed on a shaker at room temperature for 2 hr. After this time, the reaction mixture was diluted with MeCN (650 µL), transferred to an Eppendorf tube, and centrifuged in a Benchmark MC-24™ Touch Centrifuge at 12,500 rpm for 5 min. After centrifugation, 650 µL of the top layer of the reaction mixture was transferred to an LCMS vial, which was then placed on an LCMS for analysis.

Aqueous Recycling Experiments: In the recycling experiment, the reaction was run as outlined in General Procedure B with thiobenzamide. Upon reaction completion, the reaction mixture was extracted with ethyl acetate (3 x 75 mL) to remove the product generated. The aqueous solution recovered after the workup was transferred to a clean 250 mL round bottom flask that was then charged with a new substrate 4-fluorothiobenzamide (1.0 equiv, 0.800 mmol) dissolved in 100 mL of MeCN, *Cp*VBPO (0.025 mol%, 1 µM in reaction) incubated with Na_3VO_4 (0.25 equiv) and a 10% stock of H_2O_2 (790 µL, 3.0 equiv). The reaction was then left to stir at room temperature at 900 rpm for 2 hr. After the reaction completion, the reaction mixture was extracted again with ethyl acetate (3 x 75 mL) to separate the product generated. The aqueous layer recovered after workup was recycled again to run another reaction with 4-tert-butylthiobenzamide. This process involved the addition of 100 mL MeCN, 4-tert-butylthiobenzamide (1.0 equiv, 0.800 mmol), freshly incubated *Cp*VBPO (0.025 mol%, 1 µM in reaction) with Na_3VO_4 (0.25 equiv) to the round bottom flask containing recycled aqueous layer followed by addition of 10% stock of H_2O_2 (790 µL, 3.0 equiv) and was stirred for 2 hr at 900 rpm. Extractions were performed with ethyl acetate (3 x 75 mL) to remove the product formed and the aqueous layer was recycled and used to run another reaction with 3-methoxythiobenzamide. In a clean 250 mL round bottom flask, the recycled aqueous solution was combined with with 3-methoxythiobenzamide (1.0 equiv, 0.800 mmol) and MeCN (100 mL), followed by the addition of *Cp*VBPO (0.025 mol%, 1 µM in reaction) incubated with Na_3VO_4 (0.25 equiv) and 10% stock of H_2O_2 (790 µL, 3.0 equiv) and was stirred for 2 hr. Extractions were performed with ethyl acetate (3 x 75 mL) to extract the product generated. The organic extracts obtained after the reactions were separately washed with brine

(100 mL), dried over sodium sulfate, and concentrated under reduced pressure. The resulting crude samples were purified on a silica gel hand column to obtain the corresponding pure products.

Preparative Scale Biocatalytic Oxidative Dimerization Using Mouthwash as H_2O_2 Source: Two enzyme aliquots of the VHPO from *Corallina pilulifera* (*Cp*VBPO, 10 µM, 10 mL) were removed from a -80 °C freezer and allowed to warm to room temperature over 10 min. After thawing both the enzyme aliquots were transferred in a 50 mL centrifuge tube and combined with 250 mM solution of aqueous Na_3VO_4 (800 µL). This solution was then allowed to sit at room temperature for 30 minutes. To a 250 mL round bottom flask containing a magnetic stir bar was added H_2O purified by an Elga purification system (32.6 mL), 500 mM pH 6.5 PIPES buffer (40 mL) and 176 mM aqueous KBr (1.36 mL, 0.3 equiv). This was followed by the addition of 50 mL MeCN, a solution of the corresponding thiobenzamide substrate (1.0 equiv, 0.800 mmol) in MeCN (10 mL), and an additional 40 mL of MeCN. The contents of the centrifuge tube containing *Cp*VBPO (0.025 mol%, 1 µM in reaction) and Na_3VO_4 (0.25 equiv) was then added to the reaction mixture followed by a CVS brand peroxide mouthwash (1.5% H_2O_2, 5.26 mL, 3.0 equiv). The reaction was then left to stir at room temperature at 900 rpm for 2 hr. After this time, the reaction mixture was concentrated to remove the MeCN and transferred to a separatory funnel. Additional H_2O (60 mL) was added and the mixture was extracted with ethyl acetate (3 x 75 mL). The combined organic layers were then washed with brine (100 mL), dried over sodium sulfate, and concentrated under reduced pressure. The resulting crude sample was purified on a silica gel hand column to obtain the pure product.

Preparative Scale Biocatalytic Oxidative Dimerization Using Seawater as Bromide Source: Two enzyme aliquots of the VHPO from *Corallina pilulifera* (*Cp*VBPO, 10 µM, 10 mL) were removed from a -80 °C freezer and allowed to warm to room temperature over 10 min. After thawing both the enzyme aliquots were transferred in a 50 mL centrifuge tube and combined with 250 mM solution of aqueous Na_3VO_4 (800 µL). This solution was then allowed to sit at room temperature for 30 minutes. To a 250 mL round bottom flask containing a magnetic stir bar was then added seawater (38.4 mL), and 500 mM pH 6.5 PIPES buffer (40 mL). This was followed by the addition of 50 mL MeCN, a solution of the corresponding thiobenzamide substrate (1.0 equiv, 0.800 mmol) in MeCN (10 mL), and an additional 40 mL of MeCN. The contents of the centrifuge tube containing *Cp*VBPO (0.025 mol%, 1 µM in reaction) and Na_3VO_4 (0.25 equiv) were added to the reaction mixture followed by a 10% stock of H_2O_2 (790 µL, 3.0 equiv). The reaction was then left to stir at room temperature at 900 rpm for 2 hr. After this time, the reaction mixture was concentrated to remove the MeCN and transferred to a separatory funnel. Additional H_2O (60 mL) was added and the mixture was extracted with ethyl acetate (3 x 75 mL). The combined organic layers were then washed with brine (100 mL), dried over sodium sulfate, and concentrated under reduced pressure. The resulting crude sample was purified on a silica gel hand column to obtain the pure product.

Chemoenzymatic Synthesis of Penicilliumthiamine B:

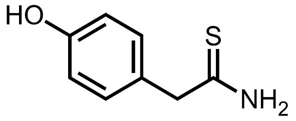

*Representative Procedure for Preparation of 2-(4-Hydroxyphenyl)ethanethioamide (**34**):* The following was adapted according to a literature procedure.[5] 4-Hydroxyphenylacetonitrile (0.800 g, 6.00 mmol, 1.0 equiv), triethylamine (0.914 mL, 6.60 mmol, 1.1 equiv) and ammonium sulfide 40% wt in water (1.13 mL, 6.60 mmol, 1.1 equiv) were dissolved in pyridine (5 mL). The reaction was stirred at 50 ºC for 3 h, at which point the reaction was cooled down, and the pyridine was removed *in vacuo*, using toluene co-evaporations (3 x 30 mL). The resulting oil was then redissolved in EtOAc (20 mL) and acidified to pH = ~7 with glacial acetic acid. This mixture was then concentrated *in vacuo* once more. The crude product was purified via flash column chromatography (silica gel, 1-10% acetone in CH_2Cl_2) to give the thioamide (0.721 g, 72% as an off-white solid.

Eluted in 10% Acetone in DCM (R_f = 0.11)

^{1}H NMR (500 MHz, DMSO) δ 9.37 (br s, 1H), 9.24 (s, 1H), 9.20 (br s, 1H), 7.14 (d, J = 8.5 Hz, 2H), 6.69 (d, J = 8.5 Hz, 2H), 3.68 (s, 2H).

^{13}C NMR (126 MHz, DMSO) δ 206.4, 156.1, 129.8, 127.5, 115.0, 50.4.

HRMS: calculated for $C_8H_{10}NOS$ $[M+H^+]^+$: 168.0483. Found $[M+H^+]^+$: 168.0486.

IR: (cm^{-1}) 3362.09, 3281.52, 3134.08, 3032.71, 2923.61, 1636.63, 1593.51, 1512.51, 1443.61, 1427.02, 1246.52, 1227.34, 987.14, 749.72.

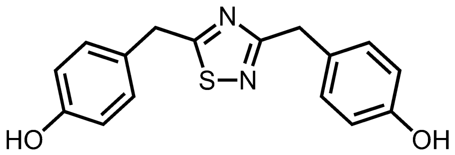

*Preparative-Scale Oxidative Dimerization Procedure for the Synthesis of Penicilliumthiamine B (**35**):* Nine enzyme aliquots of the VHPO from *Corallina pilulifera* (*Cp*VBPO, 10.0 µM, 10.0 mL) were removed from a -80 °C freezer and allowed to warm to room temperature over 10 min. After thawing the enzyme aliquots, 86 mL solution of *Corallina pilulifera* (*Cp*VBPO, 10.0 µM) was transferred to a 250 mL round bottom flask and combined with 250 mM solution of aqueous Na_3VO_4 (3.44 mL). This solution was then allowed to stir at room temperature for 30 minutes. To a 2 L round bottom flask containing a magnetic stir bar was added H_2O purified by an Elga purification system (159 mL), 500 mM pH 6.5 PIPES buffer (172 mL) and 176 mM aqueous KBr (5.85 mL, 0.3 equiv). This was followed by the addition of 300 mL MeCN, a solution of *2-(4-Hydroxyphenyl)ethanethioamide* (0.58 g, 3.44 mmol) in MeCN (100 mL), and an additional 30 mL of MeCN. The contents of the 250 mL round bottom flask containing *Cp*VBPO (0.025 mol%, 1 µM in reaction) and Na_3VO_4 (0.25 equiv) were added to the reaction mixture followed by a 10% stock of H_2O_2 (3.40 mL, 3.0 equiv). The reaction was then left to stir at room temperature at 900 rpm for 2 hr. After this time, the reaction mixture was concentrated to remove the MeCN and transferred to a separatory funnel. Additional H_2O (100 mL) was added and the mixture was extracted with ethyl acetate (3 x 100 mL). The combined organic layers were then washed with brine (100 mL), dried over sodium sulfate, and concentrated under reduced pressure. The resulting crude sample was purified as previously described.

Yield: 88%

Purification: Eluted with 50% EtOAc in Hexanes (R_f = 0.42)

^{1}H NMR (500 MHz, DMSO) δ 9.43 (s, 1H), 9.26 (s, 1H), 7.21 – 7.14 (m, 2H), 7.12 – 7.05 (m, 2H), 6.78 – 6.72 (m, 2H), 6.72 – 6.66 (m, 2H), 4.29 (s, 2H), 4.11 (s, 2H).

^{13}C NMR (126 MHz, DMSO) δ 194.0, 176.2, 157.1, 156.5, 130.7, 130.4, 127.9, 127.4, 116.1, 115.7, 38.1, 36.3.

HRMS: calculated for $C_{16}H_{15}N_2O_2S$ $[M+H^+]^+$: 299.0854 Found $[M+H^+]^+$: 299.0862.

IR: (cm^{-1}) 3139.0, 3060.89, 3018.50, 2911.39, 1611.99, 1592.89, 1514.53, 1494.47, 1434.03, 1262.98, 1229.35, 1170.16, 1101.74, 833.64.

Mechanistic Experiments

Control Experiments with *in situ* Generated Sulfoxamide Intermediate 27:

To a 1-dram vial, 296.1 µL H_2O purified by an Elga purification system, 500 mM pH 6.5 PIPES buffer (200 µL) and 400 µL MeCN was added. A 40 mM solution of the thiobenzamide substrate in MeCN (100 µL, 1.0 equiv, 0.004 mmol substrate) was then added to the reaction mixture followed by a 10% stock of H_2O_2 (3.95 µL, 3.0 equiv). The vial was then capped and placed on a shaker at room temperature for 16 hr to generate sulfoxamide. (*Note: Sulfoxamide **27** rapidly decomposed to thioamide **28** upon work-up so product formation was determined by LCMS and used directly in the below control experiments.*)

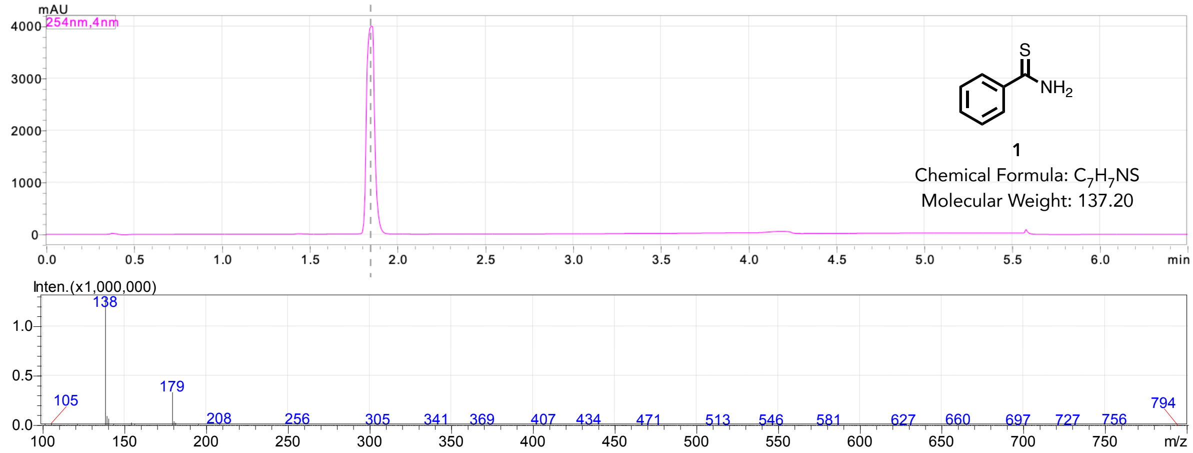

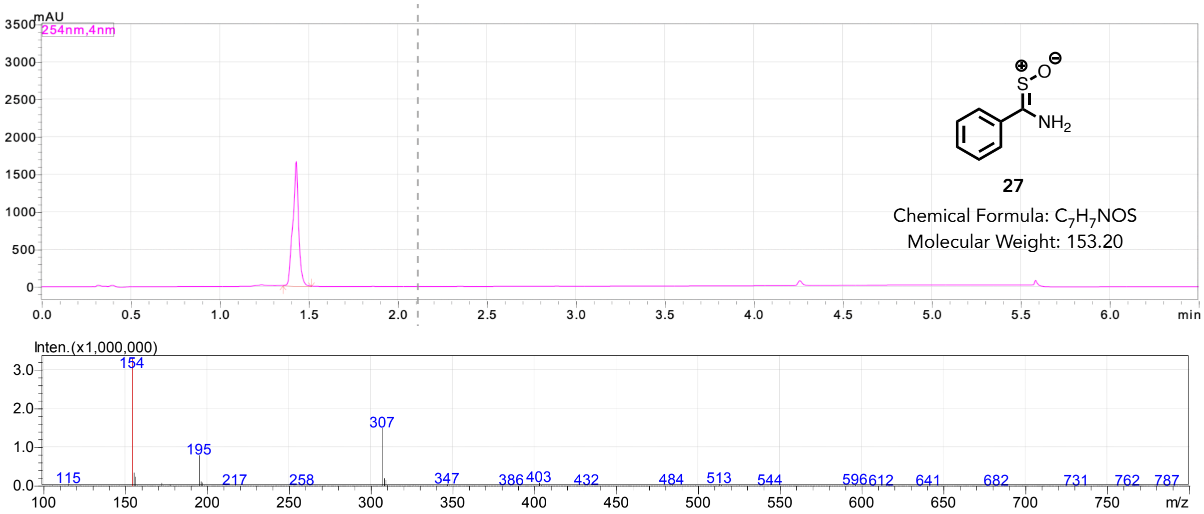

The vial containing sulfoxamide, an enzyme aliquot of the VHPO from *Corallina pilulifera* (*Cp*VBPO, 10 µM, 100 µL) incubated with a 250 mM solution of aqueous Na_3VO_4 (4 µL) was added to the reaction mixture. This was followed by the addition of addition 176 mM aqueous KBr (6.81 µL, 0.3 equiv) and 10% stock of H_2O_2 (2.90 µL, 2.0 equiv) (***Note: These reactions were run***

leaving each component out in sequence). The vial was then capped and placed on a shaker at room temperature for 1 hr. After this time, the reaction mixture was diluted with MeCN (650 µL), transferred to an Eppendorf tube, and centrifuged in a Benchmark MC-24^{TM} Touch Centrifuge at 12,500 rpm for 5 min. After centrifugation, 650 µL of the top layer of the reaction mixture was transferred to an LCMS vial, which was then placed on an LCMS for analysis. (*Note: 100 µL of a 8 mg/mL solution of 1,3,5-trimethoxybenzene was added as an internal standard for yield confirmation, where applicable)*

Preparation of N-(imino(phenyl)methyl)benzothioamide (29): The following was carried out following an adapted literature procedure.[5] Thiobenzamide (1.40 g, 10.0 mmol, 1.0 equiv) was dissolved in benzonitrile (10 mL), then had concentrated hydrochloric acid (2.42 mL, 8.0 equiv) added to the flask. The reaction was stirred at room temperature for 26 h. After the indicated time, the reaction mixture was extracted with EtOAc (3 x 50 mL). Combined organic extracts were washed with brine (100 mL), dried over Na_2SO_4, filtered and concentrated. The crude product was purified using flash column chromatography (silica gel, 30% EtOAc in hexanes) to yield the thiobenzoylbenzamidine (1.51 g, 63%) as a red solid.

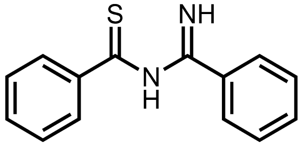

Purification: Eluted with 30% EtOAc in Hexanes (R_f = 0.45)

^{1}H NMR (500 MHz, DMSO) δ 10.93 (br s, 1H), 9.84 (br s, 1H), 8.33 – 8.28 (m, 2H), 8.13 – 8.08 (m, 2H), 7.66 (tt, *J* = 7.5, 1.5 Hz, 1H), 7.58 (app t, *J* = 7.5 Hz, 2H), 7.52 (tt, *J* = 7.5, 1.5 Hz, 1H), 7.43 (app t, *J* = 7.5 Hz, 2H).

^{13}C NMR (126 MHz, DMSO) δ 208.5, 165.9, 144.7, 134.0, 133.2, 131.7, 129.3, 128.8, 128.7, 128.3.

HRMS: calculated for $C_{14}H_{13}N_2S$ $[M+H^+]^+$: 241.0799 Found $[M+H^+]^+$: 241.0794.

IR: (cm^{-1}) 3325.12, 3053.38, 2922.49, 1592.88, 1572.21, 1437.99, 1328.51, 1212.39, 1042.72, 767.93, 683.62.

Optimization Data

All optimization reactions were perfomed using the General Analytical Procedure for Biocatalytic Oxidative Dimerization with *Cp*VBPO (General Procedure A – located in main manuscript). The only variable changed is the one indicated in the Figures below. (*Note: 100 µL of a 8 mg/mL solution of 1,3,5-trimethoxybenzene was added as an internal standard for yield confirmation, where applicable)*

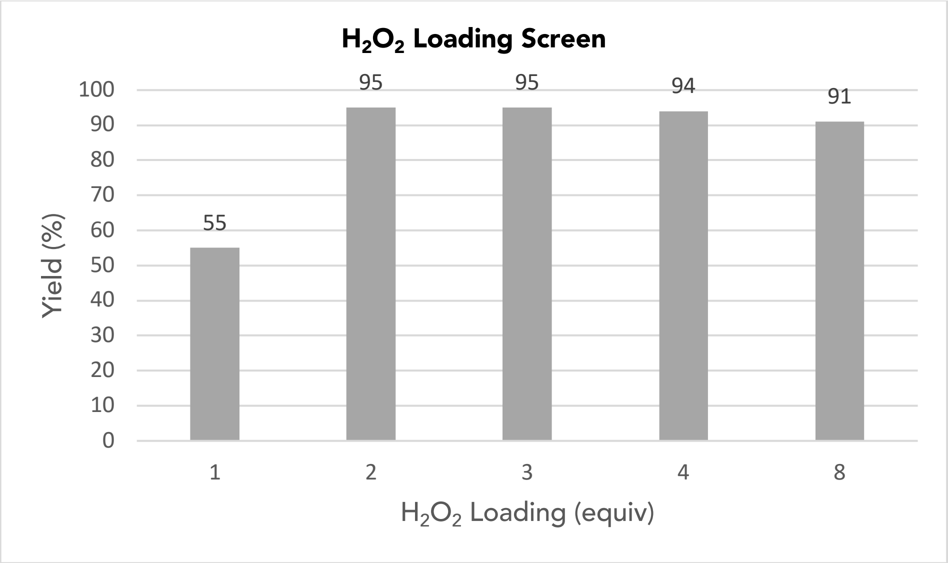

Figure S1. Hydrogen Peroxide (H_2O_2) Loading Screen

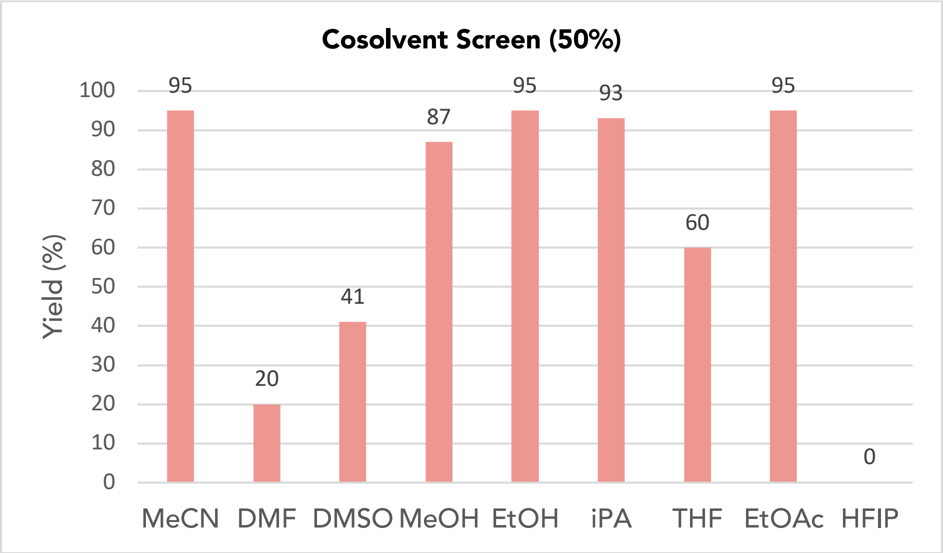

Figure S2. Cosolvent Screen

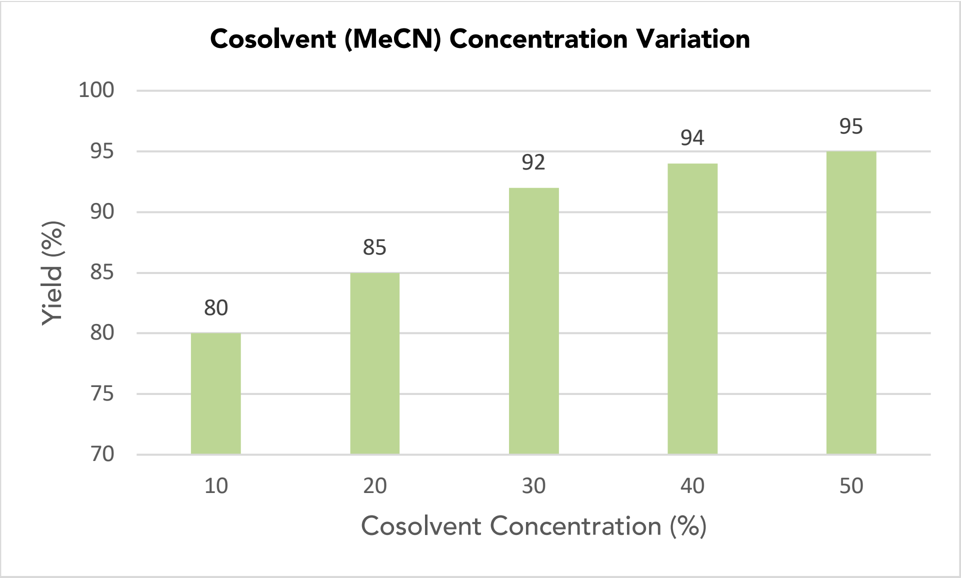

Figure S3. MeCN Loading Screen

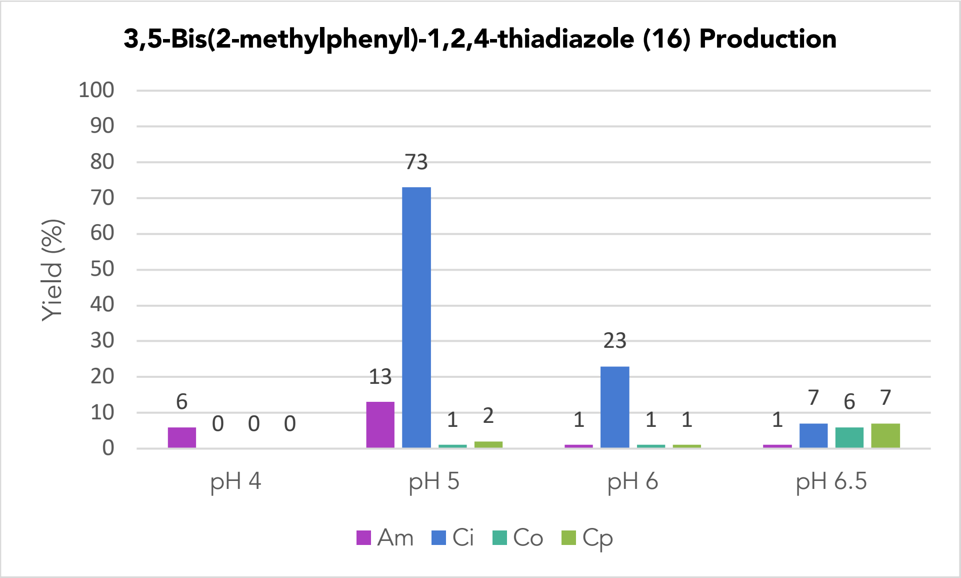

Figure S4. VHPO Screen For 3,5-Bis(2-methylphenyl)-1,2,4-thiadiazole (16) Production

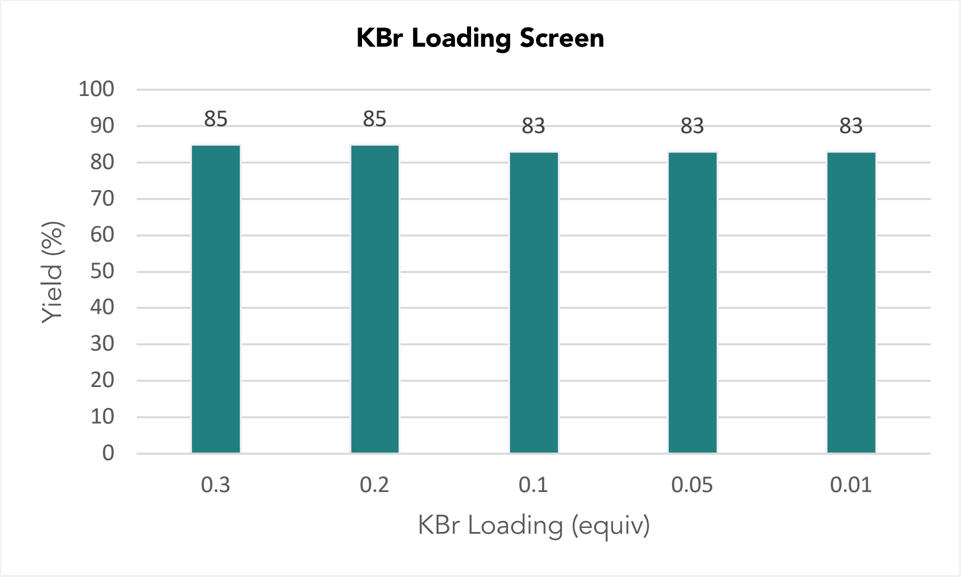

Figure S5. Potassium Bromide (KBr) Loading Screen For Production of 7

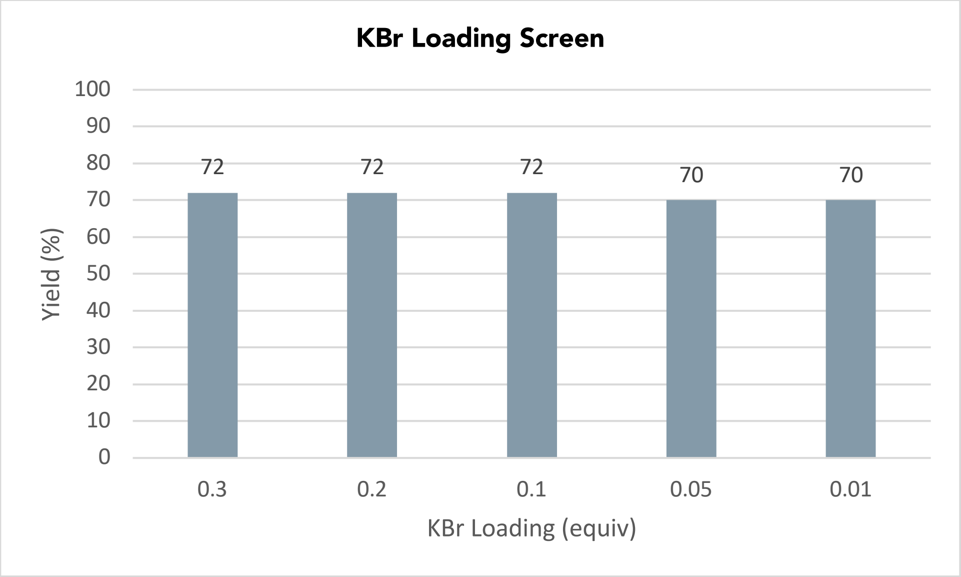

Figure S6. Potassium Bromide (KBr) Loading Screen For Production of 16

Procedure for Oxidative Dimerization of Thioamides using *in situ* Generated HOBr (General Procedure G): To a biphasic mixture of water (20 mL) and dichloromethane (20 mL), the thioamide substrate (0.16 mmol) was added, followed by potassium bromide (KBr, 0.048 mmol), ammonium metavanadate (NH_4VO_3, 0.1 mmol), and hydrogen peroxide (H_2O_2, 0.48 mmol).The

reaction mixture was allowed to stir at room temperature at 900 rpm for 2 hr. After this time, the reaction mixture was transferred to a separatory funnel. Additional H_2O (40 mL) was added, and the mixture was extracted with dichloromethane (3 x 50 mL). The combined organic layers were then washed with brine (100 mL), dried over sodium sulfate, and concentrated under reduced pressure. The resulting crude sample was purified on a silica gel hand column to obtain the pure product.

substrate	thiadiazole (yield %)	amide (yield %)
4-methylthiobenzamide	5	26
2-methylthiobenzamide	5	28

Figure S7. Oxidative Dimerization of Thioamides using *in situ* Generated HOBr

<u>**Molecular Modeling**</u>

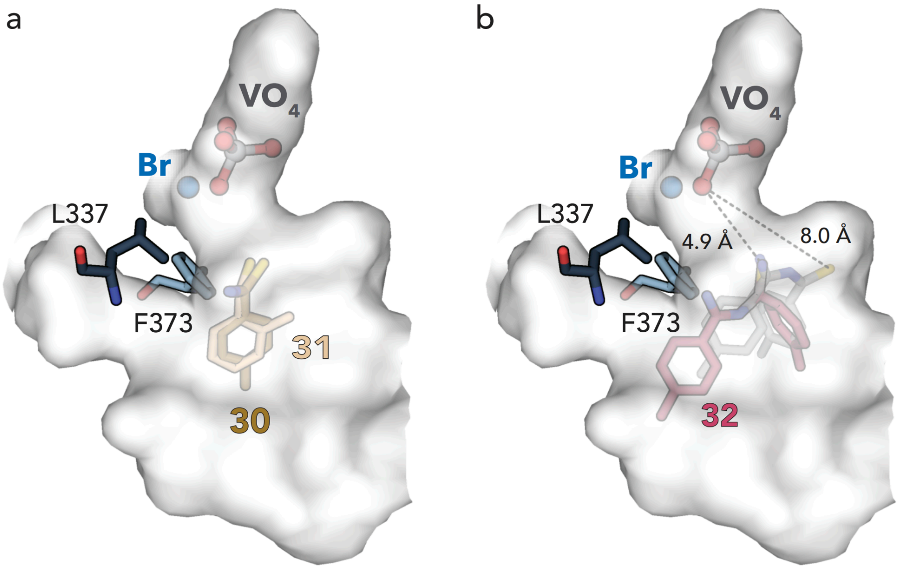

Figure S8. Molecular docking models of 31 and 32 to *Cp*VBPO. (a) Overlay of the top docking poses of **30** and **31**. (b) Overlay of the top two docking poses for the iminobenzathiamide intermediate formed by two molecules of **30**. The top scoring pose is shown in grey, while the most mechanistically relevant model is shown in maroon. Residues from the different monomeric subunits are shown in light or dark blue, respectively.

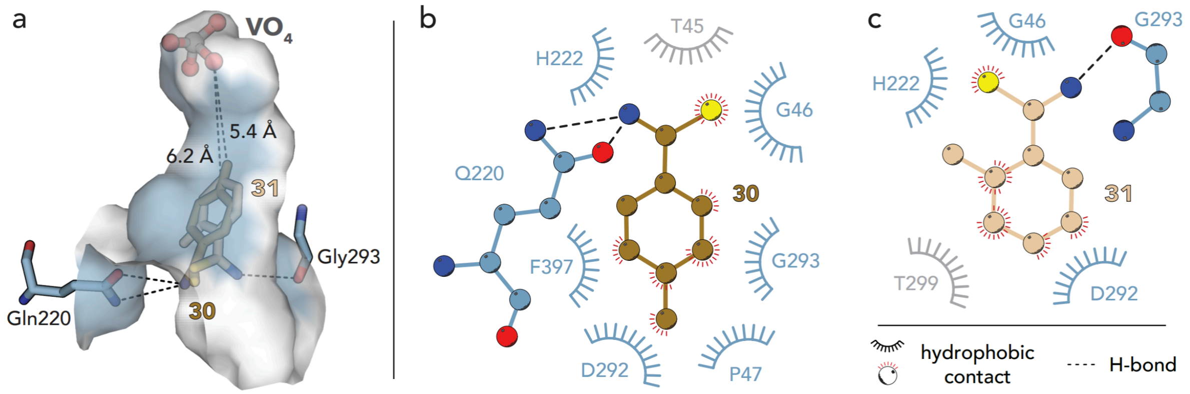

Figure S9. Molecular docking of substrates to *Ci*VCPO. The majority of interactions are hydrophobic; however, hydrogen bonds are observed with Gln220 and Gly293. (a) Overlay of **30** and **31** docking models. (b,c) 2-D interaction diagrams for (b) **30** and (c) **31** with *Ci*VCPO. In all panels, residues identified as potentially relevant for substrate binding by earlier molecular docking experiments are shown in light blue.[6]

<u>**References**</u>

(1) Wells, C. E.; Ramos, L. P. T.; Harstad, L. J.; Hessefort, L. Z.; Lee, H. J., Sharma, M.; Biegasiewicz, K. F. Decarboxylative Bromooxidation of Indoles by a Vanadium Haloperoxidase. *ACS Catal.* **2023**, *13* (7), 4622-4628.

(2) Bugnon, M.; Goullieux, M.; Röhrig, U. F.; Perez, M. A. S.; Daina, A.; Michielin, O.; Zoete, V. SwissParam 2023: A Modern Web-Based Tool for Efficient Small Molecule Parametrization. *J. Chem. Inf. Model.* **2023**, *63* (21), 6469–6475.

(3) Grosdidier, A.; Zoete, V.; Michielin, O. SwissDock, a protein-small molecule docking web service based on EADock DSS. *Nucleic Acids Res.* **2011**, *39*, W270–W277.

(4) DeLano, W. L. *CCP4 Newsl. Protein Crystallogr.* **2002**, *40*, 82–92.

(5) Zhuo, L.; Xie, S., Wang, H.; Zhu, H. Aerobic Visible-Light Induced Intermolecular S-N Bond Construction: Synthesis of 1,2,4-Thiadiazoles from Thioamides under Photosensitizer-Free Conditions. *Eur. J. Org. Chem*. **2021,** *23,* 3398-3402.

(6) Gérard, E. F.; Mokkawes, T.; Johannissen, L. O.; Warwicker, J.; Spiess, R. R.; Blanford, C. F.; Hay, S.; Heyes, D. J.; de Visser, S. P. How Is Substrate Halogenation Triggered by the Vanadium Haloperoxidase from *Curvularia inaequalis*? *ACS Catal.* **2023**, *13* (12), 8247–8261.

Spectroscopic Data

3,5-diphenyl-1,2,4-thiadiazole (2)

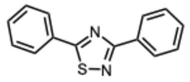

^{1}H-NMR
$CDCl_3$

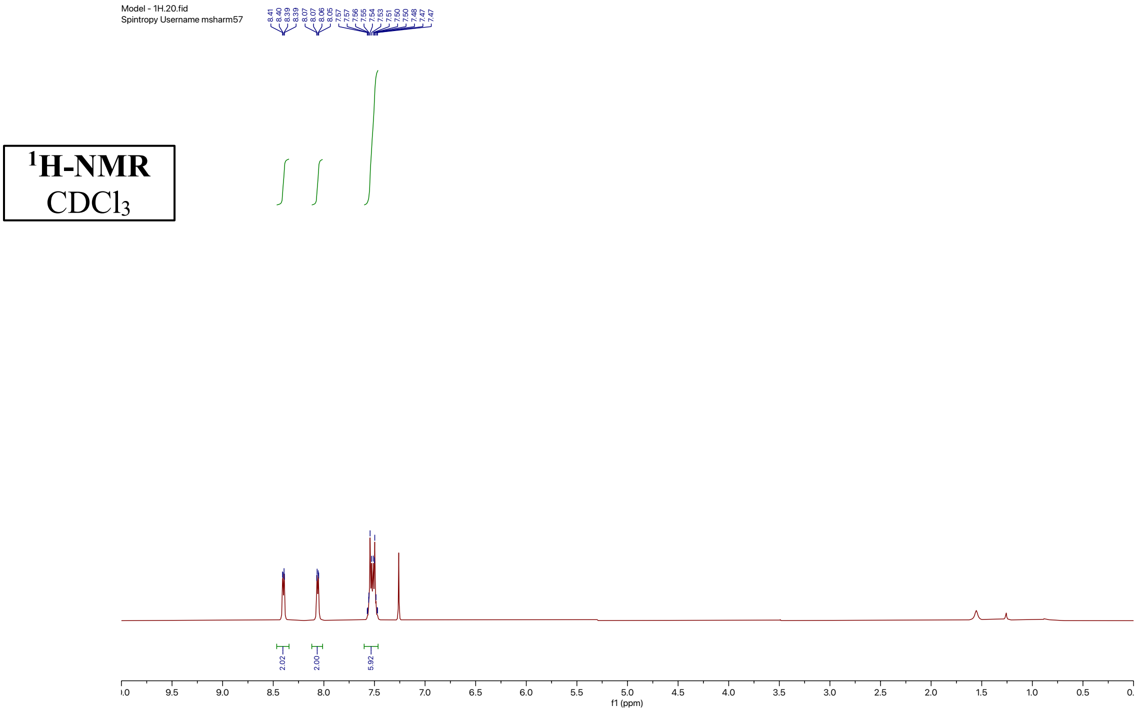

^{13}C-NMR
$CDCl_3$

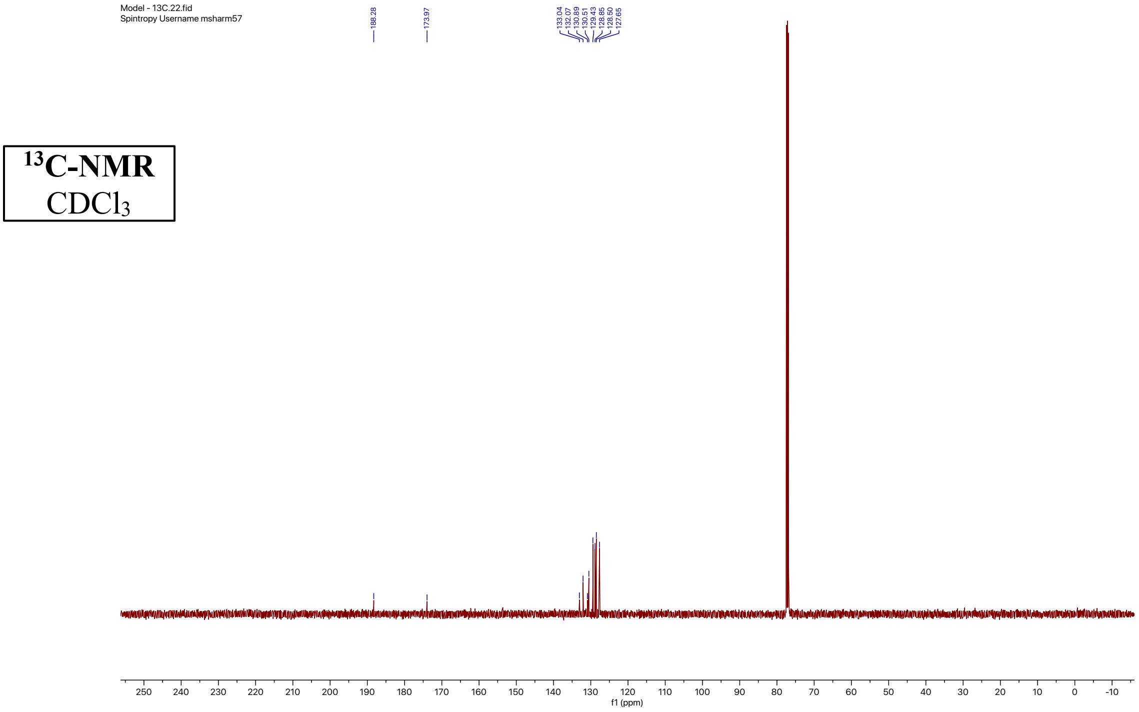

3,5-di-p-tolyl-1,2,4-thiadiazole (3)

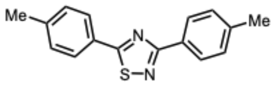

^{1}H-NMR
$CDCl_3$

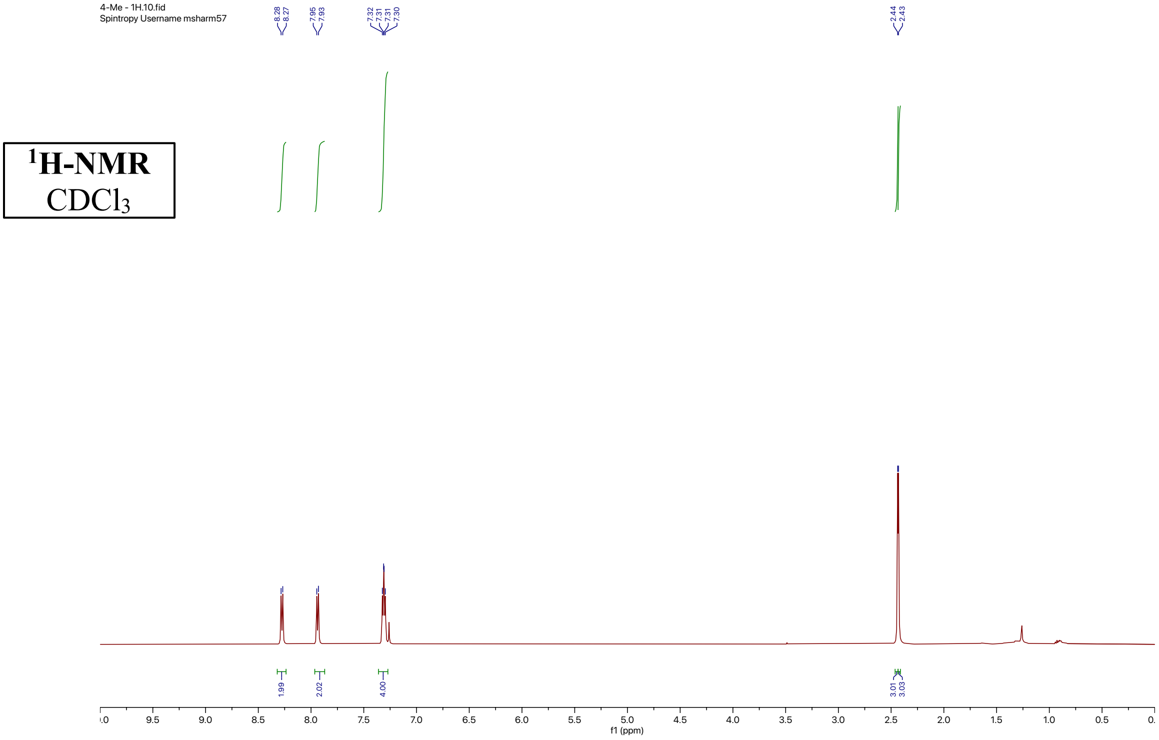

^{13}C-NMR
$CDCl_3$

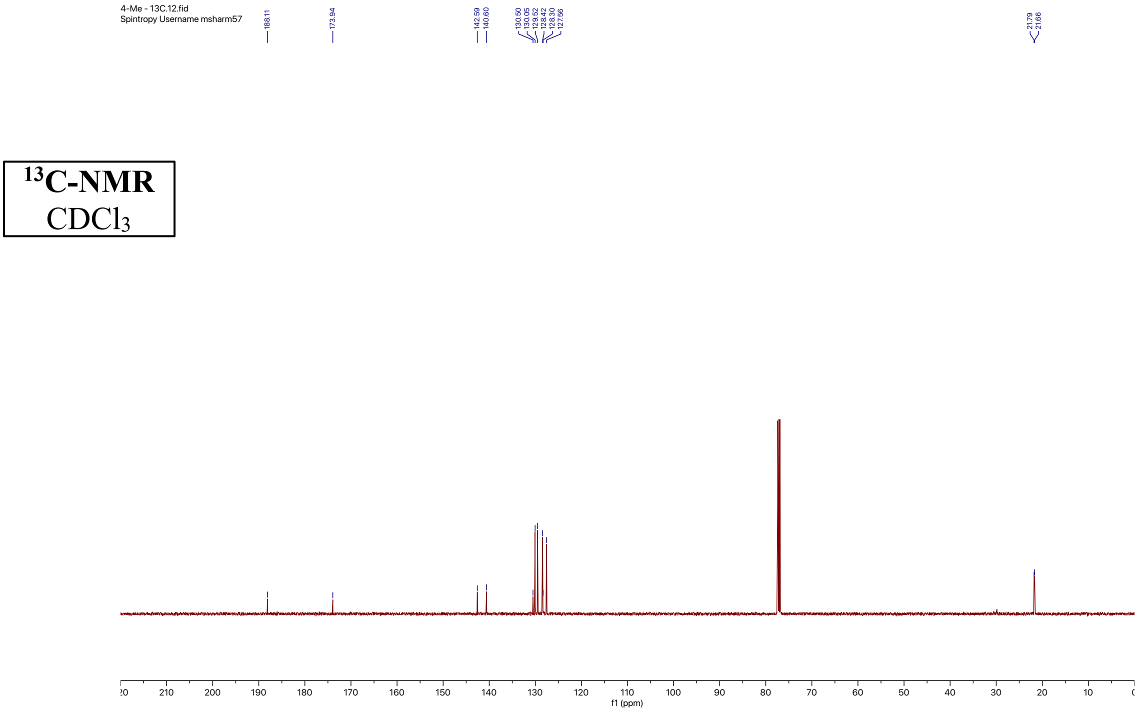

3,5-bis(4-(tert-butyl)phenyl)-1,2,4-thiadiazole (4)

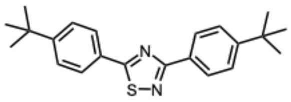

^{1}H-NMR
CDCl$_3$

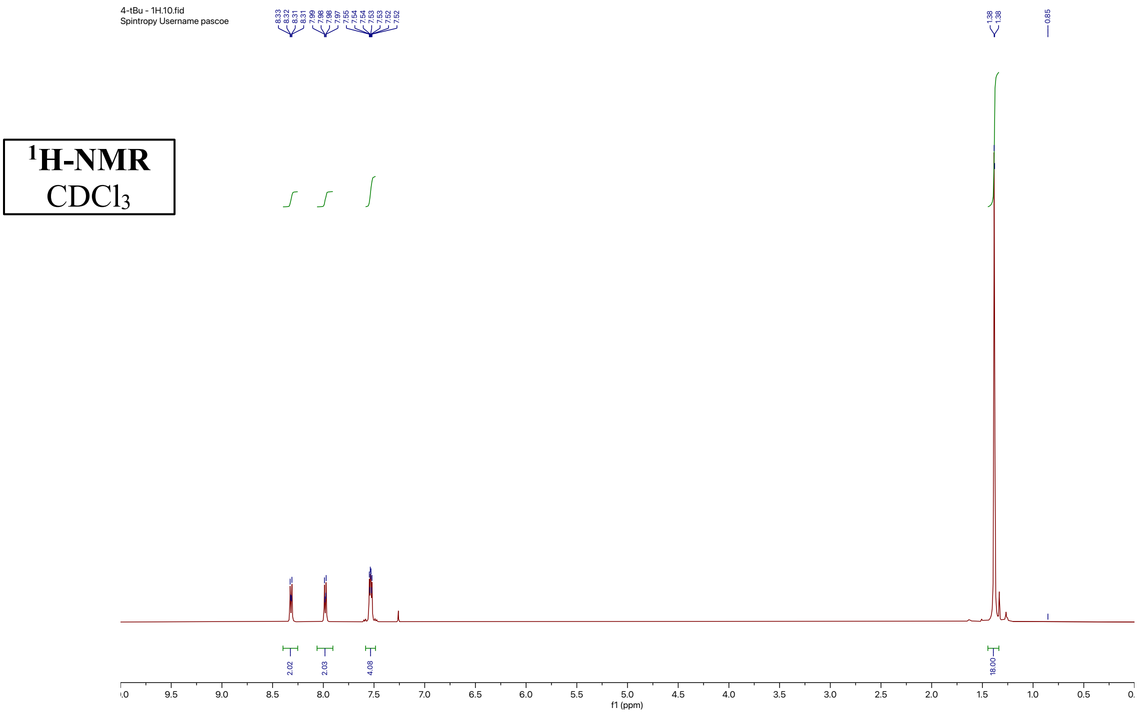

^{13}C-NMR
CDCl$_3$

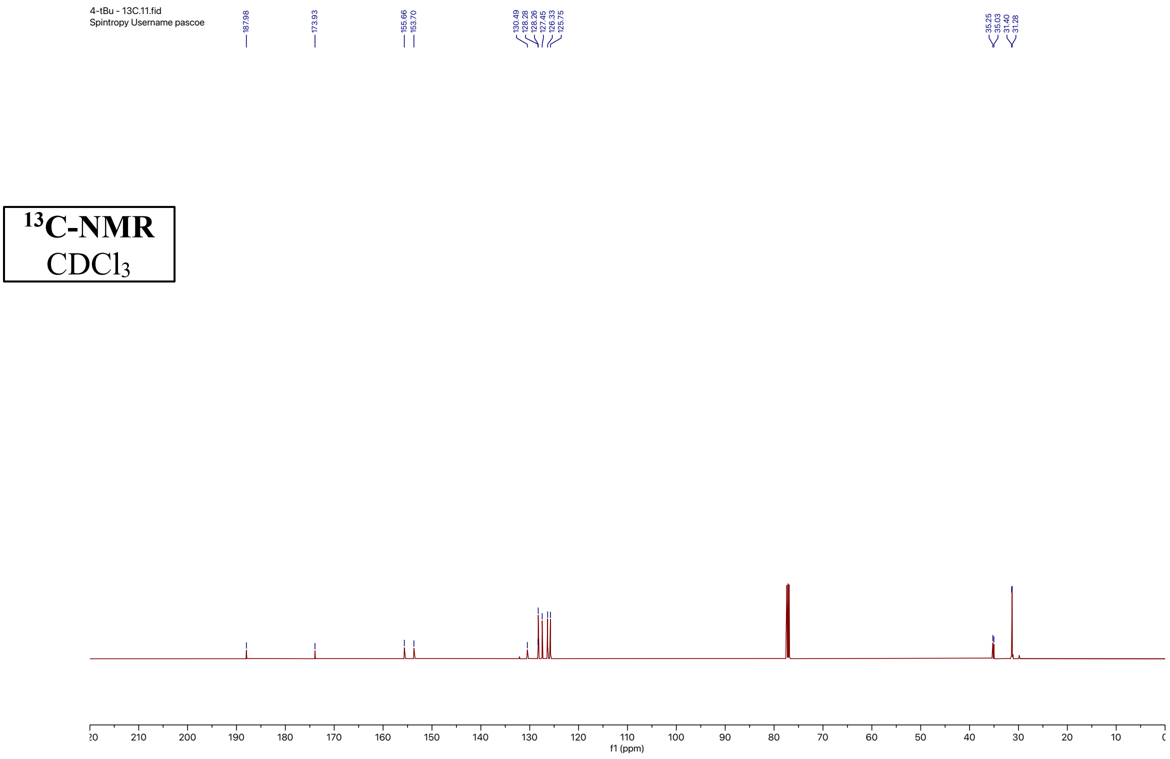

3,5-bis(4-chlorophenyl)-1,2,4-thiadiazole (5)

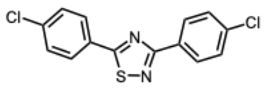

^{1}H-NMR
CDCl$_3$

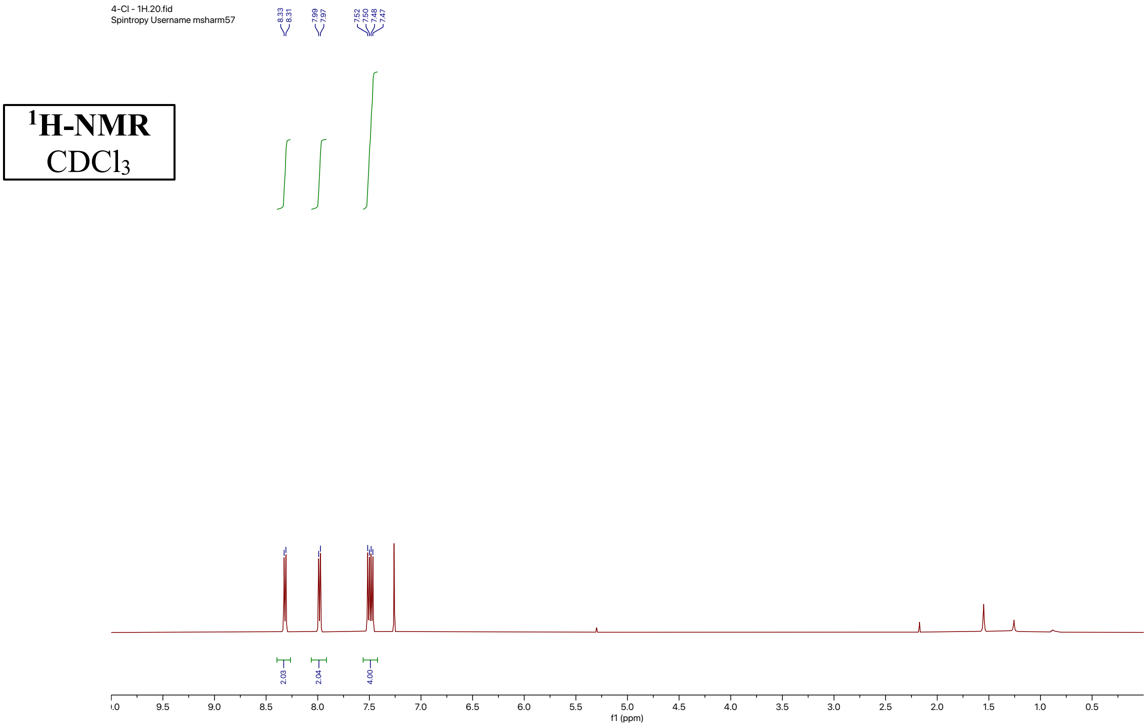

^{13}C-NMR
CDCl$_3$

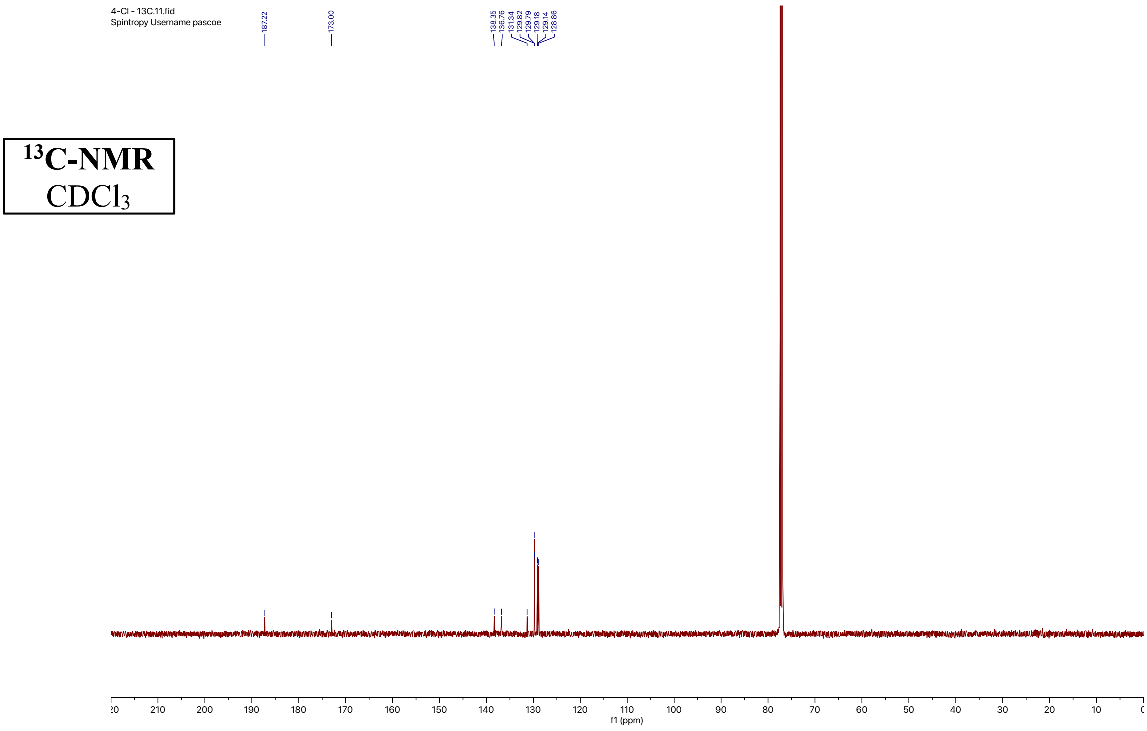

3,5-bis(4-bromophenyl)-1,2,4-thiadiazole (6)

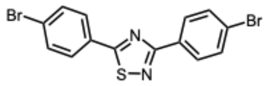

^{1}H-NMR
CDCl$_3$

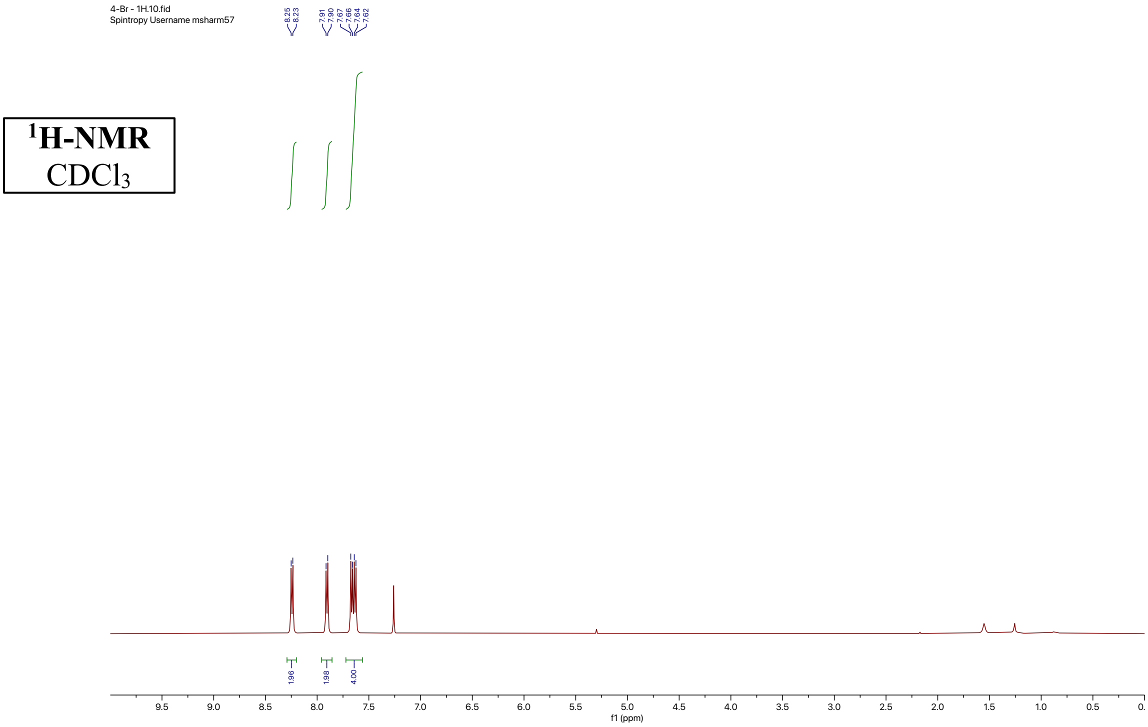

^{13}C-NMR
CDCl$_3$

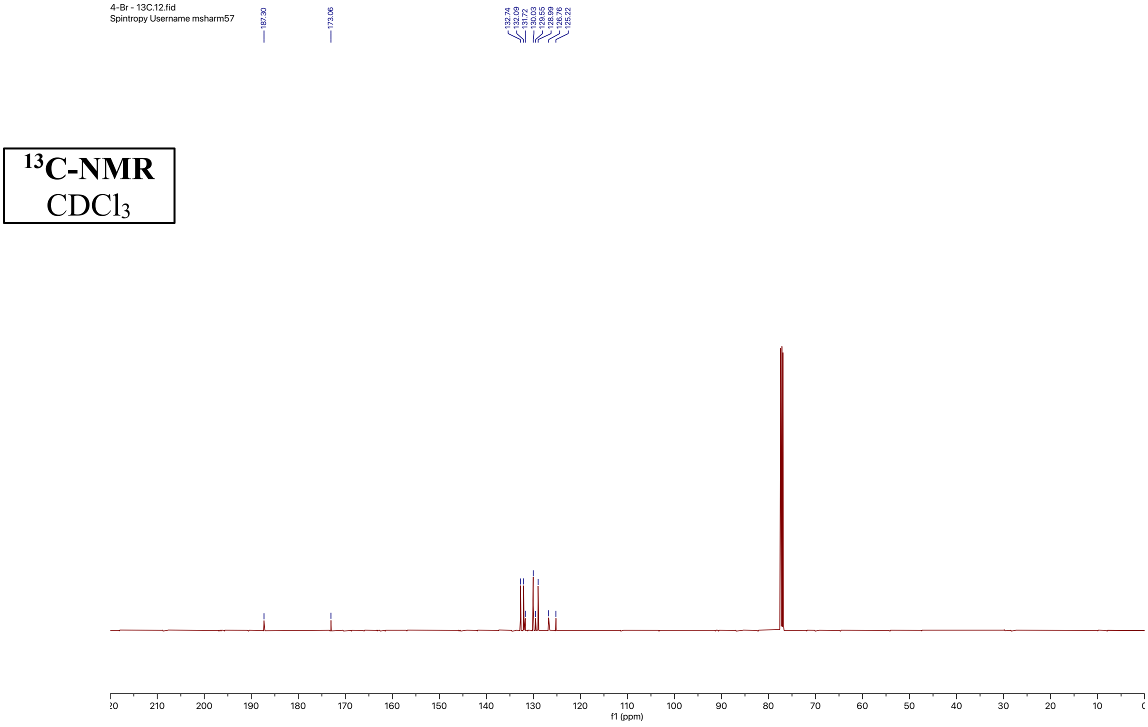

3,5-bis(4-fluorophenyl)-1,2,4-thiadiazole (7)

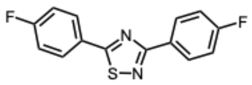

^{1}H-NMR
$CDCl_3$

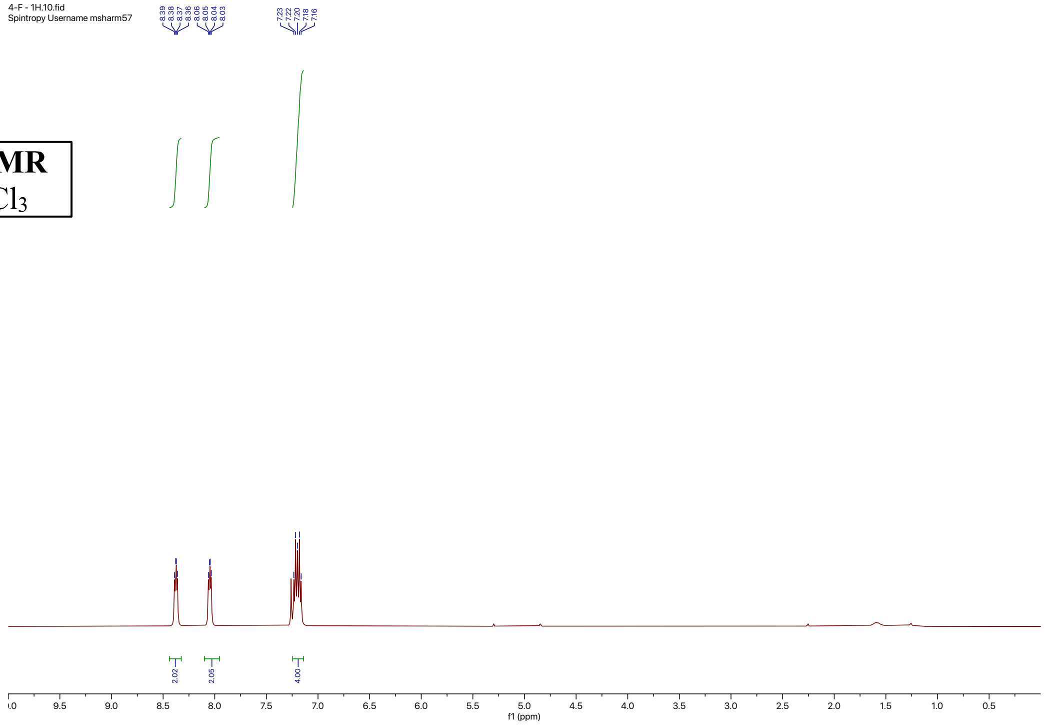

^{13}C-NMR
$CDCl_3$

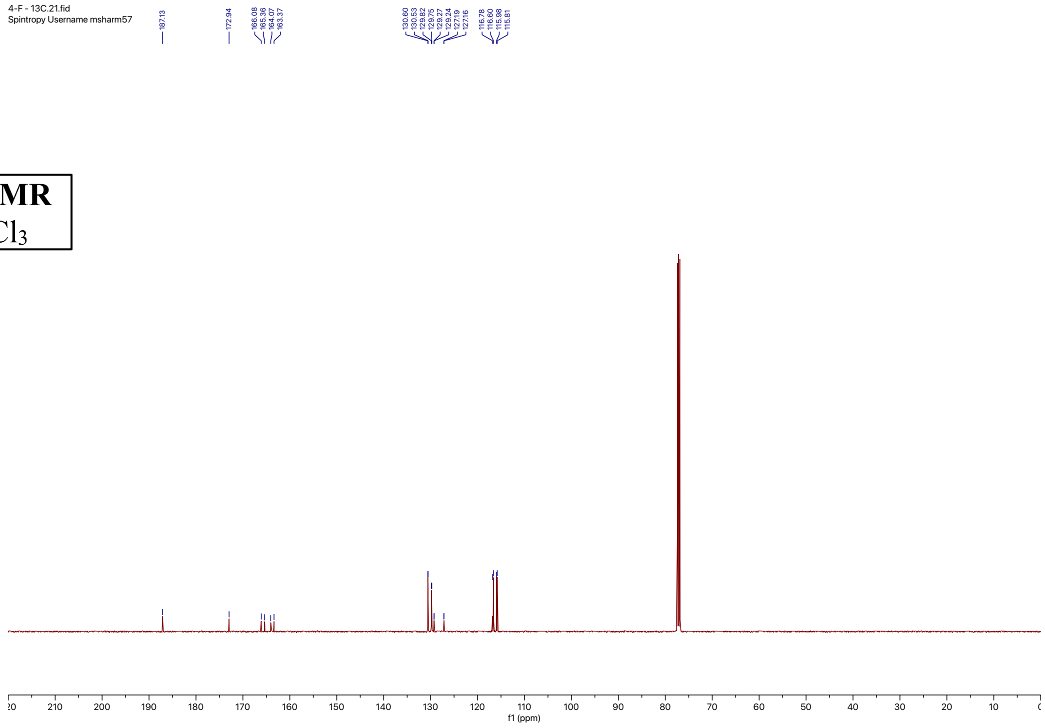

3,5-bis(4-methoxyphenyl)-1,2,4-thiadiazole (8)

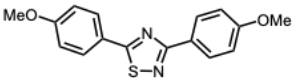

^{1}H-NMR
$CDCl_3$

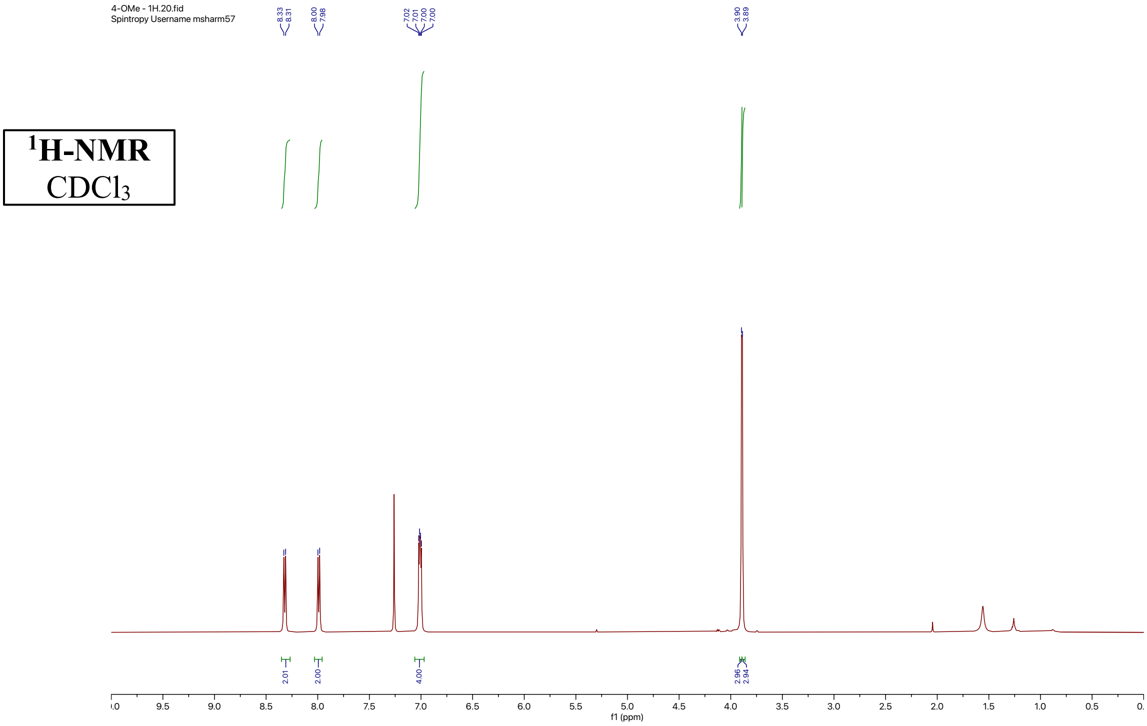

^{13}C-NMR
$CDCl_3$

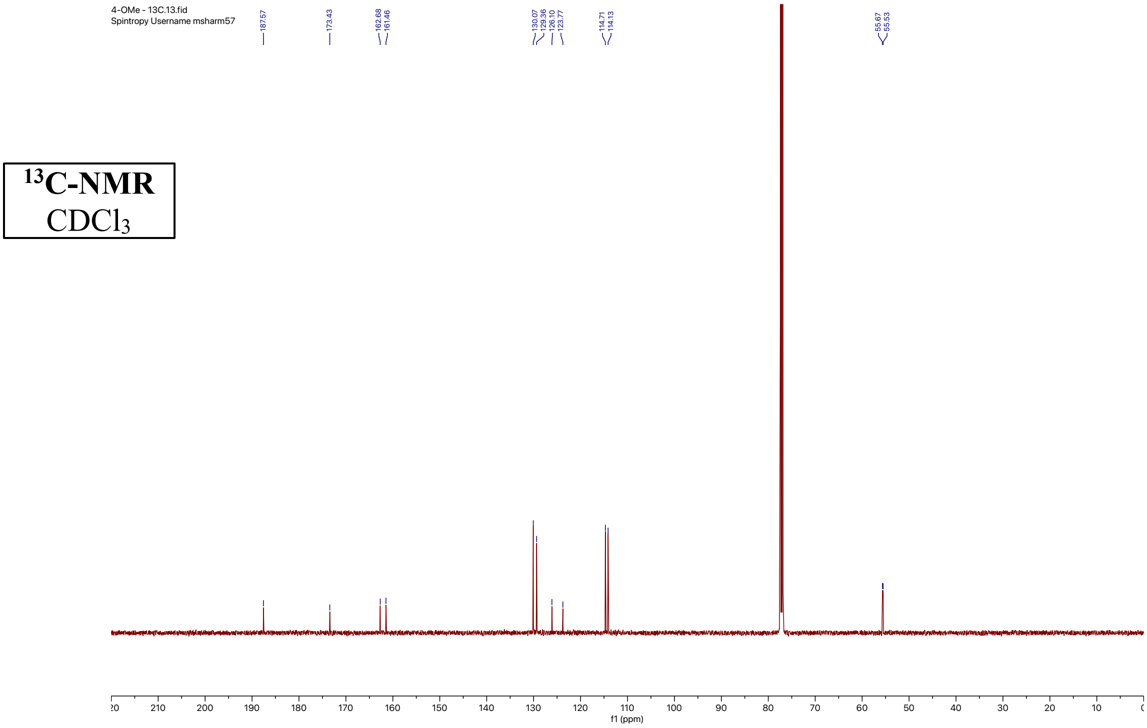

4,4'-(1,2,4-thiadiazole-3,5-diyl)diphenol (9)

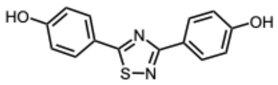

^{1}H-NMR
DMSO-d$_6$

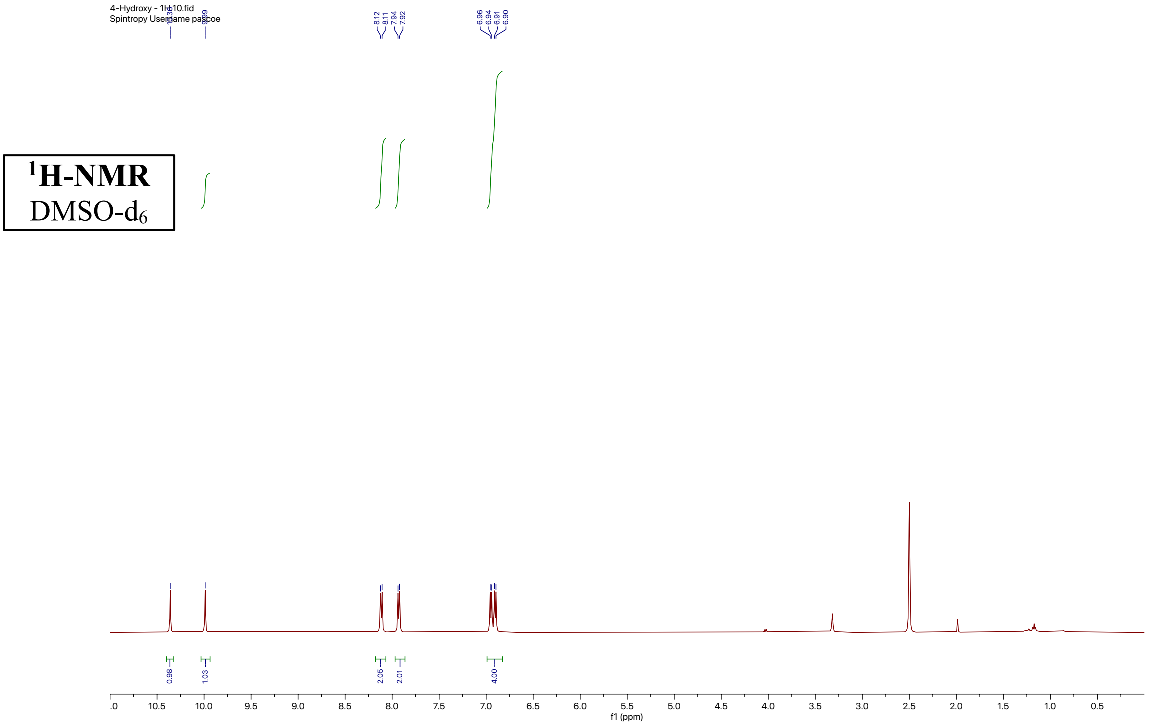

^{13}C-NMR
DMSO-d$_6$

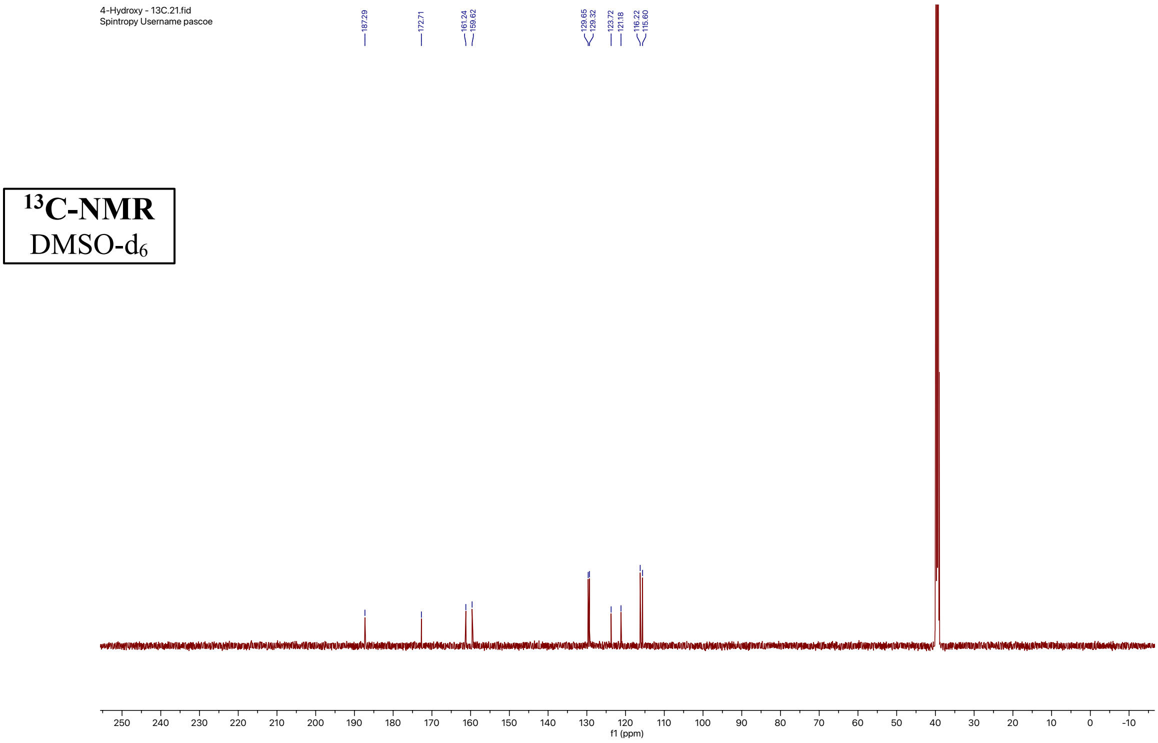

3,5-bis(4-(trifluoromethyl)phenyl)-1,2,4-thiadiazole (10)

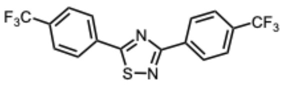

^{1}H-NMR
$CDCl_3$

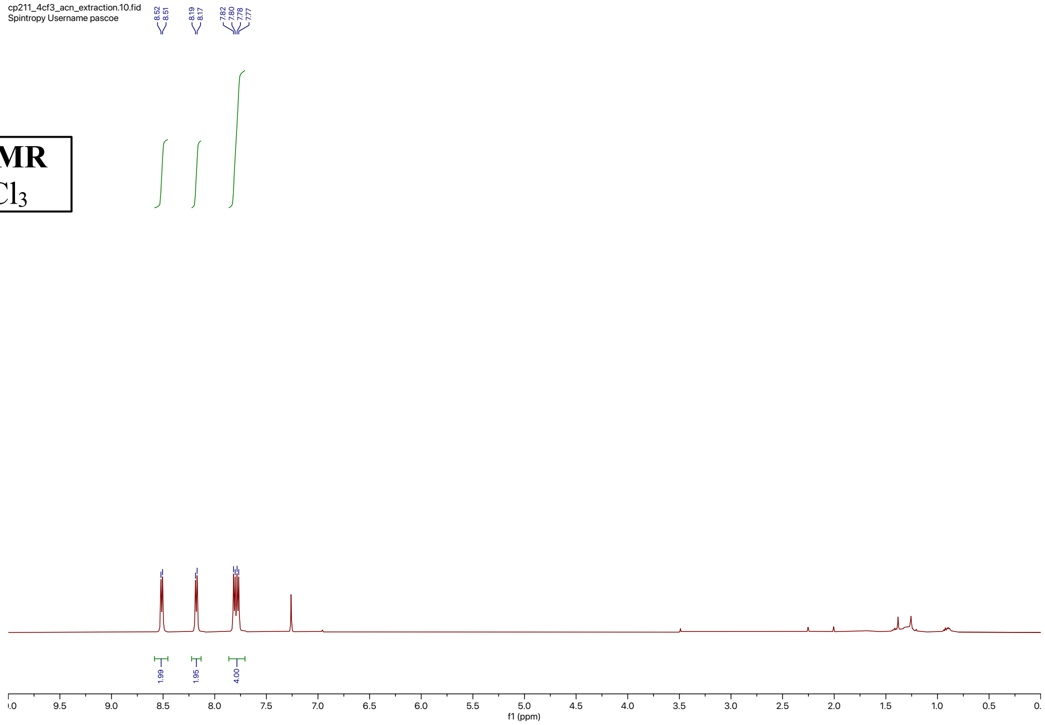

^{13}C-NMR
$CDCl_3$

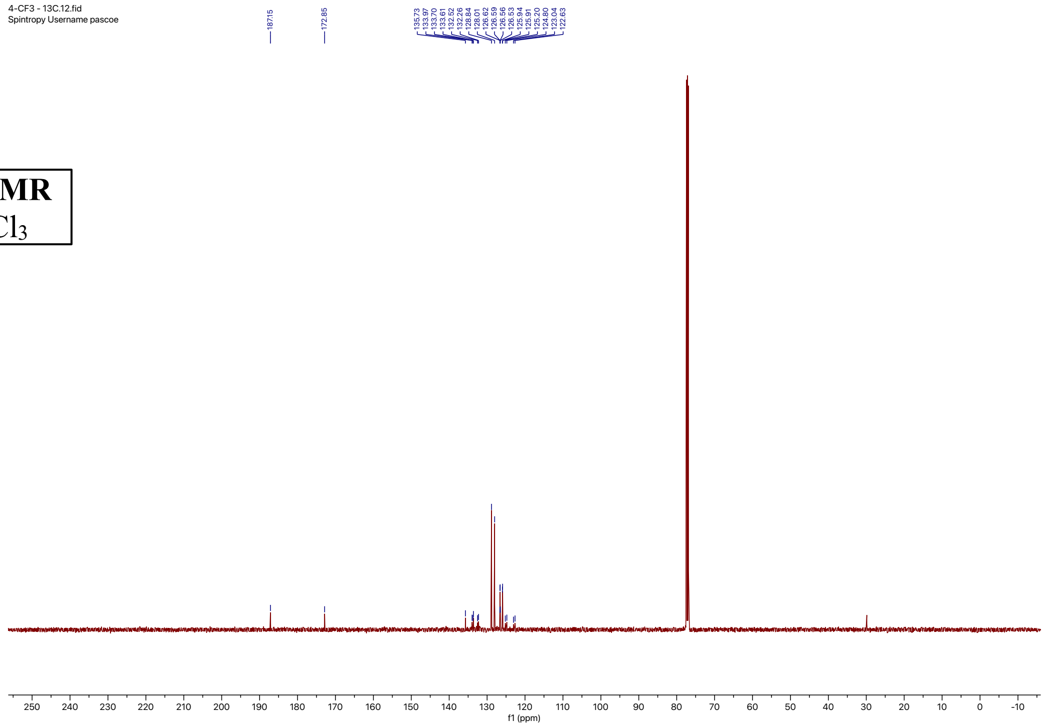

3,5-di-m-tolyl-1,2,4-thiadiazole (11)

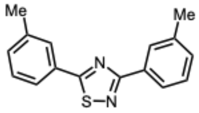

^{1}H-NMR
$CDCl_3$

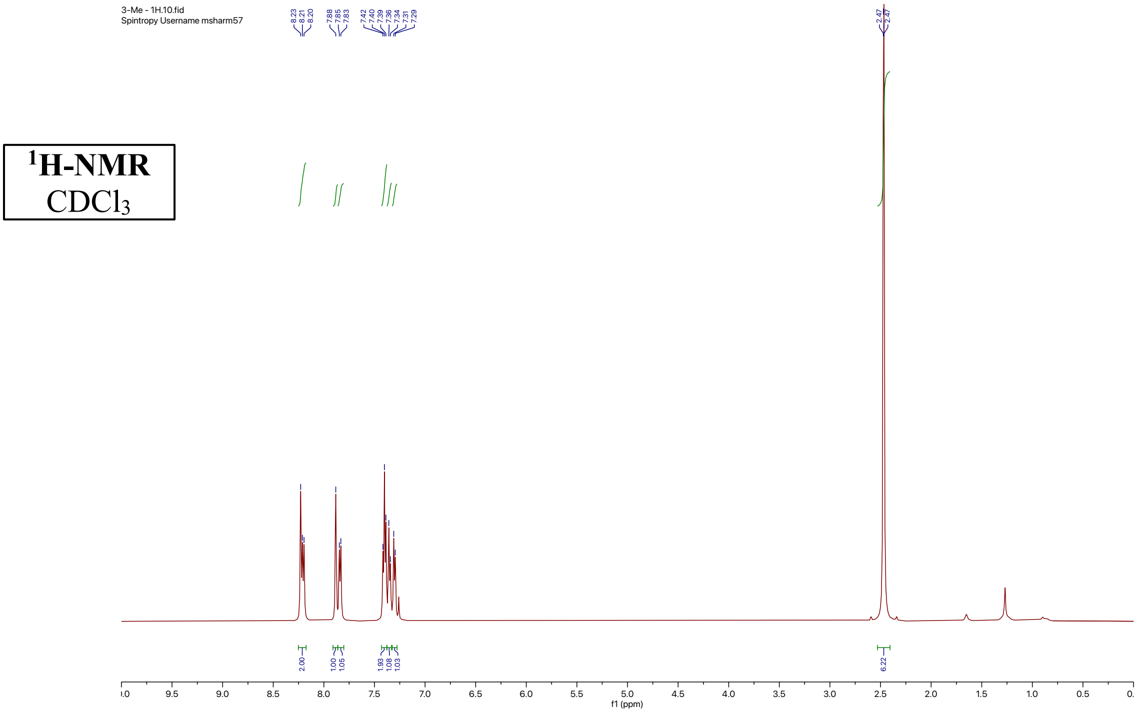

^{13}C-NMR
$CDCl_3$

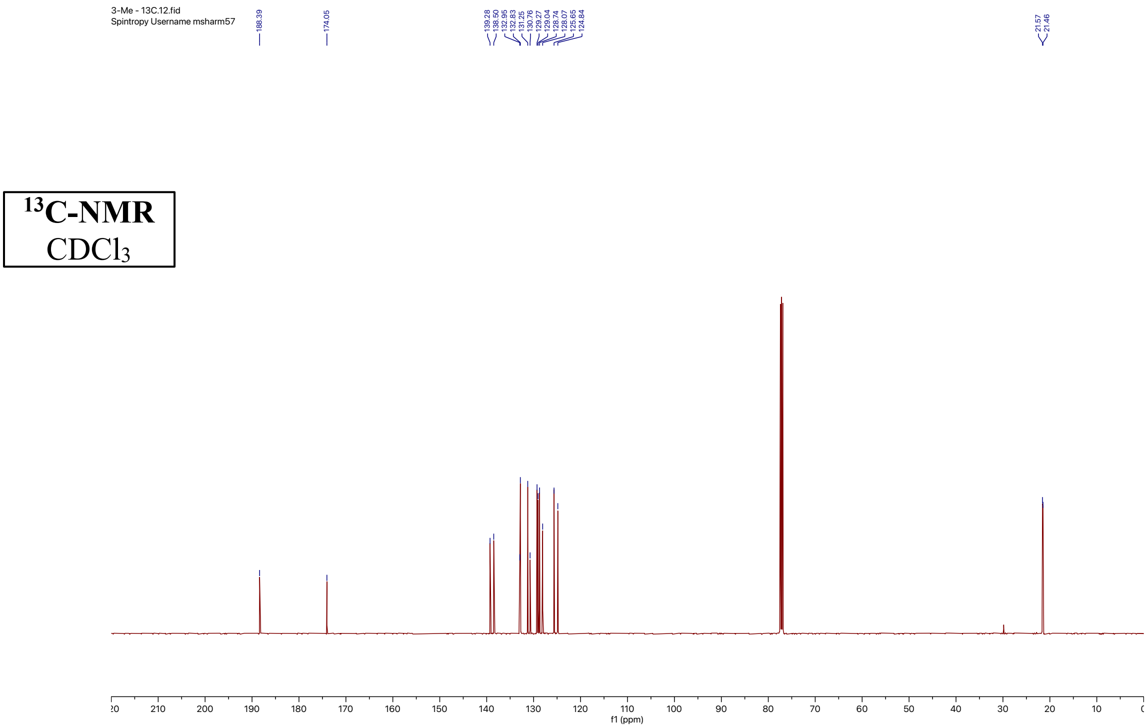

3,5-bis(3-methoxyphenyl)-1,2,4-thiadiazole (12)

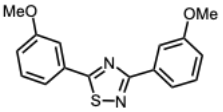

^{1}H-NMR
CDCl$_3$

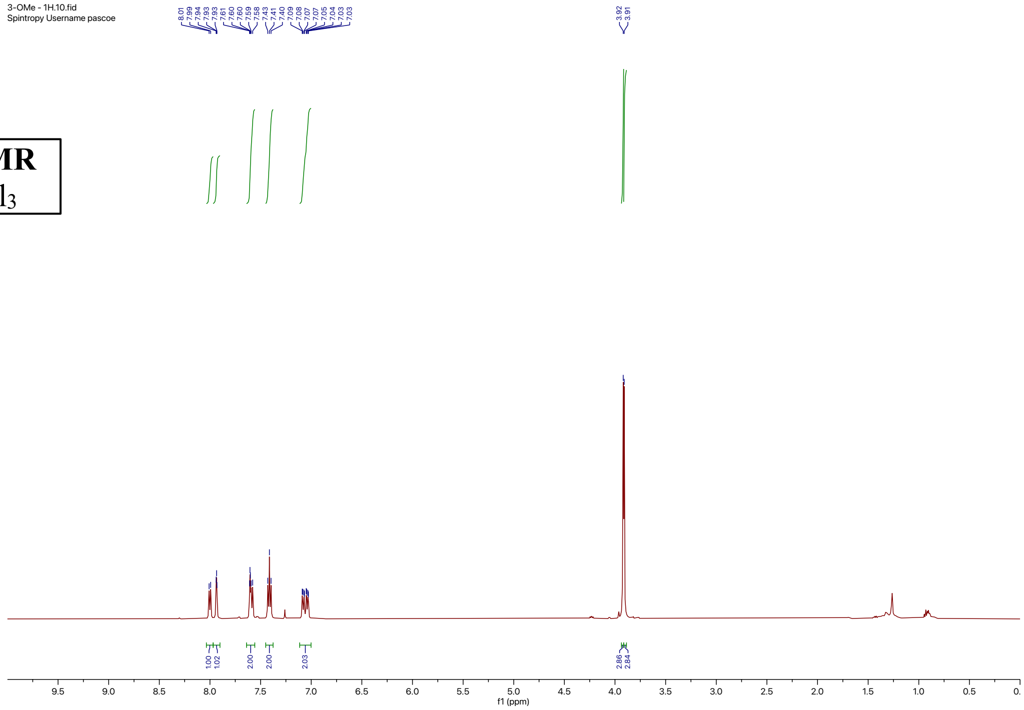

^{13}C-NMR
CDCl$_3$

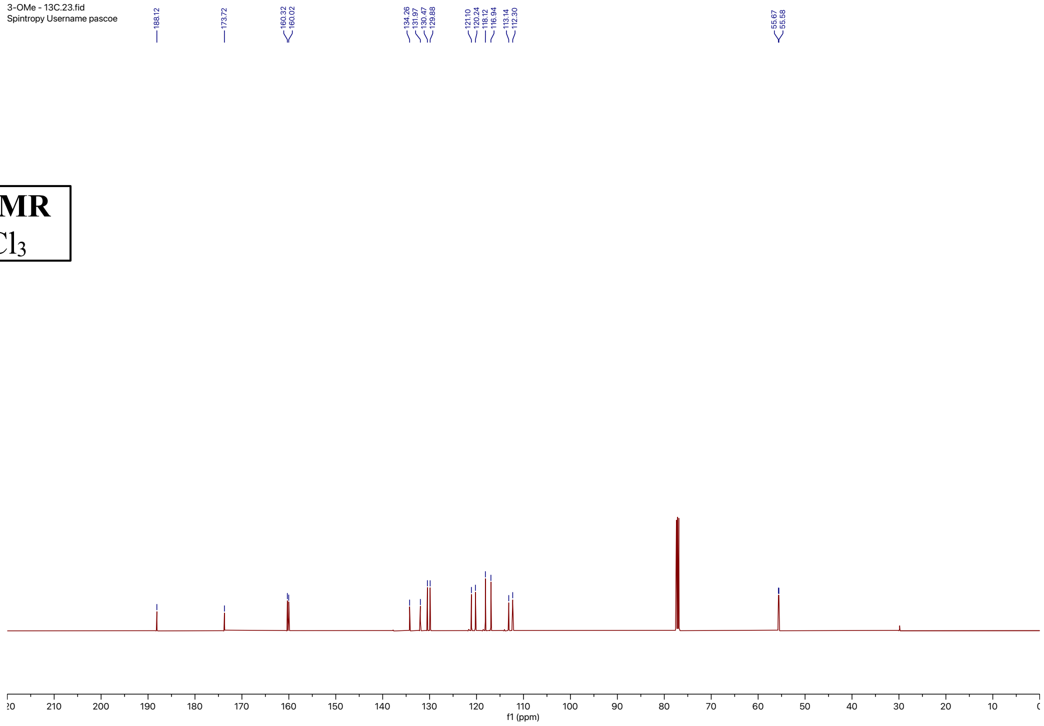

3,5-bis(3-chlorophenyl)-1,2,4-thiadiazole (13)

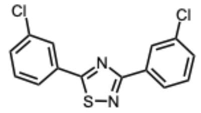

^{1}H-NMR
CDCl3

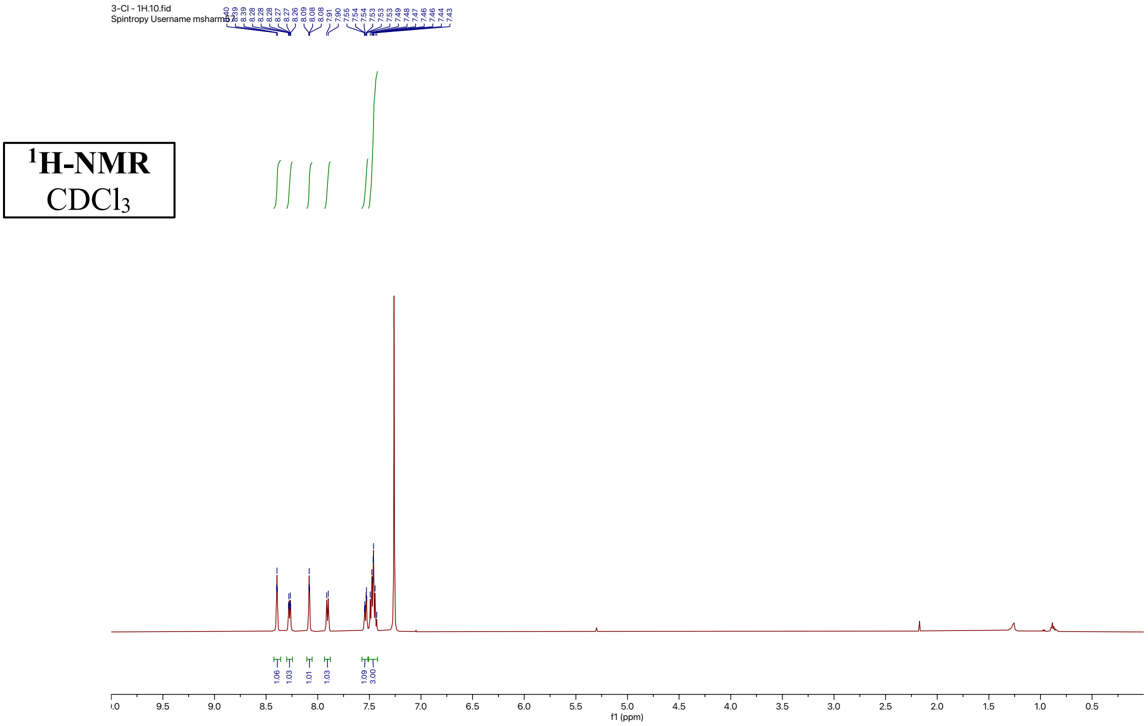

^{13}C-NMR
CDCl3

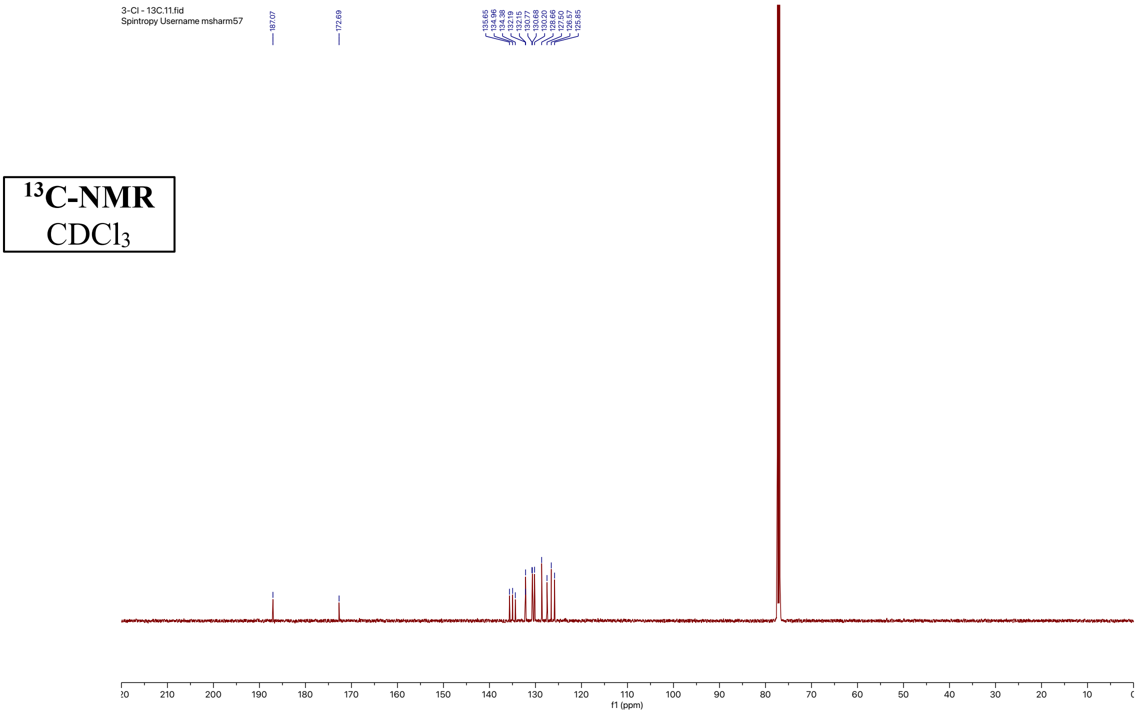

3,5-bis(3-bromophenyl)-1,2,4-thiadiazole (14)

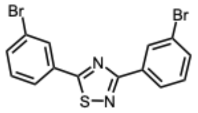

^{1}H-NMR
CDCl$_3$

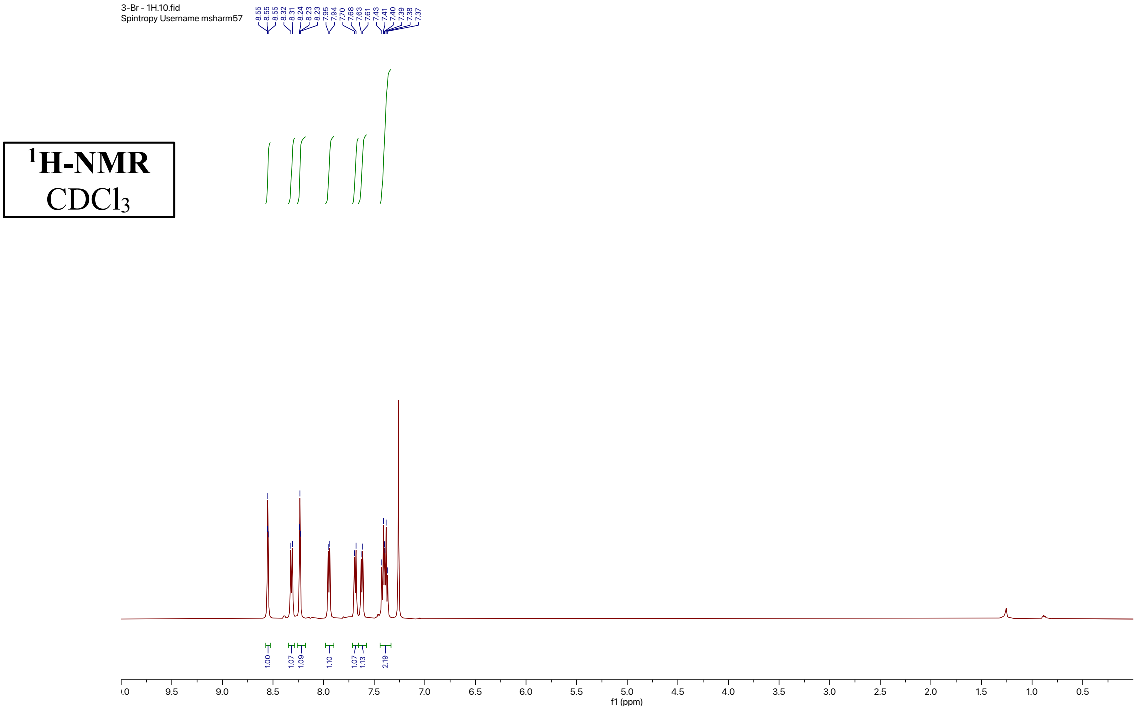

^{13}C-NMR
CDCl$_3$

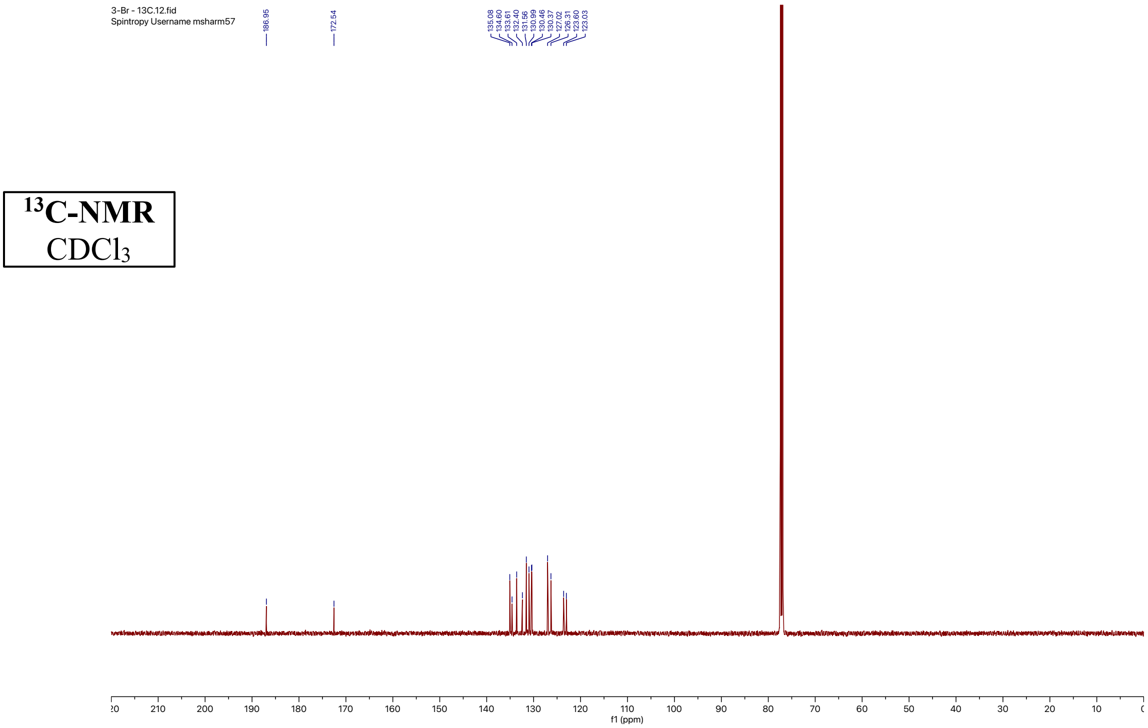

3,5-bis(3-fluorophenyl)-1,2,4-thiadiazole (15)

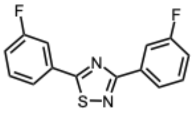

^{1}H-NMR
CDCl$_3$

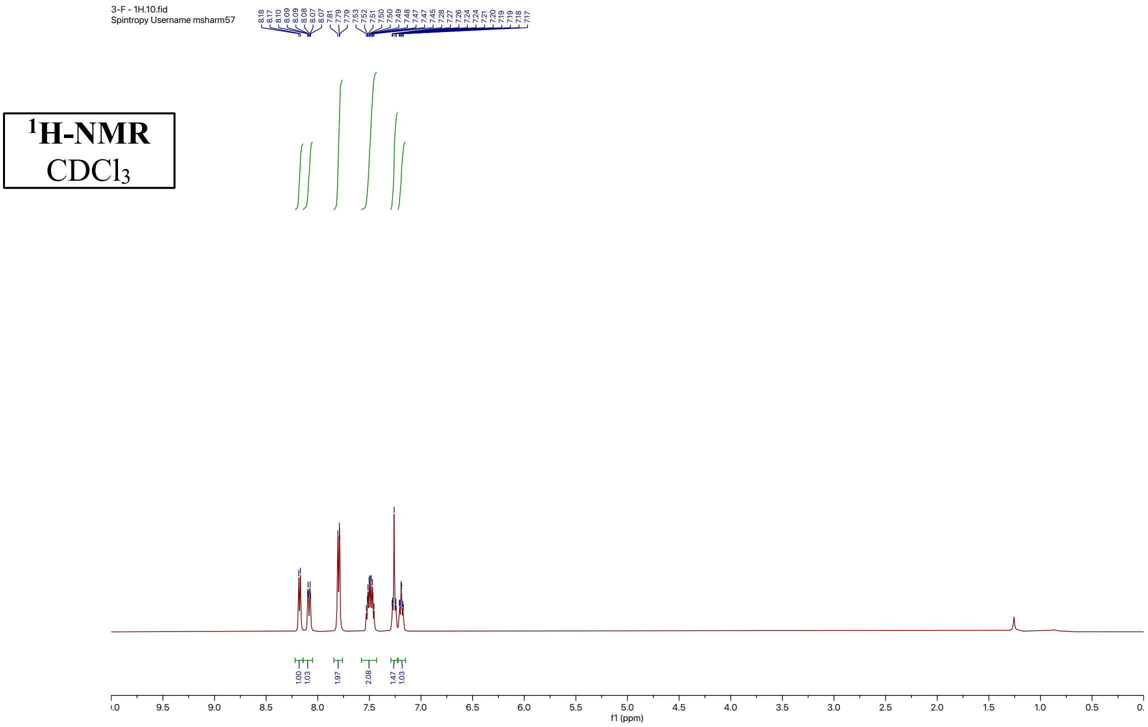

^{13}C-NMR
CDCl$_3$

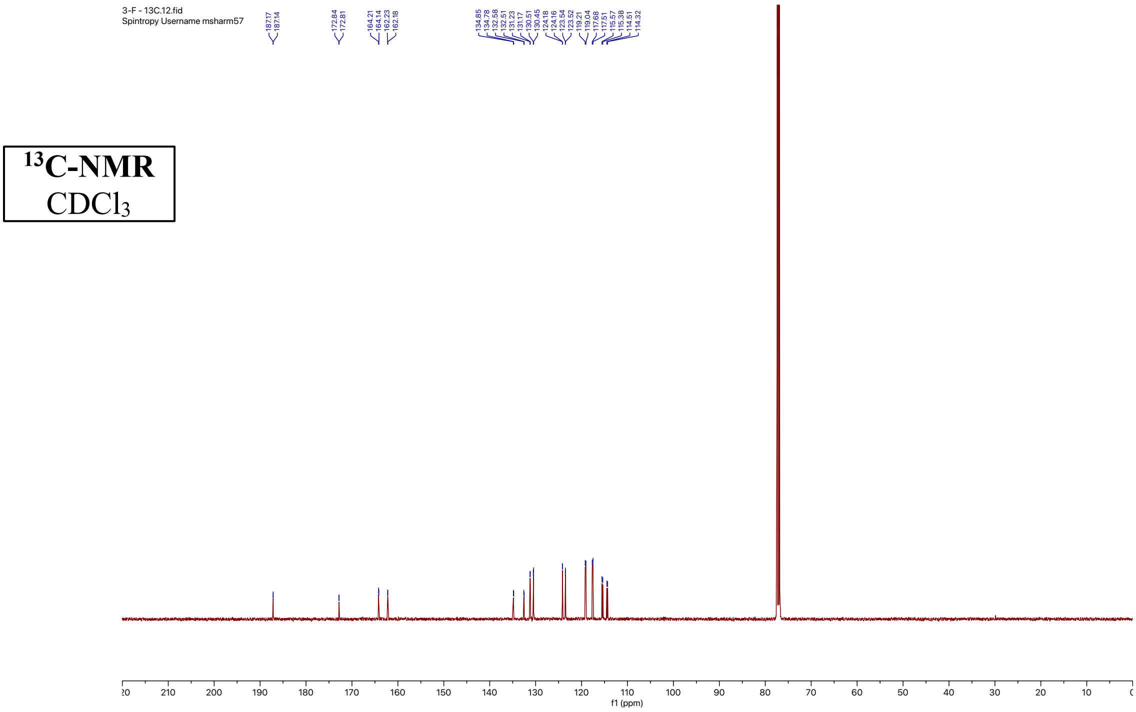

3,5-di-o-tolyl-1,2,4-thiadiazole (16)

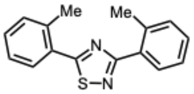

^{1}H-NMR
CDCl$_3$

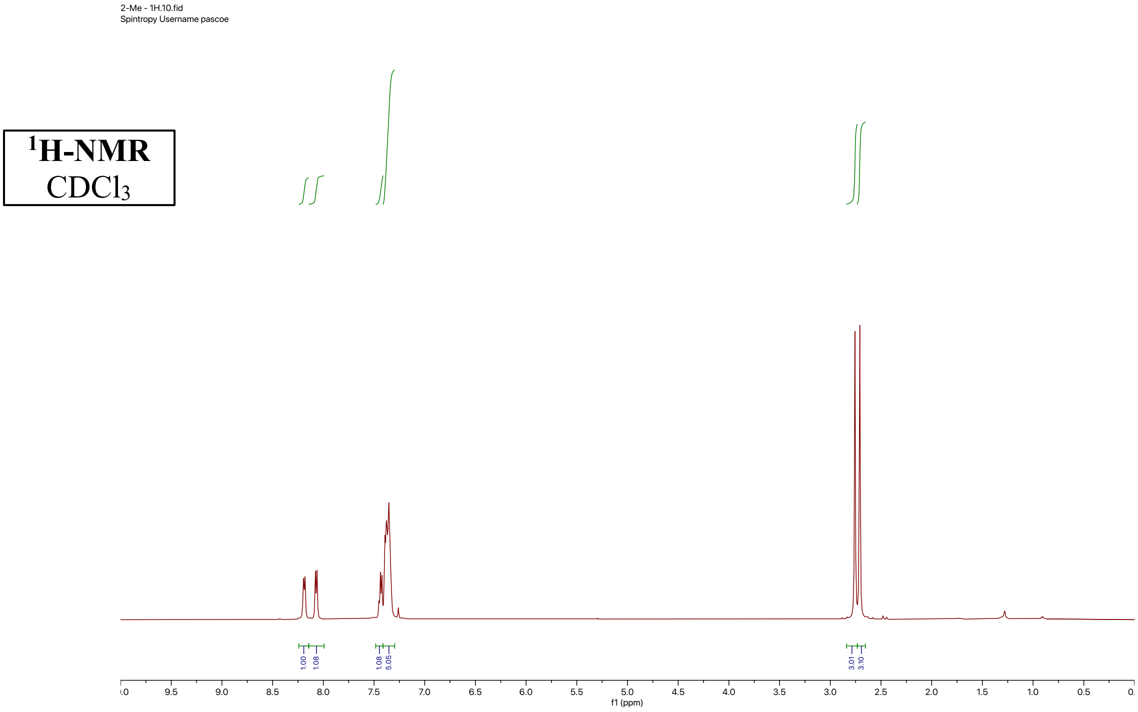

^{13}C-NMR
CDCl$_3$

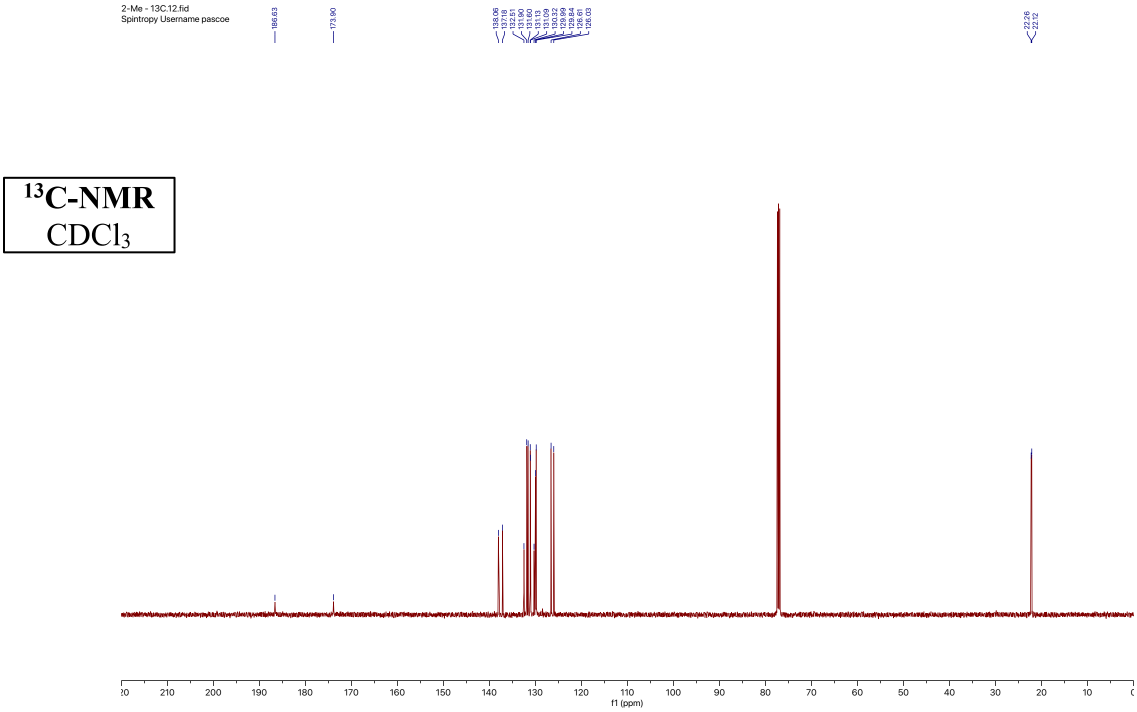

3,5-bis(2-methoxyphenyl)-1,2,4-thiadiazole (17)

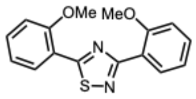

^{1}H-NMR
CDCl$_3$

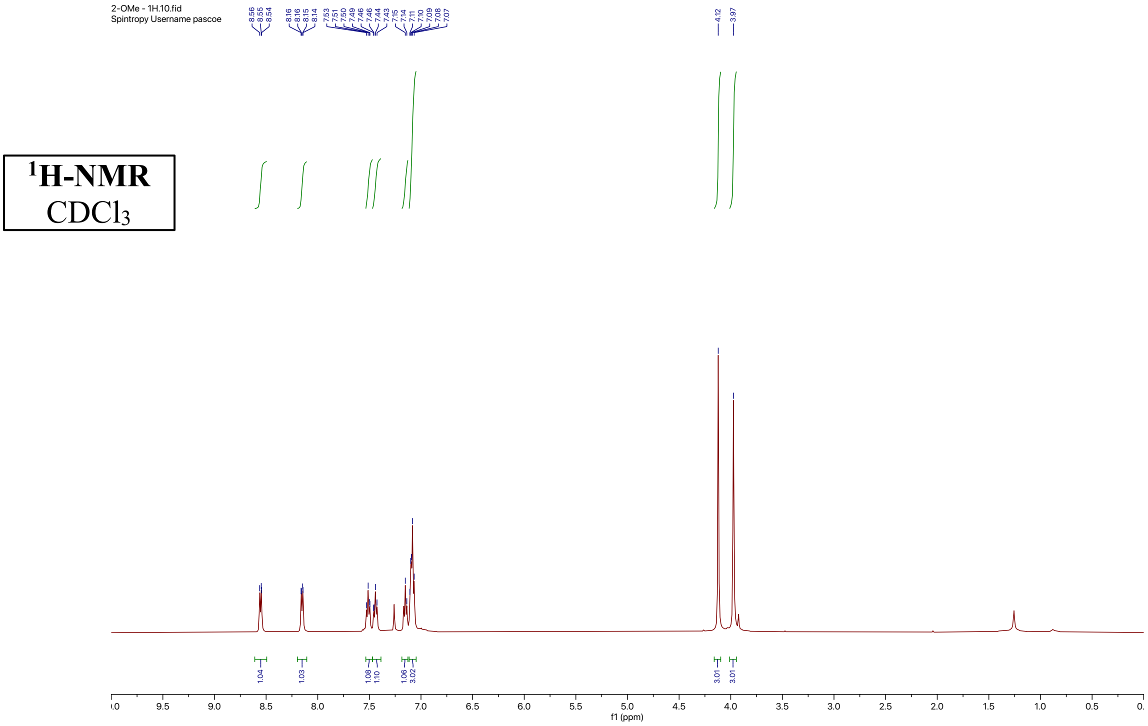

^{13}C-NMR
CDCl$_3$

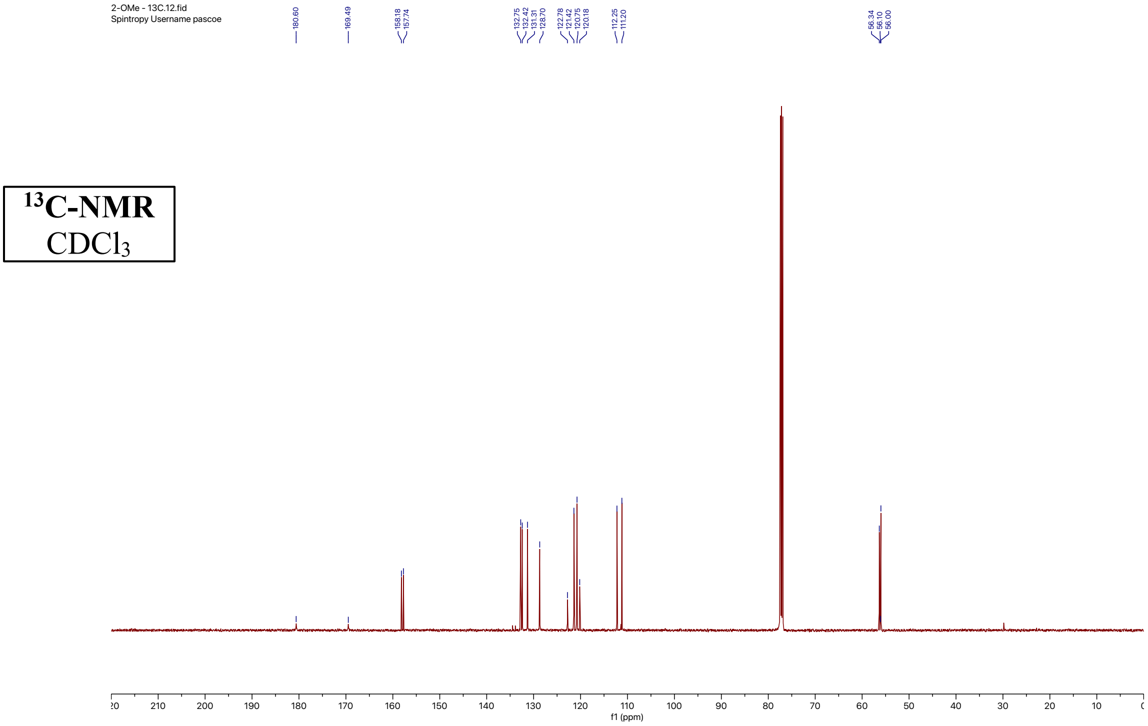

3,5-bis(2-chlorophenyl)-1,2,4-thiadiazole (18)

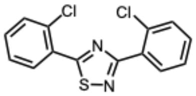

^{1}H-NMR
$CDCl_3$

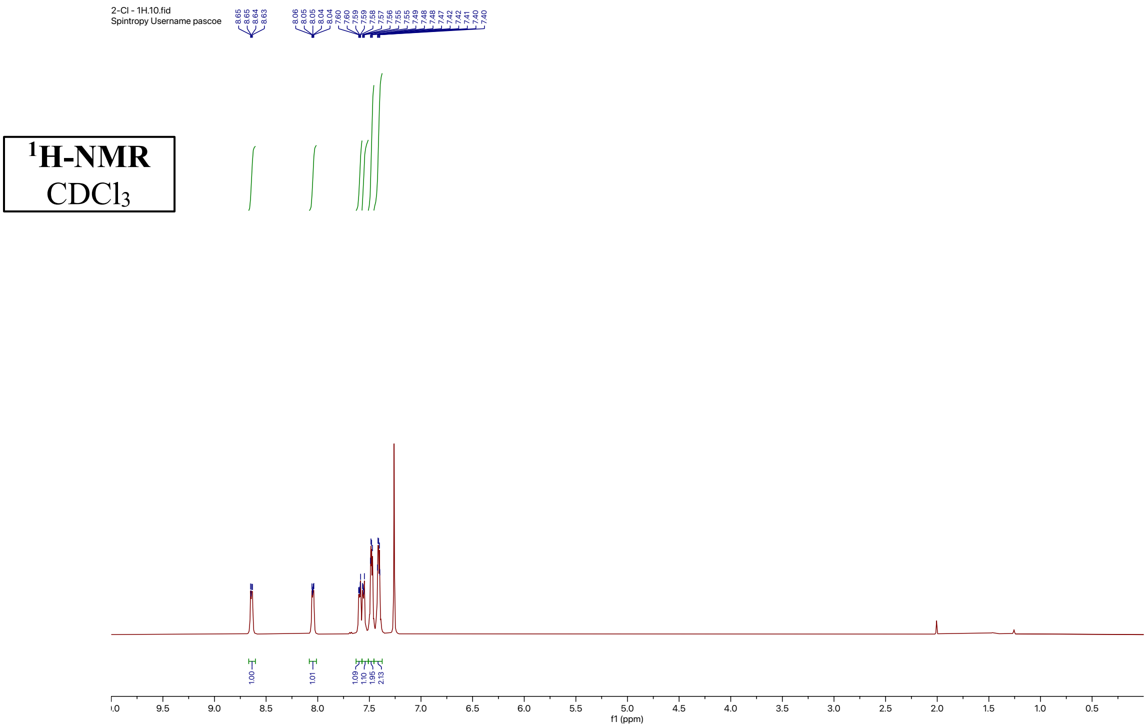

^{13}C-NMR
$CDCl_3$

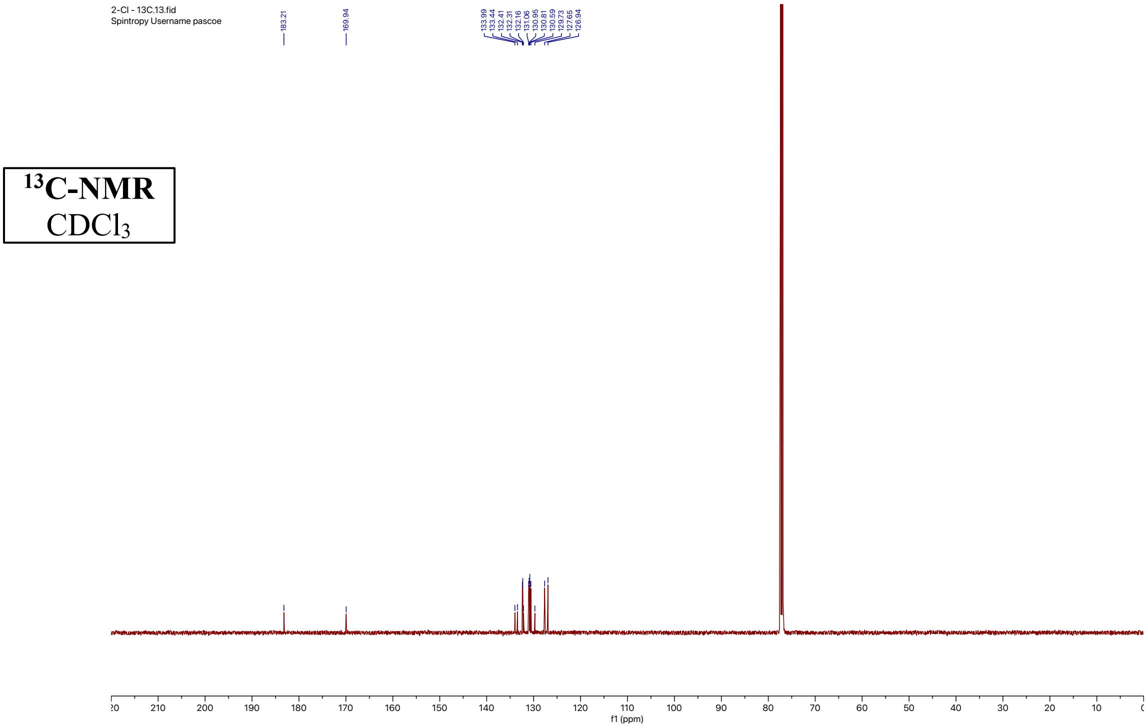

3,5-bis(2-bromophenyl)-1,2,4-thiadiazole (19)

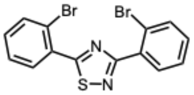

^{1}H-NMR
CDCl$_3$

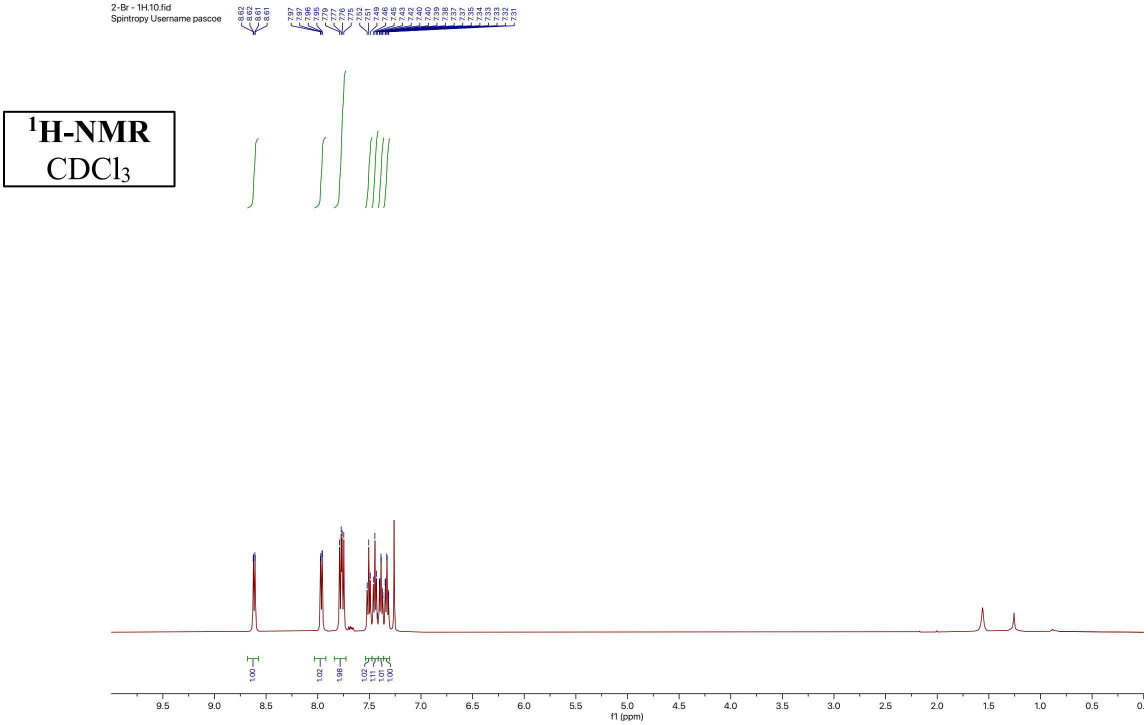

^{13}C-NMR
CDCl$_3$

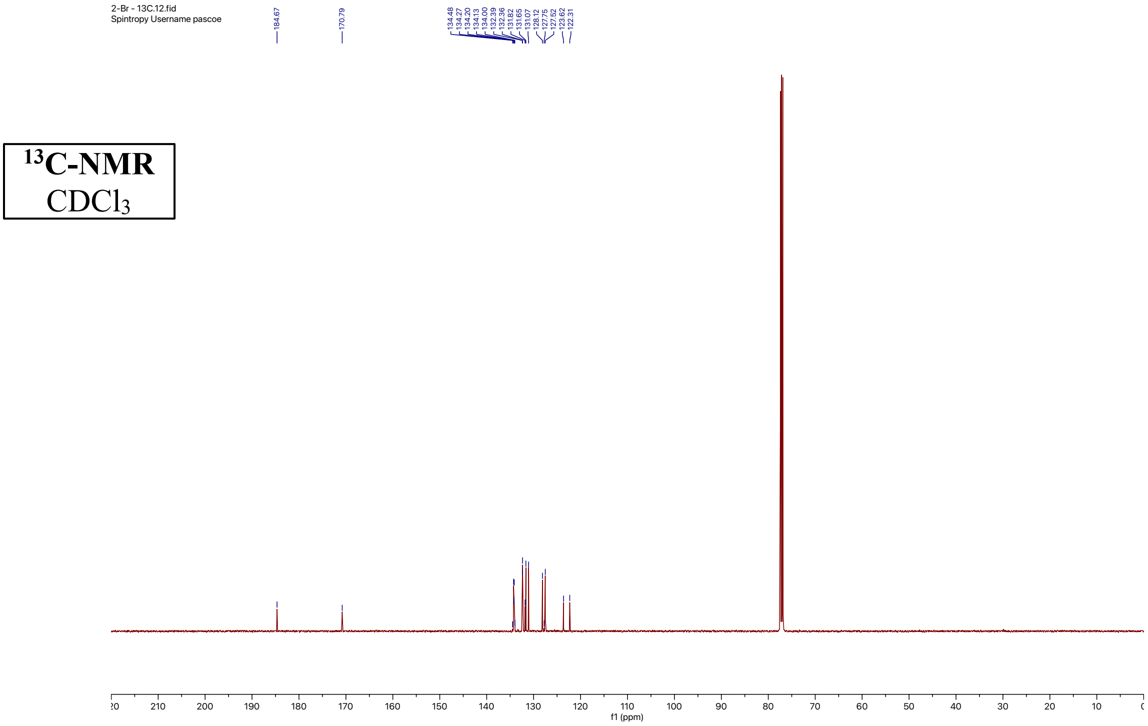

3,5-bis(2-fluorophenyl)-1,2,4-thiadiazole (20)

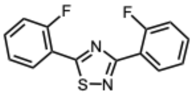

^{1}H-NMR
CDCl$_3$

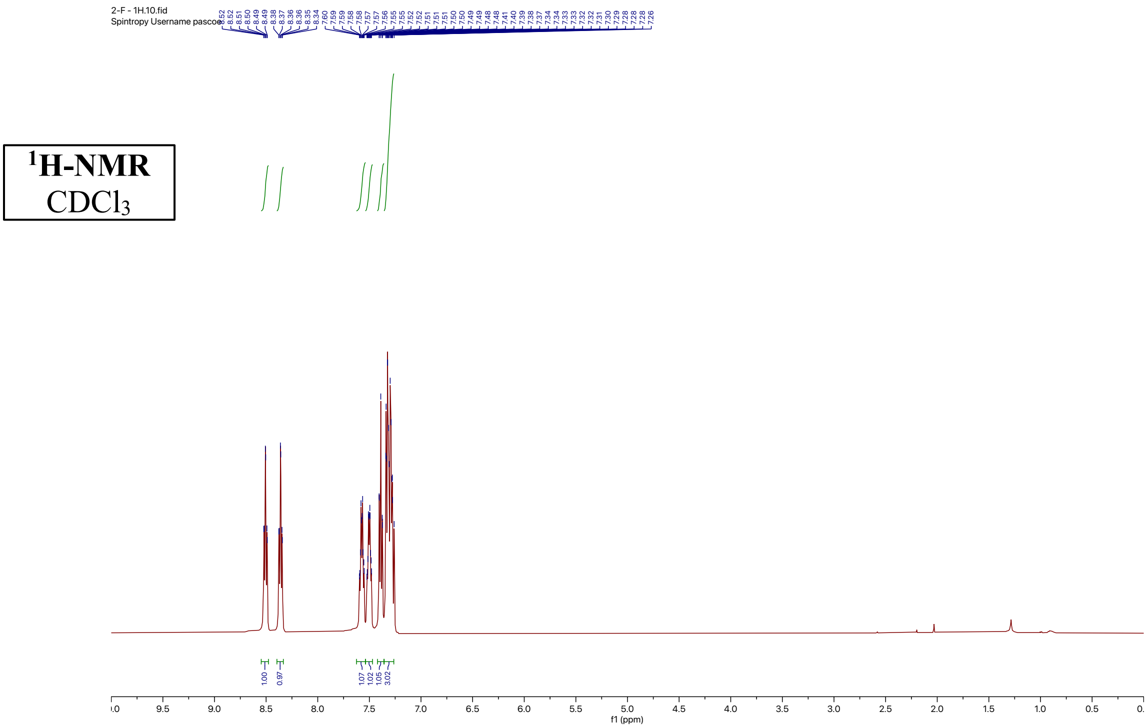

^{13}C-NMR
CDCl$_3$

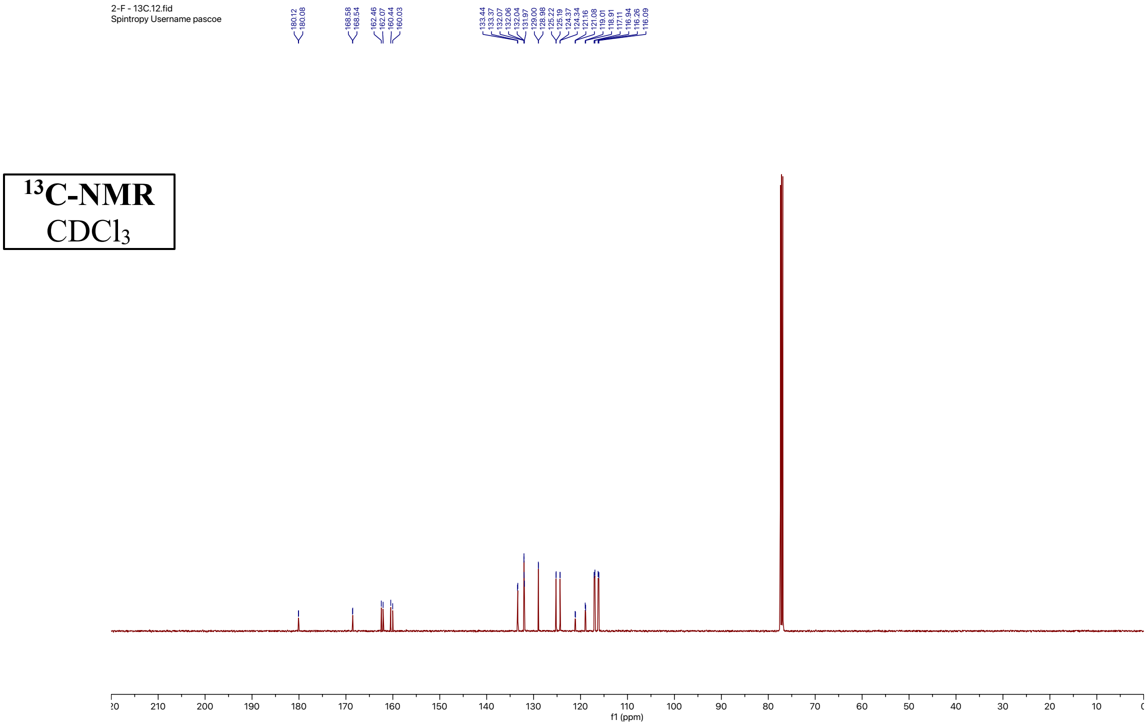

3,5-di(furan-2-yl)-1,2,4-thiadiazole (21)

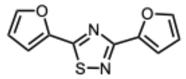

^{1}H-NMR
$CDCl_3$

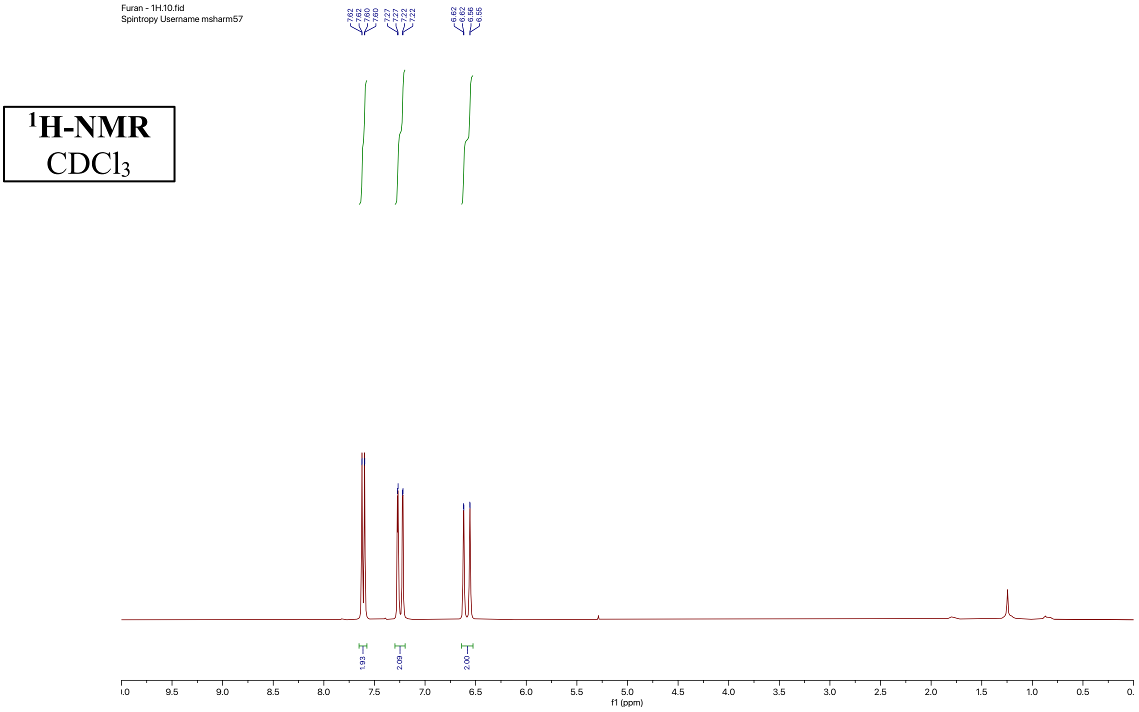

^{13}C-NMR
$CDCl_3$

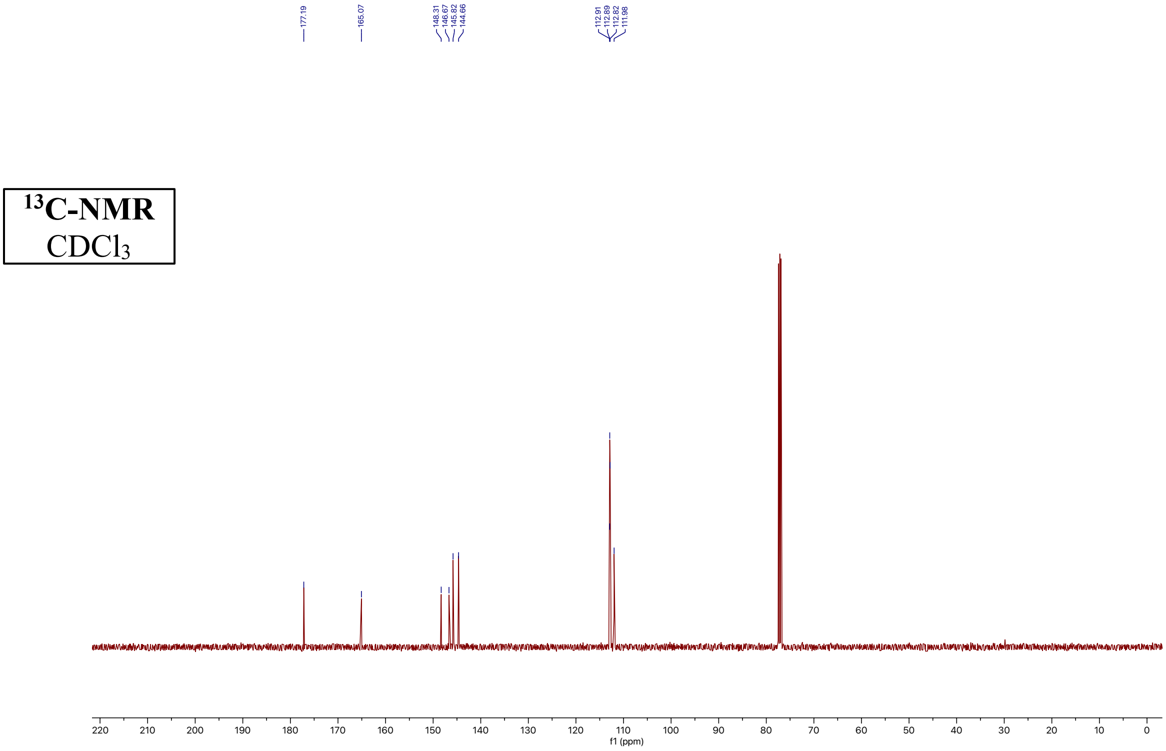

3,5-di(thiophen-2-yl)-1,2,4-thiadiazole (22)

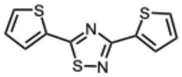

^{1}H-NMR
$CDCl_3$

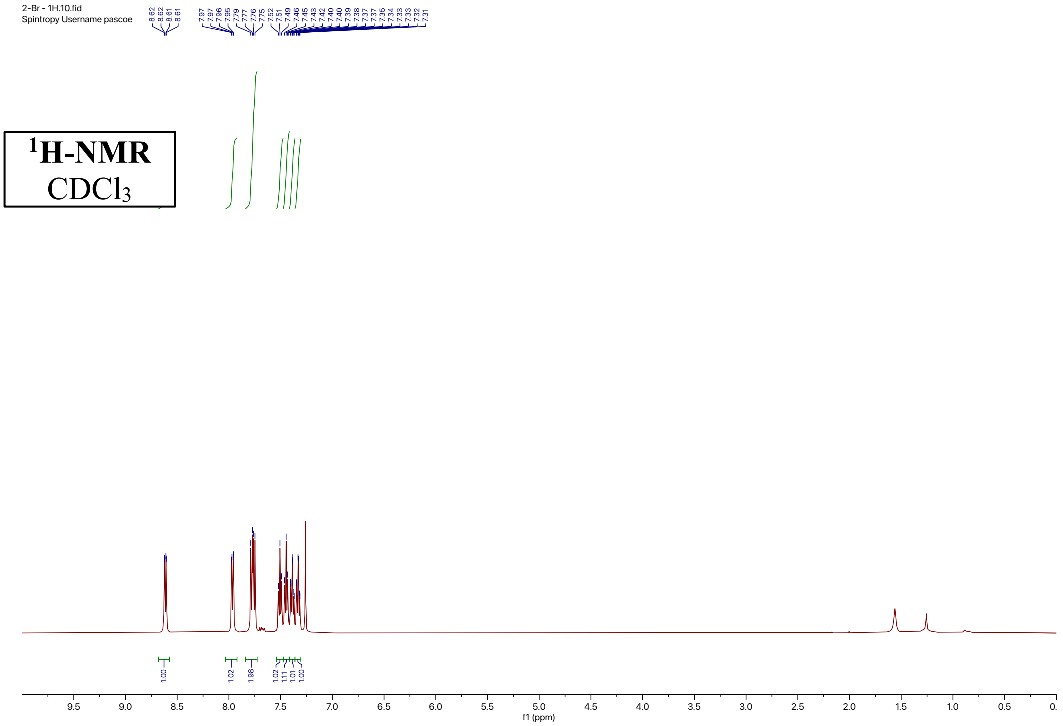

^{13}C-NMR
$CDCl_3$

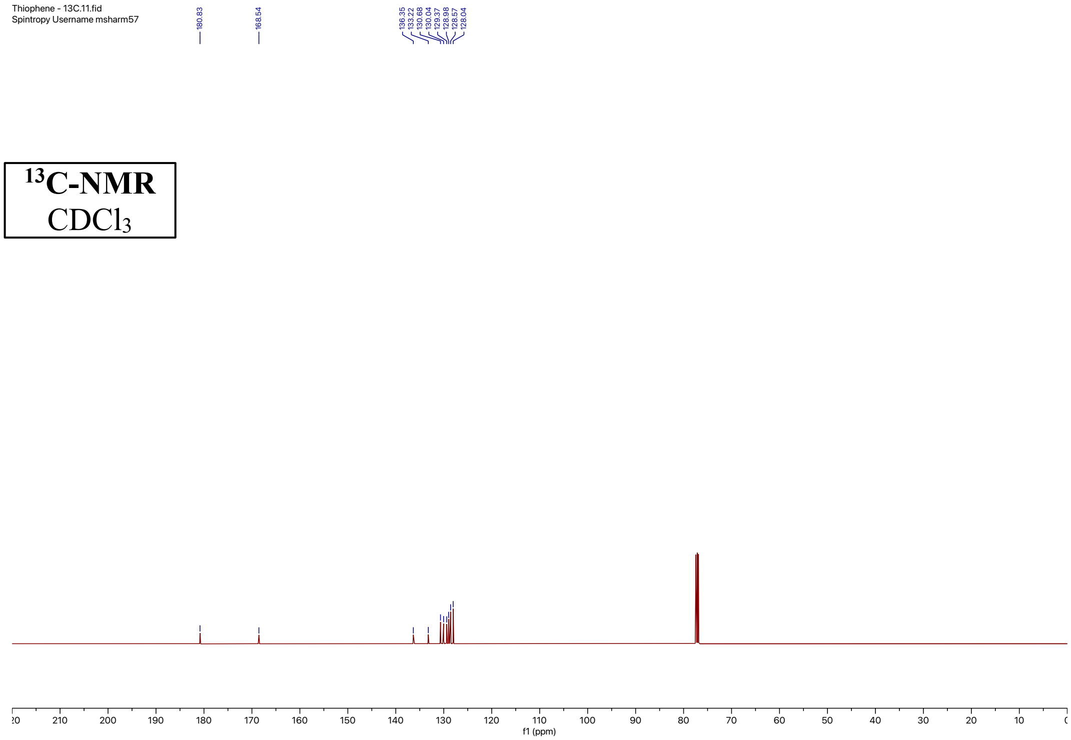

3,5-di(pyridin-2-yl)-1,2,4-thiadiazole (23)

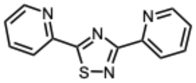

^{1}H-NMR
$CDCl_3$

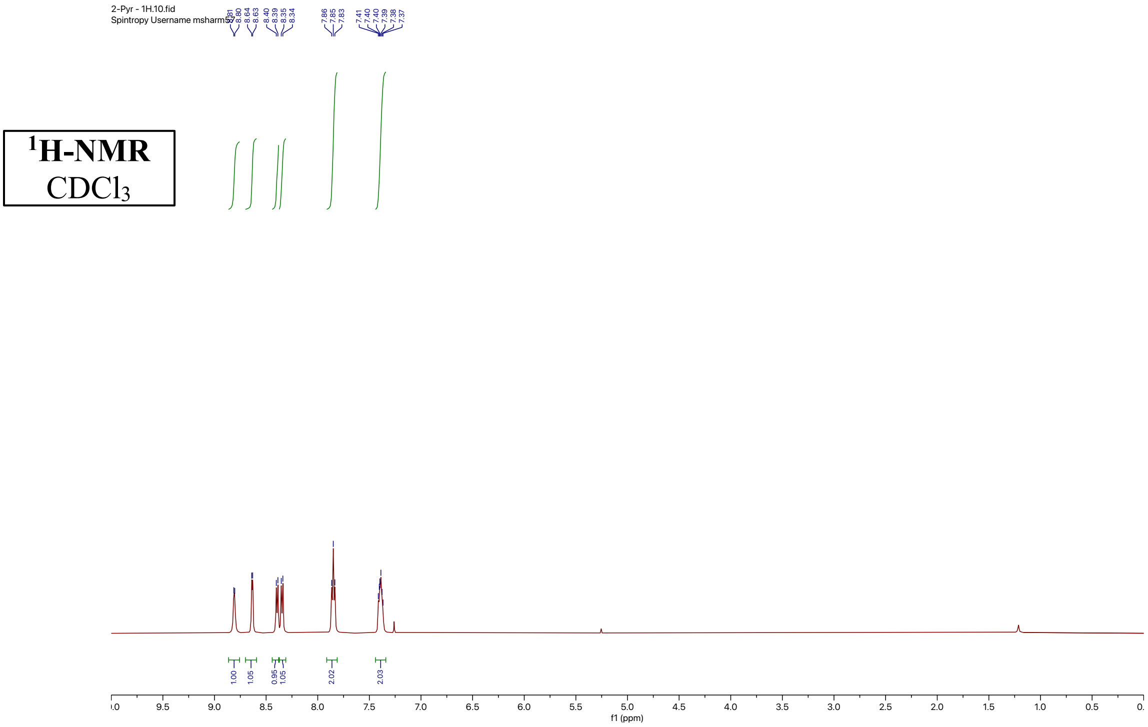

^{13}C-NMR
$CDCl_3$

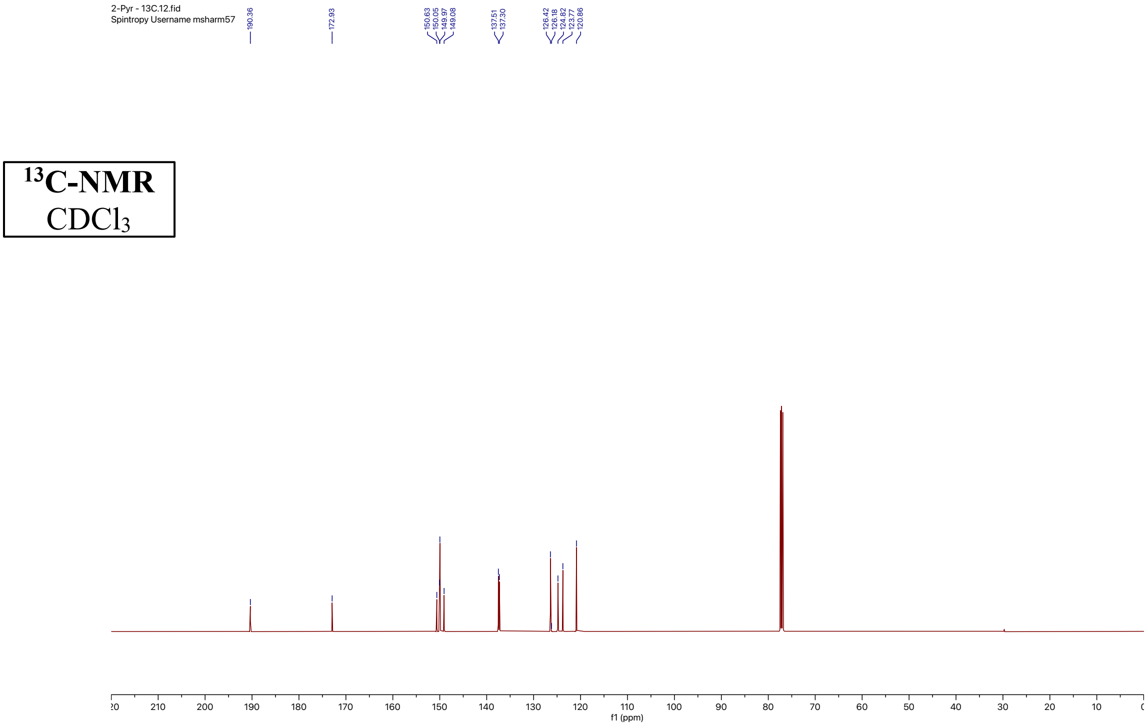

3,5-di(pyridin-4-yl)-1,2,4-thiadiazole (24)

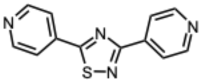

^{1}H-NMR
$CDCl_3$

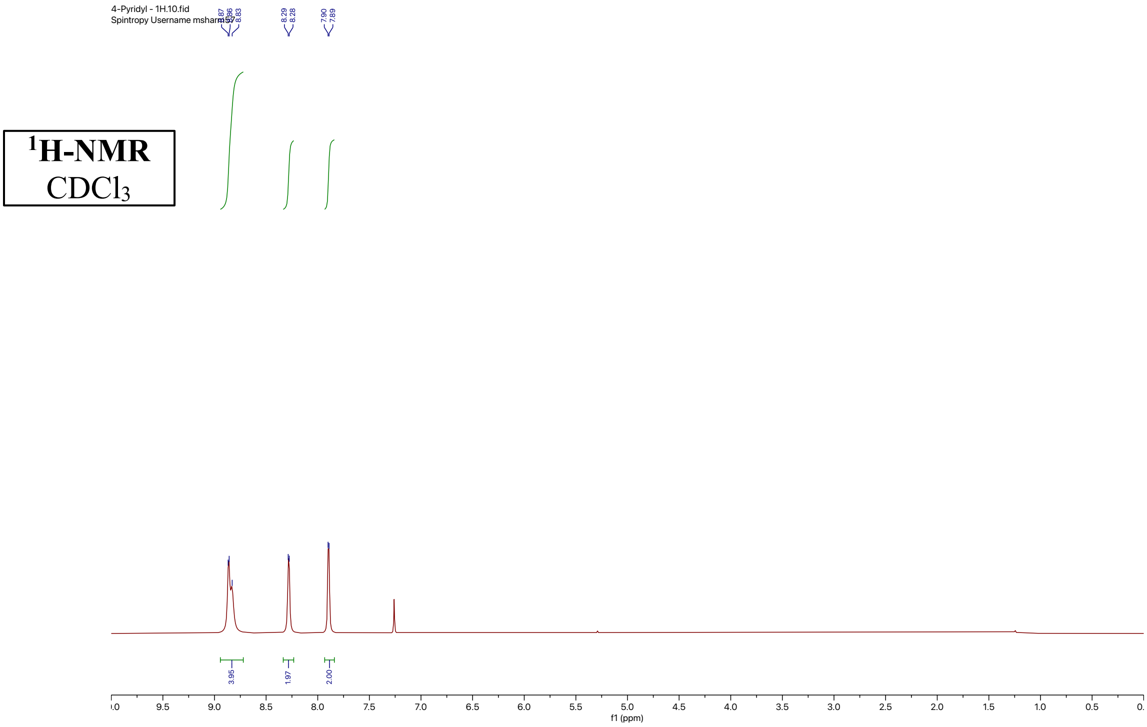

^{13}C-NMR
$CDCl_3$

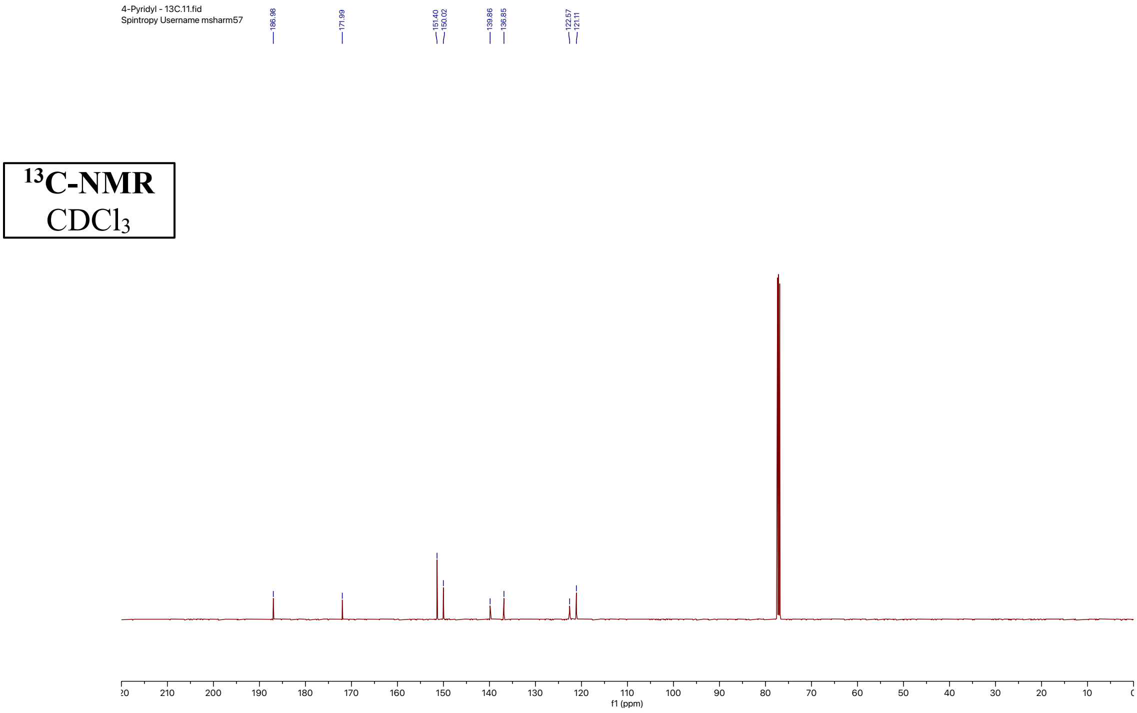

4,4'-(1,2,4-thiadiazole-3,5-diyl)dimorpholine (25)

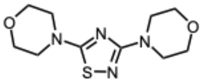

^{1}H-NMR
CDCl$_3$

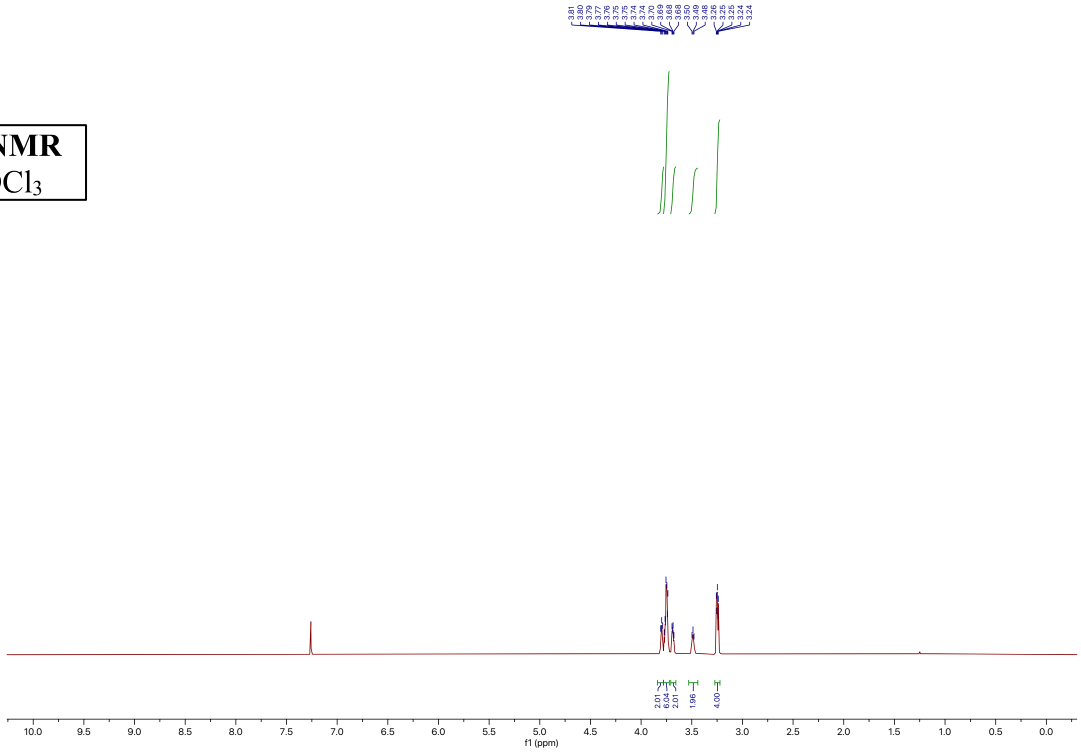

^{13}C-NMR
CDCl$_3$

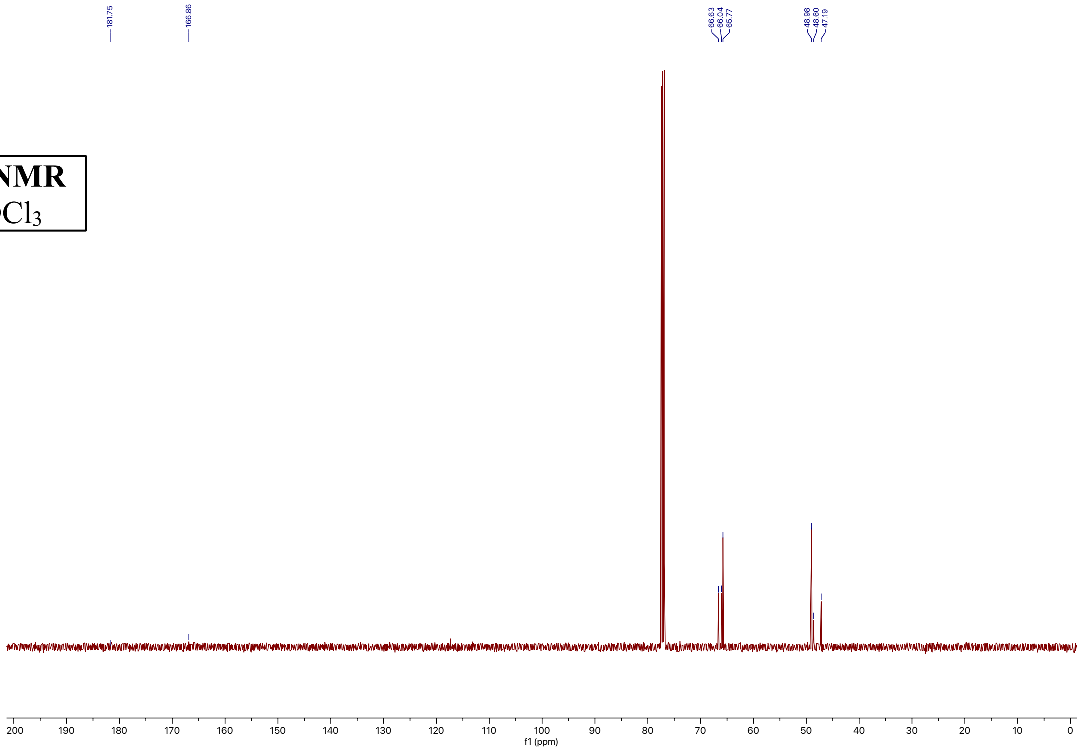

3,5-bis(4-methoxybenzyl)-1,2,4-thiadiazole (26)

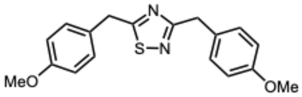

^{1}H-NMR
CDCl$_3$

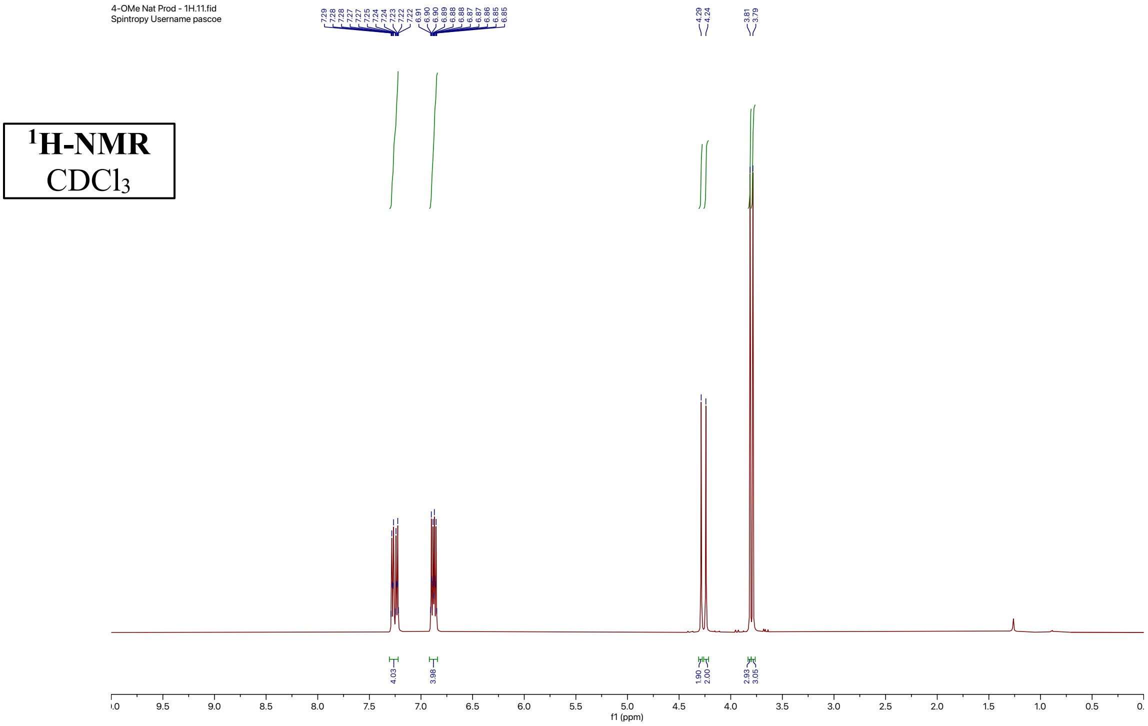

^{13}C-NMR
CDCl$_3$

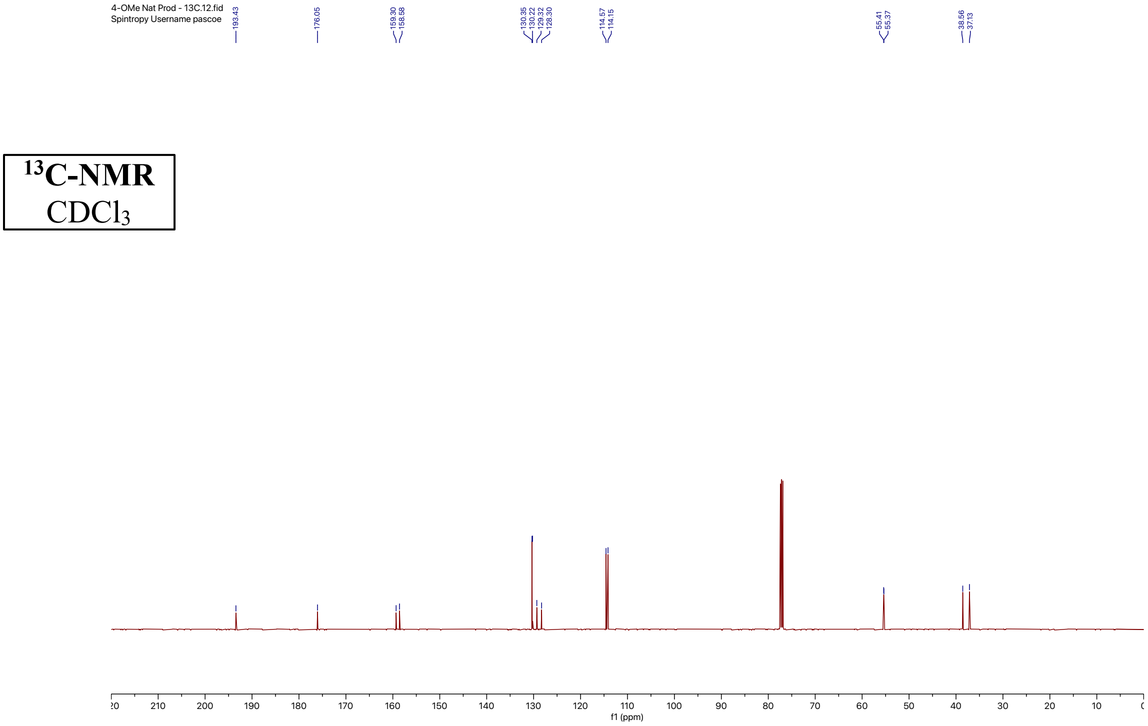

N-(imino(phenyl)methyl)benzothioamide (29)

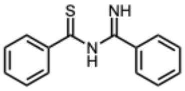

^{1}H-NMR
DMSO-d_6

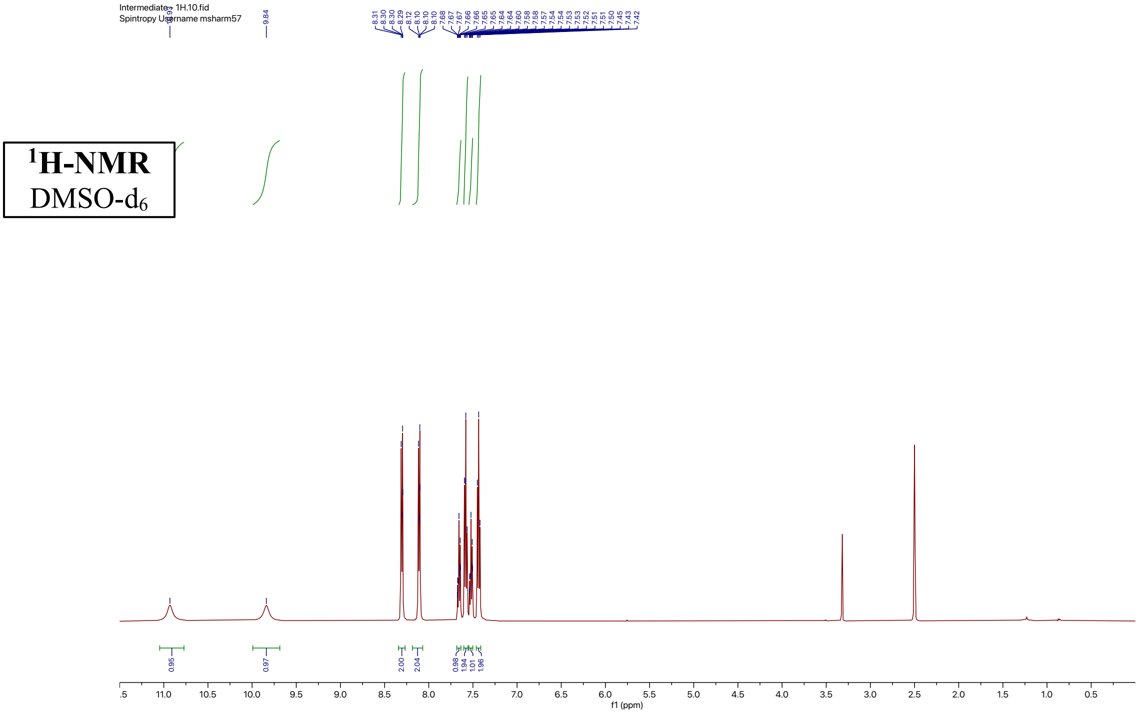

^{13}C-NMR
DMSO-d_6

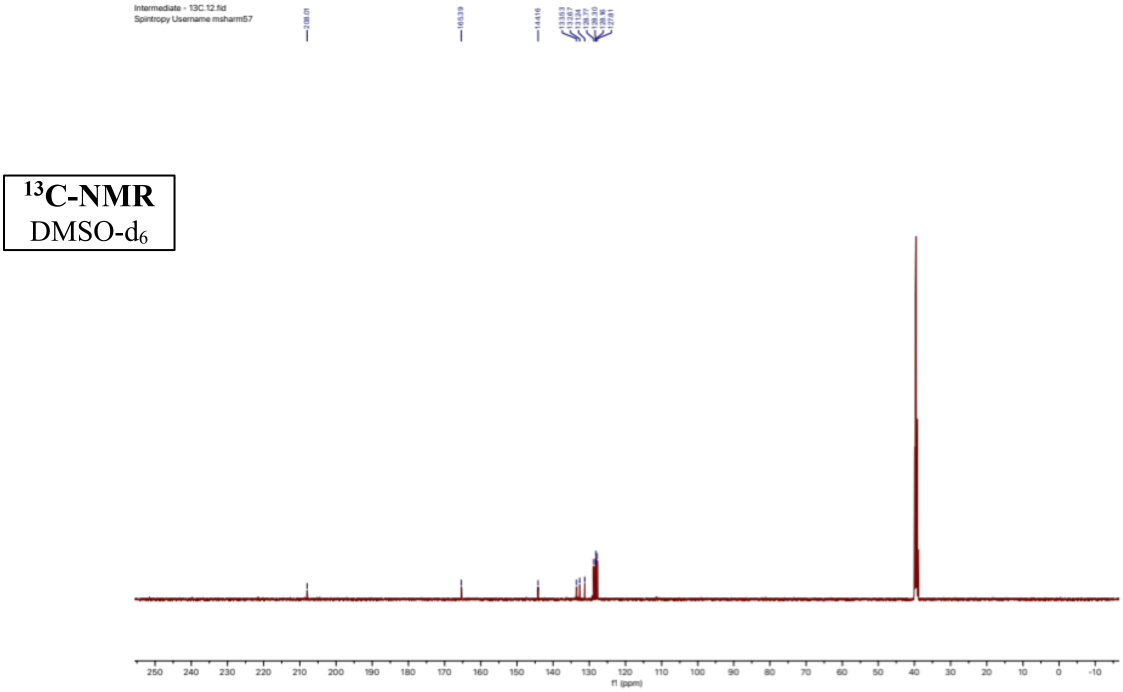

2-(4-hydroxyphenyl)ethanethioamide (34)

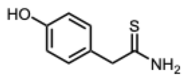

^{1}H-NMR
DMSO-d_6

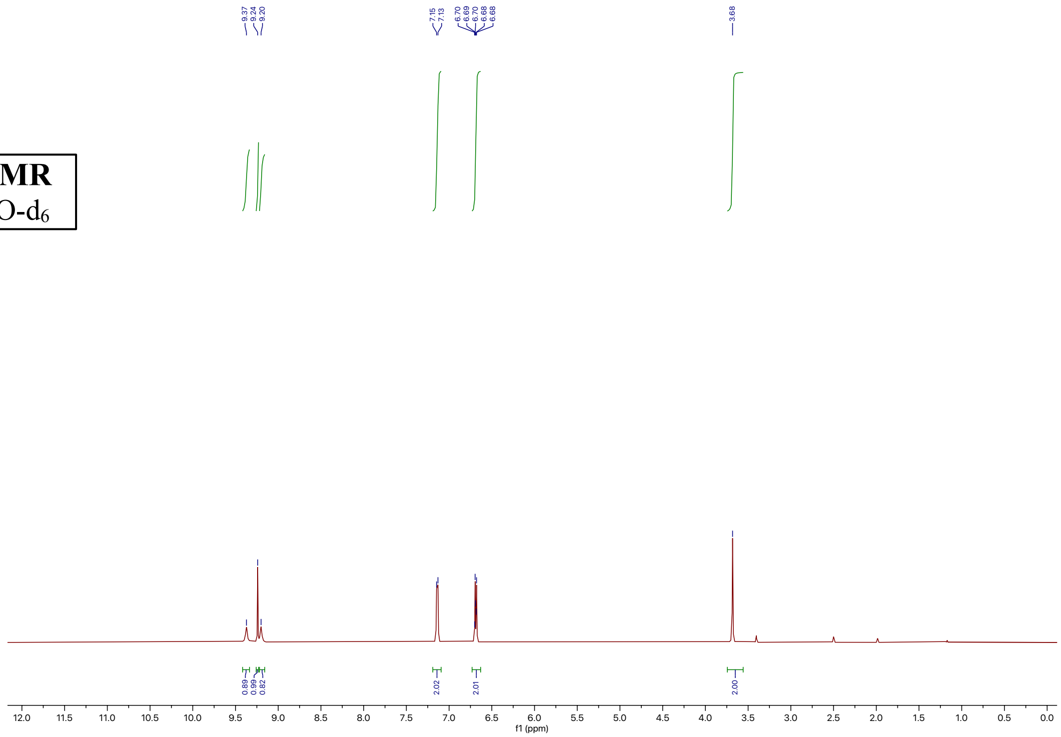

^{13}C-NMR
DMSO-d_6

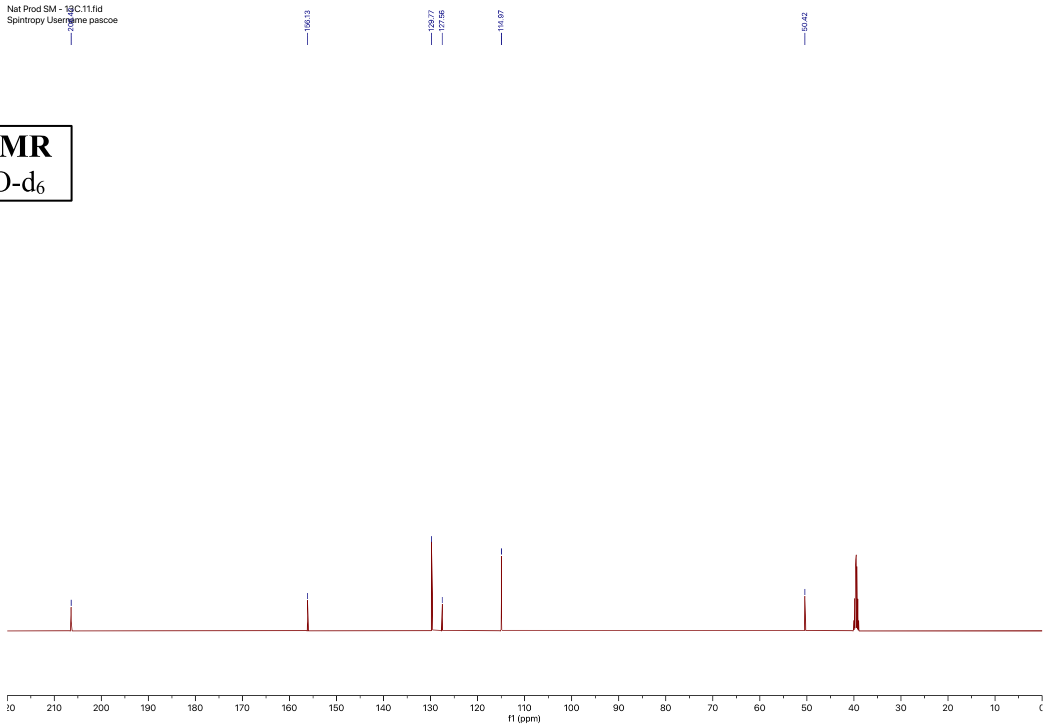

4,4'-((1,2,4-thiadiazole-3,5-diyl)bis(methylene))diphenol (35)

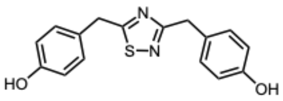

^{1}H-NMR
DMSO-d_6

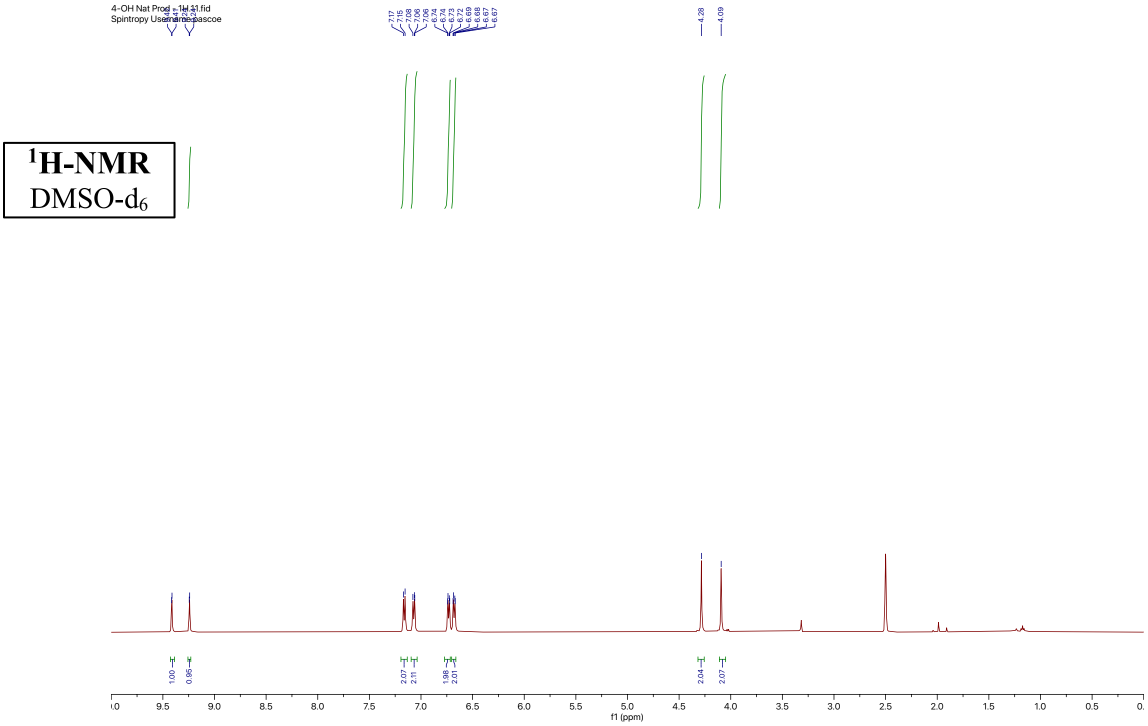

^{13}C-NMR
DMSO-d_6

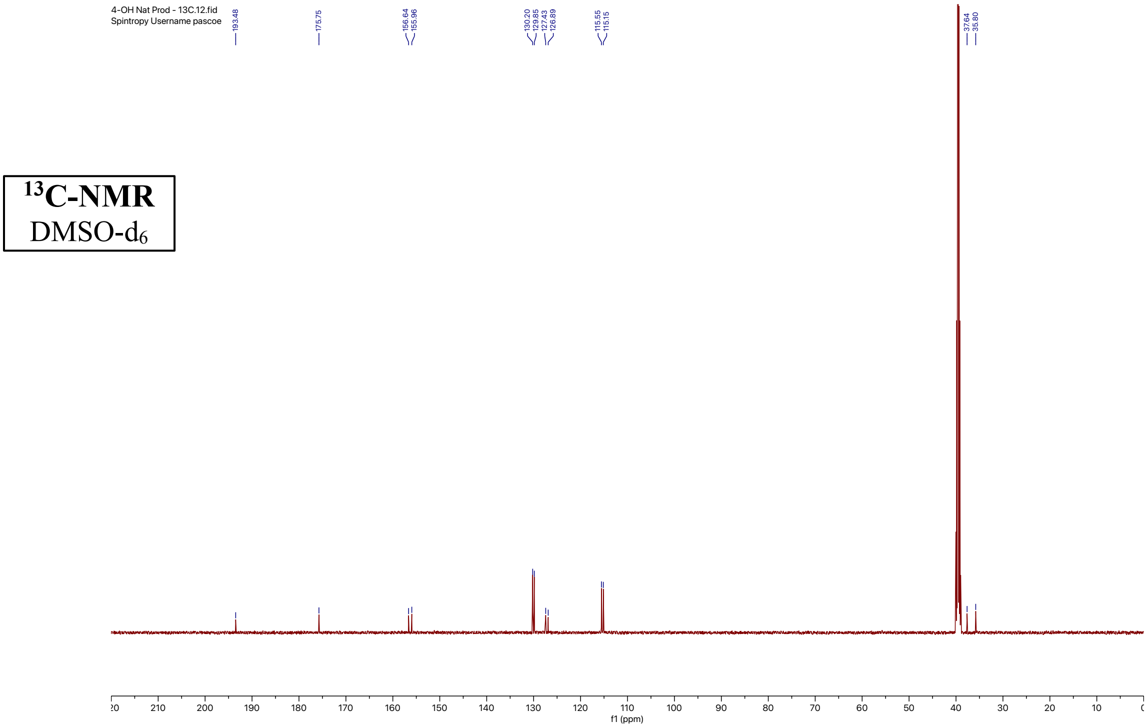

Supplement: Supplementary file 1 — ja5c01175_si_001.pdf [file ja5c01175_si_001.pdf]
